# Ancient genomes from northern China suggest links between subsistence changes and human migration

**DOI:** 10.1038/s41467-020-16557-2

**Published:** 2020-06-01

**Authors:** Chao Ning, Tianjiao Li, Ke Wang, Fan Zhang, Tao Li, Xiyan Wu, Shizhu Gao, Quanchao Zhang, Hai Zhang, Mark J. Hudson, Guanghui Dong, Sihao Wu, Yanming Fang, Chen Liu, Chunyan Feng, Wei Li, Tao Han, Ruo Li, Jian Wei, Yonggang Zhu, Yawei Zhou, Chuan-Chao Wang, Shengying Fan, Zenglong Xiong, Zhouyong Sun, Maolin Ye, Lei Sun, Xiaohong Wu, Fawei Liang, Yanpeng Cao, Xingtao Wei, Hong Zhu, Hui Zhou, Johannes Krause, Martine Robbeets, Choongwon Jeong, Yinqiu Cui

**Affiliations:** 1 School of Life Sciences, Jilin University, Changchun, 130012 China; 2 Max Planck Institute for the Science of Human History, 07745 Jena, Germany; 3 Department of Archaeology, College of History, Wuhan University, Wuhan, 430072 China; 4 College of Pharmacia Sciences, Jilin University, Changchun, 130021 China; 5 Research Center for Chinese Frontier Archaeology of Jilin University, Jilin University, Changchun, 130012 China; 6 School of Archaeology and Museology, Peking University, Beijing, 100871 China; 7 MOE Key Laboratory of Western China’s Environmental System, College of Earth & Environmental Sciences, Lanzhou University, Lanzhou, 730000 China; 8 Henan Provincial Institute of Cultural Heritage and Archaeology, Zhengzhou, 450000 China; 9 Luohe Municipal Institute of Cultural Relics, Luohe, 462000 China; 10 Jiaozuo Municipal Institute of Cultural Relics, Jiaozuo, 454000 China; 11 School of History, Renmin University of China, Beijing, 100872 China; 12 School of History, Zhengzhou University, Zhengzhou, 450066 China; 13 Department of Anthropology & Ethnology, Xiamen University, Xiamen, 361005 China; 14 Liaoning Provincial Institute of Cultural Relics and Archaeology, Shenyang, 110003 China; 15 Shaanxi Academy of Archaeology, Xi’an, 710054 China; 16 Institute of Archaeology, Chinese Academy of Social Sciences, Beijing, 100710 China; 17 School of Biological Sciences, Seoul National University, Seoul, 08826 Republic of Korea; 18 Key Laboratory for Evolution of Past Life and Environment in Northeast Asia (Jilin University), Ministry of Education, Changchun, 130021 China

**Keywords:** Biological anthropology, Genomics, Population genetics, DNA sequencing

## Abstract

Northern China harbored the world’s earliest complex societies based on millet farming, in two major centers in the Yellow (YR) and West Liao (WLR) River basins. Until now, their genetic histories have remained largely unknown. Here we present 55 ancient genomes dating to 7500-1700 BP from the YR, WLR, and Amur River (AR) regions. Contrary to the genetic stability in the AR, the YR and WLR genetic profiles substantially changed over time. The YR populations show a monotonic increase over time in their genetic affinity with present-day southern Chinese and Southeast Asians. In the WLR, intensification of farming in the Late Neolithic is correlated with increased YR affinity while the inclusion of a pastoral economy in the Bronze Age was correlated with increased AR affinity. Our results suggest a link between changes in subsistence strategy and human migration, and fuel the debate about archaeolinguistic signatures of past human migration.

## Abstract (web summary)

Northern China contains some of the world’s earliest farming societies. Here, authors use 55 ancient genomes to trace the genetic history of human migrations across northern China for the last 7500 years, and document genetic changes mirroring shifts in subsistence strategy.

## Introduction

**Fig. 1:**
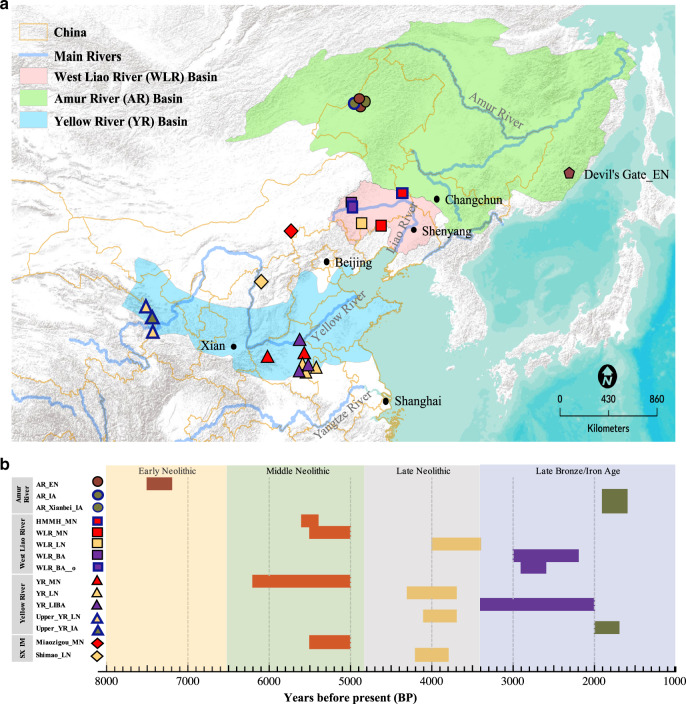
Geographic location and dates of ancient individuals. **a** Location of the 19 archeological sites covering 55 ancient individuals in this study. Each symbol corresponds to a site from a specific region: circle (AR); square (WLR); triangle (YR); diamond (sites from Inner Mongolia or Shaanxi) (see Table 1 for details). The published Early Neolithic genomes from the Russian Far East (“Devil’s Gate_EN”)^57,58^ are also indicated. The three major river basins in northern China are indicated in different color shades, namely Amur River Basin in light green, West Liao River Basin in pink, and Yellow River Basin in light blue. The base map was prepared from the ArcGIS “World Terrain Base” included in the ArcGIS desktop standard v. 9.2. ArcGIS user license was purchased by, and authorized to, the Max Planck Institute for the Science of Human History (MPI-SHH, Jena, Germany). **b** Calibrated radiocarbon dates and relative dating of ancient samples in this study. The archeological sites are ordered according to their locations. SX and IM refer to Shaanxi province and the Inner Mongolia Autonomous region of China, respectively. Their geographic locations are intermediate between the WLR and YR. Colors correspond to samples of different time periods: EN Early Neolithic, MN Middle Neolithic, LN Late Neolithic, BA Bronze Age, LBIA Late Bronze and Iron Age, IA Iron Age.

China is one of the earliest independent centers in the world for the domestication of cereal crops, second only to the Near East, with the rainfed rice agriculture in the Yangtze River Basin in southern China^1,2^, and dryland millet agriculture in northern China^2–6^. Northern China represents a large geographic region that encompasses the Central Plain in the middle-to-lower Yellow River (YR) basin, the birthplace of the well-known YR civilization since the Neolithic period. However, northern China extends far beyond the Central Plain and includes several other major river systems in distinct ecoregions (Fig. 1). Especially, it is now well received that the West Liao River (WLR) region in northeast China (Fig. 1) played a critical role distinct from the YR region in the adoption and spread of millet farming^3,6^. Both foxtail (*Setaria italica*) and broomcorn millets (*Panicum miliaceum*) were first cultivated in the WLR and lower reaches of the YR basins since at least 6000 BCE^3,6^. In the ensuing five millennia, millets domesticated in northern China spread across east Eurasia and beyond. Millets had served as one of the main staple foods in northeast Asia, particularly until the introduction of maize and sweet potato in the 16–17th centuries^2–7^.

Both the YR and the WLR are known for rich archeological cultures that relied substantially on millet farming^8,9^. By the Middle Neolithic (roughly 4000 BCE), complex societies with a substantial reliance on millet farming had developed in the WLR (Hongshan culture; 4500–3000 BCE)^10,11^ and in the YR (Yangshao culture; 5000–3000 BCE) basins^11^. For example, excavations of Hongshan societies in the WLR yielded public ceremonial platforms with substantial offerings including numerous jade ornaments, among which the “Goddess Temple” at the Niuheliang site is the most famous^10,12^. The establishment of the Middle Neolithic complex societies appears to have been associated with rapid population growth and cultural innovation, and may have been linked to the dispersal of two major language families, Sino-Tibetan from the YR^13,14^ and Transeurasian from the WLR^15^, although some scholars debate the genealogical unity of the latter^16,17^.

Compared with the YR region where crop cultivation already took the status of the dominant subsistence strategy by the Middle Neolithic, the level of reliance on crops in the WLR region has changed frequently in association with changes in climate and archeological culture. For example, paleobotanical and isotopic evidence suggest that the contribution of millets to the diet of the WLR people steadily increased from the Xinglongwa to Hongshan to Lower Xiajiadian (2200–1600 BCE) cultures^18^, but was partially replaced by nomadic pastoralism in the subsequent Upper Xiajiadian culture (1000–600 BCE). Although many archeologists associated this subsistence switch with a response to the climate change^19,20^, it remains to be investigated whether substantial human migrations mediated these changes. The WLR region adjoins the Amur River (AR) region to the northeast, in which people continued to rely on hunting, fishing, and animal husbandry combined with some cultivation of millet, barley, and legumes into the historic era^21,22^. Little is known to what extent contacts and interaction between YR and WLR societies affected the dispersal of millet farming over northern China and surrounding regions. More generally, given the limited availability of ancient human genomes so far, prehistoric human migrations and contacts as well as their impact on present-day populations are still poorly understood in this region.

Here, we present the genetic analysis of 55 ancient human genomes from various archeological sites representative of major archeological cultures across northern China since the Middle Neolithic. By the spatiotemporal comparison of their genetic profiles, we provide an overview of past human migration and admixture events in this region and compare them with changes in subsistence strategy.

## Results

### Ancient genome data production

We initially screened a total of 107 ancient individuals across northern China by shallow shotgun sequencing of one Illumina sequencing library per individual (Supplementary Data 1). These samples came from 19 archeological sites from the AR (three sites; 5525 BCE to 250 CE), WLR (four sites; 3694–350 BCE), and YR (ten sites; 3550–50 BCE) Basins, as well as sites from intermediate regions in Shaanxi province (one site; 2250–1950 BCE) and Inner Mongolia Autonomous region (one site; 3550–3050 BCE), spanning a geographic region of ~2300 km from north to south and covering six millennia (Fig. 1 and Table 1). We further sequenced 55 individuals with sufficient preservation of DNA to an autosomal coverage of ×0.03–7.53 (Table 1, Supplementary Table 1, and Supplementary Data 1).

**Table 1:** Summary of ancient samples reported in this study.

Region	Group label	Site names	Archeological culture	Date range^a^ (cal. BCE)
AR	AR_EN	Wuqi (1), Zhalainuoer (1)	–	5525–5320
	AR_IA	Zhalainuoer (1)	–	66–222 CE
	AR_Xianbei_IA	Mogushan (3)	Xianbei	50–250 CE
WLR	HMMH_MN	Haminmangha (1)	Haminmangha	3694–3636
	WLR_MN	Banlashan (3)	Hongshan	3550–3050
	WLR_LN	Erdaojingzi (3)	Lower Xiajiadian	2050–1344
	WLR_BA	Longtoushan (2)	Upper Xiajiadian	1050–350
	WLR_BA _o	Longtoushan (1)	Upper Xiajiadian	1050–350
Inner Mongolia	Miaozigou_MN	Miaozigou (3)	Miaozigou	3550–3050
Shaanxi	Shimao_LN	Shengedaliang (3)	Shimao	2250–1950
Upper YR	Upper_YR_LN	Jinchankou (1), Lajia (6)	Qijia	2050–1850
	Upper_YR_IA	Dacaozi (4)	–	50–150 CE
YR (central plain)	YR_MN	Xiaowu (1), Wanggou (7)	Yangshao	3550–3050
	YR_LN	Haojiatai (2), Pingliangtai (4), Wadian (2)	Longshan	2275–1844
	YR_LBIA	Luoheguxiang (2), Jiaozuoniecun (2), Haojiatai (2)	–	1550–50

*AR* Amur River Basin, *WLR* West Liao River Basin, *YR* Yellow River Basin.

^a^Combination of all calibrated ^14^C dates (2-sigma range) and estimates from archeological contexts across individuals available in each group. Individual dates are available in Supplementary Table 1.

**Fig. 2:**
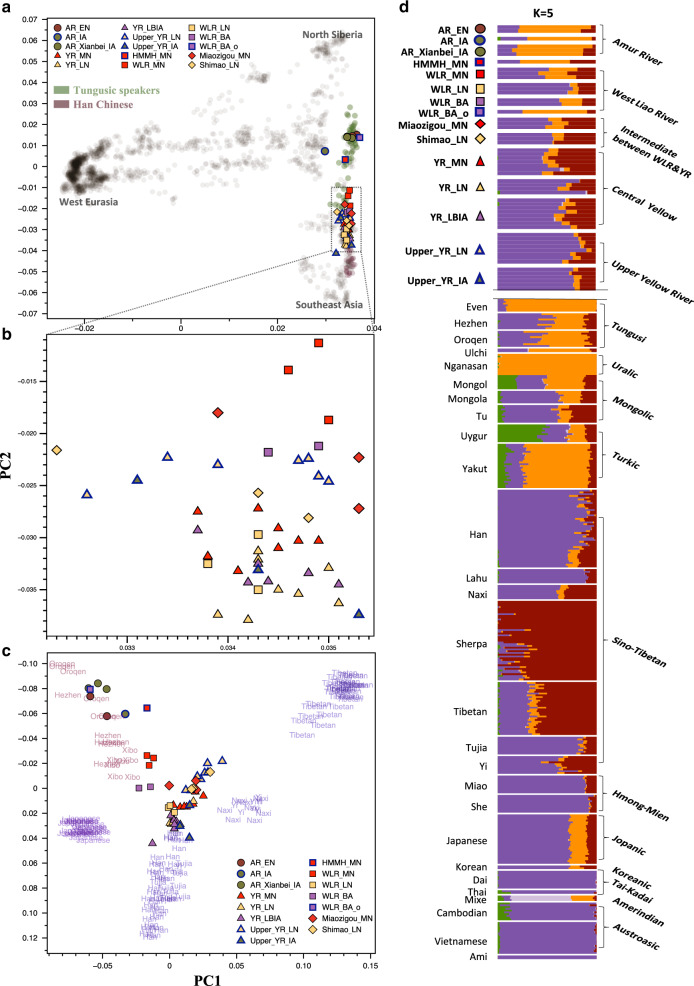
A summary of the genetic profiles of the ancient and present-day East Asian populations. **a** The first two principal components constructed from 2077 present-day Eurasians; the ancient individuals are projected onto the first two PCs. Color-filled shapes represent ancient individuals, with the color-shape combinations as used in Fig. 1. Opaque circles represent the present-day individuals used for calculating PCs. Tungusic-speaking populations and Han Chinese are marked by green and purple shades, respectively. Individuals from other populations are marked by gray shades. The population labels of present-day individuals are provided in Supplementary Fig. 4. **b** A zoom-in visualization of the WLR and YR clusters in **a**. **c** The first two principal components calculated from present-day individuals from nine East Asian populations in the “1240k-Illumina” dataset. Present-day individuals are marked by their corresponding population names. **d** ADMIXTURE results for the “1240k-Illumina” dataset at *K* = 5. Only the East Asian populations are plotted. Present-day populations are sorted and colored according to their linguistic families.

We verified the authenticity of the genome data by multiple measures. All samples showed postmortem chemical damage characteristic of ancient DNA (Supplementary Fig. 1 and Supplementary Table 2). They showed a low level of modern human contamination, <4% for mitochondrial estimates of all individuals and <5% for nuclear estimates of all males except for one with low coverage (6.3% contamination with ×0.07 coverage; Supplementary Tables 1 and 2). For each sample, we produced haploid genotypes by randomly sampling a single high-quality base for 593,124 autosomal single-nucleotide polymorphisms (SNPs) included in the Affymetrix “HumanOrigins” platform and 249,162 SNPs in the “1240k-Illumina” dataset, respectively. We then merged them with published ancient genomic data (Supplementary Data 2) and present-day individuals in the “HumanOrigins” or “1240k-Illumina” dataset^23,24^ (Supplementary Data 3) The ancient individuals from this study cover 11,690–586,085 SNPs in the “HumanOrigins” panel and 4481–244,000 SNPs on the “1240k-Illumina” panel^25–27^ (Supplementary Table 1). For group-based analyses, we primarily grouped ancient individuals based on their date, geographic region, and archeological context as well as their genetic profile (Fig. 2 and Table 1). We removed first-degree relatives in the group-based analyses to guarantee sample independence (Supplementary Note 2 and Supplementary Figs. 2 and 3).

### Genetic grouping of ancient individuals from northern China

Principal component analysis (PCA) of 2077 present-day Eurasian individuals in the “HumanOrigins” dataset^23,24^ (Supplementary Data 3) shows that the ancient individuals from northern China are separated into distinct groups (Fig. 2a, b and Supplementary Fig. 4). The ancient individuals fall within present-day eastern Eurasians along PC1. Likewise, they also harbor derived alleles characteristic of present-day East Asians and associated with potentially adaptive phenotypes^28–30^ (Supplementary Note 2 and Supplementary Table 11). However, they fall on different positions on PC2, which separates eastern Eurasians in a largely north-south manner (northern Siberian Nganasan at the top and Austronesian-speaking populations in Taiwan at the bottom). Ancient individuals from this study form three big clusters, with AR individuals to the top, YR individuals to the bottom, and WLR individuals in between, which largely reflected their geographic origin. To focus on variation within East Asians, we then used a panel of nine present-day East Asian populations in the “1240k-Illumina” dataset^25–27^ which includes highland Tibetans in large numbers. The first two PCs separate Tungusic-speakers (e.g. Oroqen, Hezhen, Xibo), Tibetans, and lowland East Asian populations (e.g. Han and Tujia) (Fig. 2c). Here fine-scaled clustering of ancient individuals, especially those from the YR and WLR, are more visible than in the Eurasian PCA. Unsupervised ADMIXTURE analysis shows a similar pattern (*K* = 5; Fig. 2d and Supplementary Figs. 5 and 6) that all ancient individuals harbor three ancestral components, and ancient individuals from the same river basins share similar genetic compositions, consistent with their PCA positions.

### Long-term genetic stability of AR populations

In both the Eurasian and East Asian PCA, two early Neolithic hunter-gatherers (“AR_EN”) and three Iron Age individuals (“AR_Xianbei_IA”; second century CE; Xianbei context) from the Upper AR, and one Bronze Age WLR individual from a nomadic pastoralist context (“WLR_BA_o”) form a tight cluster that falls within the range of present-day AR populations, who are mostly Tungusic speakers (Fig. 2 and Supplementary Fig. 4). One individual (AR_IA) falls outside of the AR cluster and slightly shifted in PCA along PC1 towards the Mongolic-speakers (Fig. 2a, b and Supplementary Fig. 4), but this is likely an artifact due to his low coverage (×0.068) and a small amount of contamination (6.3 ± 6.4%; point estimate ± 1 standard error measure, s.e.m.; Supplementary Tables 1 and 2). Ancient and present-day AR populations also show similar genetic profiles in ADMIXTURE^31^ analysis (Fig. 2d and Supplementary Fig. 6). Between pairs of AR populations, ancients as well as present-day samples from the lower AR, we observe large outgroup-*f*_*3*_ statistics supporting their close genetic affinity (Supplementary Figs. 7 and 8). Furthermore, we formally confirm that they are largely cladal to each other. First, the nonsignificant statistic *f*_*4*_(AR_1_, AR_2_; X, Mbuti) statistics are nonsignificant (*Z* < 3) for most outgroup populations (X’s) except for the two present-day Siberian populations (Nganasan and Itelmen; Supplementary Figs. 9 and 10) who may have experienced historical genetic exchanges with the AR-related gene pools. Second, the qpWave analysis cannot tell pairs of AR populations apart in terms of their affinity to the outgroups (*p* > 0.05; Supplementary Table 3A).

Although the AR populations do not show a substantial change over time regarding their affinity to populations outside the AR, a published test of the genetic continuity in the strictest sense^32^ rejects the hypothesis that the ancient AR populations in this study are the direct ancestor of the present-day ones (Supplementary Table 3B). This suggests a stratification within the AR gene pool and presumably gene flows between the AR populations during the formation of the present-day populations.

### Temporal changes in the YR genetic profile

Ancient YR individuals from the Central Plain area form a cluster distinct from the AR individuals in the PCA and likewise share a similar genetic profile in the ADMIXTURE analysis (Fig. 2 and Supplementary Fig. 4). However, we also observe small but significant differences between them: Late Neolithic Longshan individuals (“YR_LN”) are genetically closer to present-day populations from southern China and Southeast Asia (“SC–SEA”) than earlier Middle Neolithic Yangshao ones (“YR_MN”), measured by positive *f*_*4*_(YR_LN, YR_MN; X, Mbuti) (+3.7 s.e.m. with X = Ami; Supplementary Fig. 11). This provides a genetic parallel to our observation of a significant increase of rice farming in middle and lower YR between Middle Neolithic Yangshao and Late Neolithic Longshan periods (Supplementary Note 2 and Supplementary Fig. 12). We detect no further change in later Bronze/Iron Age individuals (“YR_LBIA”), shown by nonsignificant *f*_*4*_(YR_LN, YR_LBIA; X, Mbuti) (|*Z*| < 3; Supplementary Fig. 13). Unlike the AR region, we do not find present-day populations that form a clade with YR_LBIA (Supplementary Fig. 14). Han Chinese, a dominant ethnic group currently residing in the Central Plain, clearly show extra affinity with SC–SEA populations (max |*Z*| = 10.3 s.e.m.). Tibeto-Burman-speaking Naxi from southwest China show much reduced but still significant differences from ancient YR populations (max |*Z*| = 4.0 s.e.m.; Supplementary Fig. 15). These results suggest a long-term genetic connection between YR populations across time but with an important axis of exogenous genetic contribution that may be related to the northward expansion of rice farming by population migrations from south China (e.g. Yangtze river).

Neolithic genomes from the region surrounding the Central Plain show that the YR genetic profile had a wide geographic distribution. Genomes from Middle Neolithic Inner Mongolia (“Miaozigou_MN”) and Late Neolithic Shanxi province (“Shimao_LN”), both located between the YR and WLR, are genetically similar to each other and to ancient YR populations (Fig. 2 and Supplementary Figs. 16 and 17). Late Neolithic individuals from the upper YR (“Upper_YR_LN”), who are associated with the Qijia culture, also show a similar pattern (Supplementary Figs. 16 and 17). We model these groups as a mixture of YR farmers and AR hunter-gatherers, with a majority ancestry (~80%) coming from the YR (*p* ≥ 0.278; Supplementary Table 4). Iron Age genomes from the upper YR region (“Upper_YR_IA”) show an even higher YR contribution, compatible with 100% YR ancestry (94.7 ± 5.3%; Supplementary Table 4).

Archeological studies suggest a pivotal role of the mid-altitude region at the northeastern fringe of the Tibetan plateau, where the Qijia culture was located, in the permanent human occupation of the plateau after around 1600 BCE^33^. More broadly, recent linguistic studies favor a northern origin of Sino-Tibetan languages, suggesting the Yangshao culture as their likely origin^13,14^. We explored genetic connections between present-day Sino-Tibetan and ancient YR populations using admixture modeling. Tibetans are modeled as a mixture of Sherpa and Upper_YR_LN, although other sources also work (Supplementary Table 5). This provides a likely local source for the admixture signals previously reported^25,34^. Among the other Sino-Tibetan-speaking populations in our data set, Naxi and Yi are indistinguishable from YR_MN to our resolution, while Lahu, Tujia, and Han show a prevailing influence from a gene pool related to the SC–SEA populations (Supplementary Table 6). Our results are compatible with the above-mentioned linguistic and archeological scenarios, although we find other models also marginally work due to resolution of our genetic data (Supplementary Tables 5 and 6).

### Correlated changes of genes and subsistence in WLR

**Fig. 3:**
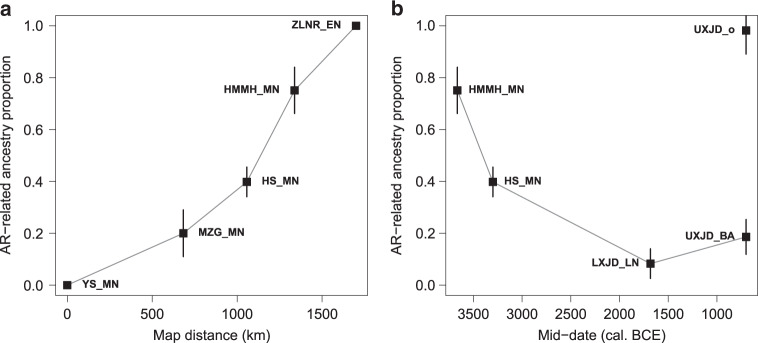
qpAdm modeling of the ancient populations in Northern China. Modeling ancient populations as a mixture of YR_MN and AR_EN. **a** Middle Neolithic populations in this study. *X*-axis shows the great circle distance from the YR_MN sites and *y*-axis shows estimates of AR-related ancestry proportion (represented by AR_EN). **b** WLR populations from Middle Neolithic to Bronze Age. Black squares represent the point estimates. Vertical bars represent ± 1 s.e.m. range, estimated by 5 cM block jackknifing.

The WLR region, located between the YR and AR, shows frequent genetic changes over time. Middle Neolithic WLR individuals fall between the AR and YR clusters in the PCA: three belonging to the Hongshan culture (“WLR_MN”) are closer to the YR cluster while one from a nearby site (“HMMH_MN”) falls closer to the AR cluster (Fig. 2a–c). *F*_*4*_ statistics confirm that both groups are intermediate between AR and YR groups, represented by AR_EN and YR_MN, respectively (Supplementary Fig. 18). We adequately model both groups as a mixture of AR and YR groups, with higher AR contribution to HMMH_MN (39.8 ± 5.7% and 75.1 ± 8.9% for WLR_MN and HMMH_MN, respectively; Supplementary Table 4). Taking contemporaneous Miaozigou_MN from Inner Mongolia into account, we observe a sharp transition from a predominantly YR-related to an AR-related genetic profile within ~600 km distance during the Middle Neolithic (Fig. 3a). Linguistically, the WLR Basin has been associated with the origin of the Transeurasian language family and the mixture between AR and YR groups may find a correlate in the borrowing between Transeurasian linguistic subgroups and Sinitic ones, becoming more intensive from the Bronze Age onwards^35^.

In addition to this genetic heterogeneity around the Middle Neolithic WLR, a temporal comparison within the WLR also shows an interesting pattern of genetic changes. First, Late Neolithic genomes associated with the Lower Xiajiadian culture (“WLR_LN”) overlap with the ancient YR cluster in the PCA and show less affinity to Siberian populations compared with WLR_MN (Fig. 2 and Supplementary Fig. 19). QpAdm modeling estimates a major YR contribution: 88 or 74% when AR_EN or WLR_MN is used as a secondary source, suggesting a substantial northward influx from a YR-related population between the Middle and Late Neolithic (Fig. 3b and Supplementary Table 4). Interestingly, the Bronze Age WLR individuals, associated with the Upper Xiajiadian culture (“WLR_BA”), again show a genetic change but to an opposite direction from the Middle-to-Late Neolithic, with one individual (“WLR_BA_o”) being indistinguishable from the ancient AR individuals (Supplementary Figs. 9 and 10). Compared with AR_EN, he has extra affinity with later AR individuals (“AR_Xianbei_IA”) and multiple present-day Tungusic-speaking populations (Supplementary Fig. 20). We speculate that this individual may signify a recent migration from an AR-related gene pool into the WLR. Indeed, the remaining two individuals (“WLR_BA”) are modeled as a mixture of WLR_LN and WLR_BA_o with 21 ± 7% contribution from the latter (Fig. 3b and Supplementary Table 7). A previous archeological study suggests that the Lower to Upper Xiajiadian transition was associated with a climatic change to a drier environment less favorable to millet farming and led to southward population migrations within the WLR region^20^. Our results highlight the other side of the process: climate change made a pastoral economy more favorable and may have led to an influx of people already practicing it.

## Discussion

In this study we present a large-scale survey of ancient genomes from northern China that covers many, although not all, major archeological cultures in the region. Especially, our study provides the first genomic look into people who lived in the earliest complex societies of northern China, i.e., Yangshao and Hongshan cultures in the YR and WLR, respectively. By providing genomic time series in these regions, we could detect genetic changes in each region over time and associate them with external genetic sources and with sociocultural and environmental changes.

In contrast to the long-term stability of the genetic profile of the AR populations who practiced limited food production, we observe frequent genetic changes in the two centers of complex millet-farming societies in northern China, the YR and WLR, over the last six millennia. The WLR genetic profile changes over time in close association with changes in subsistence strategy. More specifically, an increase in the reliance on millet farming between the Middle-to-Late Neolithic is associated with higher YR genetic affinity in the Late Neolithic WLR, while a partial switch to pastoralism in the Bronze Age Upper Xiajiadian culture is associated with lower YR affinity. In the Middle Neolithic, we observe a sharp transition from YR- to AR-related genetic profiles around the WLR. Such a spatial genetic heterogeneity may have persisted in the WLR during the Bronze and Iron Ages although our current data are not sufficient to test such a hypothesis. The Middle-to-Late Neolithic genetic change in the YR also coincides with the intensification of rice farming in the Central Plain, which may provide another case of change in subsistence strategy via demic diffusion. We acknowledge that our current data set lacks ancient genomes from candidate source populations which may have brought rice farming into the Central Plain and call for archaeogenetic studies for them, especially Neolithic people from the Shandong and Lower Yangtze River regions. Future studies of ancient genomes across China, particularly the genomes of the first farmers will be critical to test the representativeness of the genomes reported in this study, to understand the genetic changes we detected at finer genetic, archeological and geographic scales, and to test the evolutionary correlation between archeological cultures, languages, and genes.

## Methods

### Laboratory procedures

We initially screened 107 ancient samples (Supplementary Data 1) in dedicated clean facilities at the ancient DNA lab of Jilin University, China, following published protocols for DNA extraction and library preparation^36,37^. Prior to sampling, we wiped all skeletal elements with 5% bleach and irradiated with UV-light for 30 min from each side. We drilled teeth to obtain fine powder using a dental drill (Dremel, USA). We sampled the dense part of petrous bones around the cochlea by first removing the outer part using the sandblaster (Renfert, Germany), and then grinding the clean inner part into fine powder with the mixer mill (Retsch, Germany). We digested the powder (50–100 mg) in 900 μl 0.5 M EDTA (Sigma-Aldrich), 16.7 μl of Proteinase K (Sigma-Aldrich), and 83.3 μl ddH_2_O (Thermo Fisher, USA) at 37 °C for 18 h. Then we transferred the supernatant to a MinElute silica spin column (QIAGEN, Germany) after fully mixed with the 13 ml custom binding buffer [5 M guanidine hydrochloride (MW 95.53), 40% Isopropanol, 90 mM Sodium Acetate (3 M), and 0.05% Tween-20] followed by two washes with PE buffer (80% ethanol). Then we eluted the DNA with 100 μl TET buffer (QIAGEN, Germany).

We prepared one double-stranded dual-indexed library from a 20 μl aliquot of each extract. We performed blunt-end-repair of DNA fragments by adding T4 Polynucleotide Kinase (0.5 U/μl; Thermo Fisher) and T4 DNA Polymerase (0.08 U; Thermo Fisher), and incubating at 25 °C for 15 min. We retrieved the repaired DNA fragments using a standard MinElute purification step (Qiagen; Germany), and then by eluting the samples in 18 μl TET (Qiagen, Germany). We ligated Illumina adapters (0.25 μM adapter mix) onto the blunt-ends using 1X Quick Ligase (New England Biolabs, NEB) in a total reaction volume of 40 μl, followed by another MinElute purification step. We performed the final fill-in step by adding 1X isothermal buffer, 0.4 U/μl Bst-polymerase (NEB) and 250 μM dNTP Mix (Thermo Fisher), followed by incubating at 37 °C for 30 min and then at 80 °C for 20 min. We then indexed the libraries with uniquely combined double indices. We purified indexed products using AMPure XP bead (Beckman Coulter Ltd). We qualified the clean-up libraries by Qubit 2.0 (Thermo Fisher). We then sequenced the libraries on an Illumina HiSeq X10 instrument at the Annoroad Company, China in the 150-bp paired-end sequencing design.

### Sequence data processing

Sequence reads were demultiplexed by allowing one mismatch in each of the two 8-bp index sequences. We clipped the Illumina sequencing adapters by AdapterRemoval v2.2.0^38^. We mapped merged reads to the human reference genome (hs37d5; GRCh37 with decoy sequences) using BWA v0.7.12^39^. We removed PCR duplicates using DeDup v0.12.2^40^. To minimize the impact of postmortem DNA damage on genotyping, we prepared additional “trimmed” BAM files by soft masking up to 10 bp on both ends of each read using the trimbam function on bamUtils v1.0.13^41^ based on the DNA misincorporation pattern of each library. For the SNPs in the “1240k” panel^42,43^, we randomly sampled a single high-quality base (Phred-scaled base quality score 30 or higher) as pseudodiploid genotypes using the pileupCaller program (https://github.com/stschiff/sequenceTools). For C/T and G/A SNPs, we used trimmed BAM files. For the remaining SNPs, we used untrimmed BAM files.

### Reference datasets

We compared our ancient individuals to two sets of world-wide genotype panels, one based on the Affymetrix HumanOrigins Axiom Genome-wide Human Origins 1 array (“HumanOrigins”; 593,124 autosomal SNPs)^23^ and the other intersecting over multiple Illumina array platforms (‘1240k-Illumina’; 249,162 autosomal SNPs)^25–27^. The latter include more Tibetan/Sherpa genomes (30 Tibetans and 45 Sherpa) for an in-depth analysis of Sino-Tibetan-speaking populations. We augmented both data sets by adding the Simons Genome Diversity Panel^44^ and published ancient genomes (Supplementary Data 2).

### Ancient DNA authentication

We used multiple methods to assess the quality of the ancient genomes from northern China. First, we tabulated patterns of postmortem chemical modifications expected for ancient DNA using mapDamage v2.0.6^45^. Second, we estimated mitochondrial contamination rates for all individuals using Schmutzi v1.5.1^46^. Third, we measured the nuclear genome contamination rate in males based on X chromosome data as implemented in ANGSD v0.910^47^. Since males have only a single copy of the X chromosome, mismatches between bases, aligned to the same polymorphic position, beyond the level of sequencing error are considered as evidence of contamination.

### Genetic sexing and uniparental haplogroup assignment

We assigned the molecular sex of our ancient samples by comparing the ratio of X and Y chromosome coverages with autosomes^48^. We generated the mtDNA consensus sequences of our ancient individuals using the Geneious v11.1.3 software^49^, and then determined their mtDNA haplogroups using HaploGrep2^50^. We determined the male Y chromosome haplogroup by examining a set of positions on the 25,660 diagnostic positions on the ISOGG database, and assigned the final haplogroups by the most downstream derived SNPs (Supplementary Table 2).

### Population structure analysis

We performed PCA as implemented in the smartpca v16000^51^ using a set of 2077 present-day Eurasian individuals from the “HumanOrigins” dataset and a subset of 266 East Asian individuals using the “1240k-Illumina” dataset with the option “lsqproject: YES” and “shrinkmode: YES.” We also performed unsupervised admixture analysis with ADMIXTURE v1.3.0^31^. We removed genetic markers with minor allele frequency smaller than 1% and pruned for linkage disequilibrium using the *“--indep-pairwise 200 25 0.2*” option in PLINK v1.90^52^.

### Genetic relatedness analysis

We used pairwise mismatch rate (pmr)^53^ and lcMLkin v0.5.0^54^, to determine the genetic relatedness between ancient individuals. We calculated pmr for all pairs of ancient northern China individuals using the autosomal SNPs of the 1240k panel^42^ and kept individual pairs with at least 8000 SNPs covered by both to minimize an artificial bias between poor-quality samples. We then used lcMLkin to estimate more details of relatedness.

### F-statistics

We used outgroup-*f*_*3*_ statistics^55,56^ to obtain a measurement of genetic relationship between two populations since their divergence from an African outgroup. We calculated *f*_*4*_ statistics with the “*f4mode: YES*” function in the admixtools^56^. *F*_*3*_ and *f*_*4*_ statistics were calculated using qp3Pop v435 and qpDstat v755 in the admixtools package.

### Admixture modeling with qpAdm

We modeled our ancient northern China populations using the qpWave/qpAdm framework (qpWave v410 and qpAdm v810)^43^. We used the following nine populations in both “HumanOrigins” and “1240k-Illumina” datasets as outgroup (“OG1”): Mbuti, Natufian, Onge (Onge.DG in “1240k-Illumina” panel), Iran_N, Villabruna, Mixe (Mixe.DG in “1240k-Illumina” panel), Ami (Ami.DG in “1240k-Illumina” panel), Nganasan and Itelmen (Itelmen.DG in “1240k-Illumina” panel). This set includes an African outgroup (Mbuti), early Holocene Levantine hunter-gatherers (Natufian), Andamanese islanders (Onge), early Neolithic Iranians from the Tepe Ganj Dareh site (Iran_N), late Pleistocene European hunter-gatherers (Villabruna), Central Native Americans (Mixe), an indigenous group native to Taiwan (Ami), indigenous Samoyedic people inhabiting the Taymyr Peninsula in north Siberia (Nganasan), and an ethnic group native to the Kamchatka Peninsula in Russia (Itelmen). Given that some AR populations show increased genetic affinity with Nganasan and Itelmen (Supplementary Figs. 9 and 10), we further modeled the AR and nearby populations using the outgroups with exclusion of Nganasan and Itelmen from OG1.

### The genetic continuity test

For pairs of ancient and present-day AR populations, we tested if the ancient individuals can be placed into the direct ancestral population of the present-day ones using a published method^32^, downloaded from https://github.com/Schraiber/continuity. Specifically, we prepared read count data of ancient individuals from the pileup tables used for haploid genotyping, and allele count data of five present-day AR populations (Evenk_FarEast, Nanai, Negidal, Nivh, Ulchi) from the eigenstrat format genotype data. Starting from 593,124 autosomal SNPs in the HumanOrigins panel, we excluded SNPs fixed in each present-day population and applied a default coverage filter for ancient individuals (min_cutoff = 2.5, max_cutoff = 97.5; removing 5% or less sites with most extreme coverage). For the remaining SNPs used for the analysis, we fitted beta prior for the allele count distribution of present-day populations to account for the relatively small sample size of the present-day populations. Then we calculated likelihoods of the models that assumes the continuity (*t*_*2*_ = 0; no genetic drift private to the ancient individuals; the null hypothesis) and that does not (*t*_*2*_ > 0; an alternative hypothesis). Likelihood ratio test provides *p* value for testing genetic continuity.

### Reporting summary

Further information on research design is available in the Nature Research Reporting Summary linked to this article.

## Supplementary information

Supplementary Information

Peer Review File

Reporting Summary

Description of Additional Supplementary Files

Supplementary Data 1

Supplementary Data 2

Supplementary Data 3

## Data Availability

Raw FastQ and alignment files (BAM format) are available at the European Nucleotide Archive under the accession number PRJEB36297. Haploid genotype data of ancient individuals in this study on the 1240k panel are available in the EIGENSTRAT format from the following link: https://edmond.mpdl.mpg.de/imeji/collection/5oV1TtHlsYggGBT3.

## Code availability

All analyses performed in this study are based on publicly available programs. Program names, versions, and nondefault options are described in the “Methods” section.
