# Supplementary material for: Ancient genomes from northern China suggest links between subsistence changes and human migration
Source: Nat Commun. 2020 Jun 1;11:2700. doi: 10.1038/s41467-020-16557-2 (PMC7264253; doi:10.1038/s41467-020-16557-2)
Supplement: Supplementary file 1 — Supplementary Information [file 41467_2020_16557_MOESM1_ESM.pdf]

Supplementary Information

Ancient genomes from northern China suggest links between subsistence changes and human migration

Chao Ning, Tianjiao Li, Ke Wang, Fan Zhang, Tao Li, Xiyan Wu, Shizhu Gao, Quanchao Zhang, Hai Zhang, Mark J. Hudson, Guanghui Dong, Sihao Wu, Yanming Fang, Chen Liu, Chunyan Feng, Wei Li, Tao Han, Ruo Li, Jian Wei, Yonggang Zhu, Yawei Zhou, Chuan-Chao Wang, Shengying Fan, Zenglong Xiong, Zhouyong Sun, Maolin Ye, Lei Sun, Xiaohong Wu, Fawei Liang, Yanpeng Cao, Xingtao Wei, Hong Zhu, Hui Zhou, Johannes Krause*, Martine Robbeets*, Choongwon Jeong*, Yinqiu Cui*

* Correspondence to: cuiyq@jlu.edu.cn (Y.C), cwjeong@snu.ac.kr (C.J.), robbeets@shh.mpg.de (M.R.), krause@shh.mpg.de (J.K.)

This file includes:

Supplementary Notes 1 and 2
Supplementary Figures 1 to 21
Supplementary Tables 1 to 11
Supplementary References

Supplementary Note 1. Archaeological Background of the studied ancient individuals

China is one of the few independent centers for the world's earliest cereal domestication: rainfed rice agriculture in the Yangtze River basin in southern China, and dryland millet agriculture in the Yellow River (YR) and West Liao River (WLR) basins in northern China. By roughly 8,000 BP, the dryland farming was dominated by foxtail millet (*Setaria italica*) and broomcorn millet (*Panicum miliaceum*); these species are believed to have been domesticated in northern China[1,2]. From approximately 6,500 BP onward, the Hongshan culture in the WLR and the Yangshao culture in the YR became the dominant cultures in northern China, leaving a significant influence on the later cultures in the regions[3]. In contrast to the WLR and YR, indigenous peoples of the Amur River (AR) basin have relied on a mixed subsistence strategy of cereal crop farming augmented by hunting, gathering and fishing as well as animal husbandry. In the following sections, we provide a brief introduction on the natural settings and archaeological backgrounds of the three river regions in northern China.

1.1. The Amur River Valley

The Amur river (Heilongjiang "Black Dragon river" in Chinese) is the tenth longest river in the world, running approximately 4,000 km long, and forming the border between the Russian Far East and northeast China. The river arises from the Mongolian Plateau and flows eastward into the Sea of Okhotsk. The Amur River basin forms a mountainous region, in which mountain foothills and plateaus cover around two-thirds of the region. The region also includes a large area of plains that are located between the Zeya and Bureya rivers in the lower Amur valley. For example, the Sanjiang Plain is located at the confluence of the Amur with the Songhua and Ussuri Rivers and the Song-Nen plain at the confluence of the second Songhua and Nen Rivers. Because of the great diversity of landscapes such as floodplain wetlands, alpine tundra, steppe and forest, the basin is rich in biological diversity including the rare cranes, tigers, leopards and endemic fishes. As a result, people in this region have lived primarily on hunting and fishing as well as nomadic pastoralism, giving rise to several nomadic groups such as the Xianbei and Khitan[4,5].

The AR is known as one of the areas with the world's earliest pottery (~16,500 cal BC). The term "Neolithic" in the AR, as elsewhere in East Asia, is usually used to denote the period after the emergence of pottery[6]. However, unlike the Neolithization process in other regions, the earliest ceramics in the AR were found among the groups equipped with a microblade complex that belongs to the Upper Paleolithic culture with no obvious sign of a cultural transition. In this sense, some archaeologists have proposed that the AR should be taken as a representative example where the concept of the Neolithic Revolution marked by the arrival of multiple changes at once did not apply[7].

The main cultures in the AR, especially those from the Primorye region of the Russian Far East include the Paleolithic cultures such as Osipovka and the Gromatukha which were dated to roughly 13,000 BP[8]. This was then followed by the Neolithic Rudnaya (7,740-5,850 BP), Boisman (6400-5000 BP) and Zaisanovka (5800-3300 BP) cultures and by the Kirovska and other cultures of the Palaeometal Age (3000 - 1600 BP).

1.1.1. Archaeological context of the AR sites in this study

Wuqi farm site

The Wuqi farm site is located in Zhalainuoer district of Inner Mongolia, China. The site was

excavated by a joint team from the National Museum of Hulunbuir and the Manzhouli Municipal Institute of Cultural Relics in 2011. A total of four burials were excavated in this site of which two individuals were initially screened and only one (WQM4) was analyzed in this study due to the poor preservation of the other individual (WQM3):

- WQM3: 3590-4330 calBCE (3285±30 BP)
- WQM4: 5430-5270 calBCE (6445±40 BP)

Zhalainuoer coal mining site
The Zhalainuoer mining site is located in the Zhalainuoer area of Hulunbuir, Inner Mongolia. It is a surface pit about 4 km long and 1.2 km wide. Since 1933, when the first human cranium was discovered in the sediments, more than 16 human skulls have been found in this site. All the excavation materials were stored in the Zhalainuoer museum. Two individuals from this site produced genomic data:

- ZLNR-1: 66-222 calCE (1880±30 BP)
- ZLNR-2: 5525-5460 calBCE (7500±30 BP)

Mogushan site
The Mogushan Xianbei site is located in Hulunbuir, Inner Mongolia and is about 4 km north of Zhalainuoer city. Rescue excavations were conducted in 2011 by a joint team from Manzhouli Municipal Institute of Cultural Relics, the National Museum of Hulunbuir and Institute of Cultural Relics of Zhalainuoer district. The cemetery is about 600 meters long from south to the north and 50 meters wide. A total of 7 burials were excavated and marked from 2011MZMM2 to 2011MZMM7. Except for 2011MZMM2, 2011MZMM4 and 2011MZMM7, the remaining four burials were sacrificed with horses, cattle and/or goats. 2011MZMM7 is the only burial with two individuals buried together. Based on the stratigraphy, the cemetery was dated to the Eastern Han Dynasty. More than 90 funerary objects were excavated including pottery, stone tools, beads, gold, etc. Three individuals from the Mogushan site produced genomic data:

- MGS-M6: 50-250 CE
- MGS-M7L: Male, adult. 50-250 CE
- MGS-M7R: Female, adult. 50-250 CE

1.2. The West Liao River Valley
The West Liao River valley refers to the area with the western tributaries of the upper Liao River, located primarily in Inner Mongolia. The valley is situated in a transitional zone between the Inner Mongolian Plateau and the Manchurian Plain. The Khingan mountains set the boundary between the two zones, with the Great Khingan Range to the north of the valley and the Yanshan mountains to its south. The hydraulic system is especially developed in the upper part of the valley, where the West Liao, Laoha, Jiaolai, Xinkai, Daling, as well as other smaller rivers are distributed. Terraces on the banks of these rivers often form ideal locations for settlements and upland dry farming[9,10]. The geography of the West Liao River valley allows multiple subsistence strategies (hunting, fishing, gathering, and farming) and thus the coexistence of multiple ethnic groups and diverse cultures[11].

To the east of the West Liao River Valley lies the Manchurian (Northeastern) plain, a fertile agricultural plain drained by the Liao river proper and a natural corridor of communication to the north where the Songhua (Sungari) river offers access to the Amur. In ancient times, this Manchurian plain corridor was extremely important for trading and other contacts in northeast

Asia. Today, the Manchurian plain remains an important center of millet agriculture; for example, in 2017, northeast China (Liaoning, Jilin, Heilongjiang, and eastern Inner Mongolia) produced 27% of China's foxtail millet output, second only to northern China (35%)[12]. Paleoenvironmental reconstructions have suggested that the Chifeng region, located in the upper reaches of the West Liao River valley, was warm and humid from around 8,000 to 6,000 BP, with the landscape covered by deciduous and coniferous forests consisting mainly of Manchurian walnut (*Juglans mandshurica*), Chinese ash (*Fraxinus chinensis*), and pine trees. The average annual temperature is estimated at 6.5 to 7.5 ℃, about 3 ℃ higher than present-day for most parts of northeast China. It is further estimated that between 8,000-6,000 BP, the average temperature in January, the coldest month of the year, was -11 to -12 ℃; in contrast, the average temperature in July, the hottest month of the year, was 23 to 24 ℃. The annual precipitation was 400 to 500 mm[11]. Archaeological evidence suggests that broomcorn millets was first domesticated and cultivated in this region some 8,000 years ago[13,14].

1.2.1. The culture sequence of the West Liao River region

A series of archaeological cultures have been found in the West Liao River Valley between 9,000 and 2,500 BP. Major Neolithic cultures include the Xiaohexi (9,000-8,500 BP), Xinglongwa (8,200-7,400 BP), Zhaobaogou (7,500-6,500 BP), Fuhe (7,200-7,000 BP), Hongshan (6,500-5,000 BP), and Xiaoheyan (5,000-4,000 BP). Two successive Late Neolithic and Bronze Age cultures in the region are the Lower Xiajiadian (4,000-3,200 BP) and the Upper Xiajiadian (3,200-2,600 BP)[15].

The cultures above have their material remains densely distributed within the West Liao River valley, which is well known for old remains of domesticated millets. Many scholars agree that the West Liao River valley is a center for the origin of millet agriculture in northeast Asia[13-16].

Xiaohexi culture

Our knowledge about the Xiaohexi culture is limited to only a few excavated sites without associated radiocarbon dates. A total of 44 Xiaohexi culture sites have so far been recorded by national and regional surveys. Stratigraphy and cross-dating of cultural remains, however, suggest the Xiaohexi has an earlier start date than the Xinglongwa, therefore making Xiaohexi the earliest Neolithic culture in the West Liao River valley[10]. Opinions vary among scholars regarding whether the Xiaohexi people were the ancestors of the Xinglongwa people, but archaeologists generally agree on a close relationship between them based on the similarities in house plans, construction techniques, and some artefactual assemblages[10].

At the Xiaohexi site, three semi-subterranean houses, each having a hearth in the central part, were excavated. Unearthed stone tools include hoes, grinding stones, and pestles. Some scholars interpret these tools as an indication of agricultural activities. Flotation and identification of archaeobotanical remains at Xiaohexi suggest a predominance of fruits and nuts, with rare occurrence of cultivated plants[12]. However, recent isotope analyses of human bones report that C_4 plants, most likely millets, comprised up to 30% of the Xiaohexi people's diet. Consumption of wild C_4 grasses alone is unlikely to have resulted in such a high dietary proportion[12]. Thus, it is likely that millet cultivation started in the Xiaohexi period.

Xinglongwa culture

There are 110 sites of the Xinglongwa culture within the Upper West Liao River valley[10]. A few Xinglongwa sites yield some of the earliest domesticated millets in northeast Asia. For example,

at Xinglonggou, a Middle Xinglongwa village, archaeobotanists collected more than 1,400 carbonized broomcorn millet seeds and 60 foxtail millet seeds, all with possible characteristics of domestication[14]. Some millet grains were radiocarbon dated to 7,600 to 7,800 years old[13,14]. The excavators also found hoes—outnumbering other tool categories and presumably used for plowing in agricultural production—at Xinglonggou. Grinding stones unearthed at Xinglonggou retain starch grains sourced from both wild plants and domesticated millets[16]. Furthermore, isotope analyses suggest that millets contributed up to 60–70% of the Xinglonggou people's diet[17]. Other sites with the Xinglongwa cultural remains, such as Baiyinchanghan (7,600-7,400 BP) and Chahai (7,500-7,200 BP), also have starch grain evidence on grinding tools, suggesting on-site millet consumption[18,19]. In short, there is clear evidence for the cultivation of millets in the Xinglongwa period and millets - even if they were not the only cereal plants used—contributed much more to the human diet than before[14].

Zhaobaogou and Fuhe cultures
A total of 98 and 38 sites respectively belong to the Zhaobaogou and Fuhe cultures within the Upper West Liao River valley[10]. The Zhaobaogou and Xinglongwa cultures are similar especially in their spatial extent of cultural materials, settlement patterns, and artefactual assemblages (i.e., Z-motif and geometric patterns on pottery, stone hoes, and jade *jue* rings)[20]. Therefore, many archaeologists assume that the Zhaobaogou culture succeeded the Xinglongwa culture. There has been no flotation or archaeobotanical studies at sites of the Zhaobaogou culture, and thus no millet grains are known[21]. Nor are there published microbotanical (phytoliths and starch grains) or isotope analyses. A large number of stone tools are the only clue to understanding of the Zhaobaogou people's subsistence strategies. At Zhaobaogou, Xiaoshan, Nantaidi, Shuiquan and Baiyinchanghan, the excavators found many hoes, axes, grinding stones, and pestles, all presumably related to food production[21]. Despite the absence of harvesting tools, many scholars still believe that millet agriculture was practiced in the Zhaobaogou period[22]. The same can be said of the Fuhe culture.

Hongshan culture
Beginning around 6,500 BP, Hongshan communities emerged in the West Liao River valley, and archaeologists suggest that they later developed into the first chiefly societies in northeast China. The influence of the Hongshan culture crossed the boundaries of the West Liao River valley and reached, for example, present-day Jilin and Heilongjiang provinces, as shown by the distribution of painted ceramics and finely-made jades. The number of sites increased to 967 in the Upper West Liao River valley during this period. Large settlements (centers) were formed, surrounded by many smaller settlements[20,23]. Moreover, there is a diversity of agricultural tools for plowing, harvesting, and food processing[24]. Macro- and micro-botanical remains suggest millet farming of both broomcorn and foxtail at many Hongshan sites[10]. At Weijiawopu, an Early to Middle Hongshan site, archaeobotanists collected 33 foxtail and 13 broomcorn millet seeds[25]. At Haminmangha, a site dated to the Late Hongshan (5,500-5,000 BP), but characterized as a different culture called the Haminmangha, archaeobotanists collected 20 foxtail and 615 broomcorn carbonized millet grains. Isotope analyses reveal that millets contributed up to 70% of the human diet in the Early Hongshan and up to 80% in the Late Hongshan[17]. Overall, in Hongshan times millet agriculture was well established and widely practiced at or around occupation sites, sustaining more humans and animals (pigs).

Xiaoheyan culture
The number of sites decreased to 82 in the Xiaoheyan period[10], and cultural materials correspondingly became rarer. So far, there are no millet remains reported for sites of the Xiaoheyan culture[10]. However, isotopic reconstructions[26] on human bones unearthed at Jiangjialiang, an Early Xiaoheyan site in Yangyuan County of Hebei Province, show that the early Xiaoheyan people -although living outside of the West Liao River valley -consumed high proportions of C_4 plants (likely millets). Their carbon isotopic values are close to those of populations who consumed millets as the main plant food in their diet[16]. In other words, the practice of millet farming is likely to have continued, and the consumption of millets was high, in the Xiaoheyan period.

Lower and Upper Xiajiadian cultures
The late Neolithic and Bronze Age cultures-the Lower Xiajiadian and Upper Xiajiadian-created an unprecedented number of sites and material remains in the West Liao River valley. Sites of the Lower Xiajiadian culture number 2,964[10], and large towns -sometimes heavily fortified - emerged for the first time, in the West Liao River valley[20]. In addition, the distribution of sites shifted southward within and beyond the river valley; pottery shapes and forms suggest strong connections with, and influence from, the Longshan Culture in the Central Plain; stone agricultural tools are abundant; and there are many large storage pits -containing carbonized millets -and domesticated animals (cattle, sheep, pigs, and dogs)[27]. At Dadianzi, a Lower Xiajiadian site, archaeologists unearthed nearly 1,000 graves and found pigs or dogs buried in most of them. Pigs are omnivores and eat C_4 plants, making them an indicator of millet production[27]. At Erdaojingzi, another Lower Xiajiadian site dated to about 4,000 BP, broomcorn and foxtail millets account for respectively 16.5% and 72.6% of the 250,419 domesticated plant seeds at this site[10]. Recent isotope analyses suggest that millet was the main daily food of the Lower Xiajiadian people's diet[17].

In the Upper Xiajiadian, large storage pits with carbonized millets and domesticated animals (60% pigs, 11% dogs, 11% sheep and 11% cattle at the Dashanqian site in Chifeng) indicate a heavy reliance heavily on millet farming. However, unlike the Lower Xiajiadian people who relied strongly on agriculture, the Upper Xiajiadian populations adopted herding strategies and maintained a certain mobility in their lives.

1.2.2. Archaeological context of sites from the WLR in this study

Banlashan site (Hongshan Culture)
Banlashan cemetery is located at the top of Banla mountain south of Chaoyang city, Liaoning. The cemetery was uncovered in the third national cultural relics census in 2009. From 2014 to 2016, Liao Provincial Institute of Cultural Relics and Archaeology and Longcheng District Museum of Chaoyang city conducted a cooperative rescue excavation on this cemetery. A total of 78 burials, 1 altar and 29 sacrifice pits were excavated. A large number of artifacts were unearthed including jade dragons which are characteristic of the Hongshan culture. Three types of burials were found in this cemetery namely earthen, sarcophagus and stone pits. Most of the human skeletal remains from this cemetery were not well preserved and some bones were covered with red mineral. Two individuals yielded radiocarbon dates, one from the excavators, BLSM4: 3,355-3,095 calBCE (4510±30 BP) and the other individual from this study (BLSM45)[28]. Nineteen samples from this site were initially screened and only three individuals (Data Table S1) were analyzed in this study.

- BLSM27S: Female, 25-30 years old (5500-5000BP)
- BLSM41: Male, 40-45 years old (5500-5000BP)
- BLSM45: Female, 25-30 years old, 3338-3098 calBCE (4497±30 BP)

Haminmangha site (Hamin Culture)
The Haminmangha site is located about 20 km southeast of Xibet Town in Horqin Left Middle Banner, Inner Mongolia. Since 2011 two seasons of excavations have been conducted by the Inner Mongolian Institute of Cultural Relics and Archaeology and the Research Center for Chinese Frontier Archaeology of Jilin University: 29 house foundations, 10 ash pits, three tombs and one moat were excavated with more than 1,000 artifacts including pottery, jades and implements made of stone, bone and shell. The Haminmangha site is a large-scale prehistoric settlement with unique cultural features (unique combination of pottery and distinctive regional decorations) which are different from those of the nearby archaeological cultures, giving rise to the term 'Hamin Culture'[29]. The most striking feature of the Haminmangha site is the large number of human skeletons excavated in the house foundations, such as F40, where 97 human skeletons were disorderly scattered. Owing to the collapse of the burnt wooden structural members in the roof, part of the skulls and the limb bones had been burnt to black or deformed by pressure. Six individuals were initially screened but to the preservation of those samples, only one individual produced enough genomic data for analysis.

- HMF32: 3694-3636 calBCE (4866±30 BP)

Erdaojingzi site (Lower Xiajiadian Culture)
The Erdaojingzi site lies to the north of the village of Erdaojingzi in the Hongshan District in Chifeng, Inner Mongolia, and encompasses an area of around 30,000 m^2. In 2009, a rescue excavation was conducted on the site by the Inner Mongolia Cultural Relics and Archaeology Institute, during which some 5,200 m^2 was uncovered. A total of 149 house foundations have been discovered at the site and the majority of them are surface structures. Later houses were usually built on the abandoned earlier ones. The lower structures may have been used as the foundations for the houses above them. At one location, seven layers of house remains were found. Abundant artifacts were excavated including a small number of jades and bronzes and a large quantity of pottery and stone and bone artifacts. The Erdaojingzi site is the best -preserved and most intact Lower Xiajidian culture site so far excavated. Five individuals from the Erdaojing site were screened and 3 samples produced enough genomic data for analysis:

- EDM124: 1664-1543 calBCE (3330±30 BP)
- EDM139: 3970-3294BP
- EDM176: 3970-3294BP

Longtoushan site (Upper Xiajiadian Culture)
The Longtoushan site lies about 6 km south of Tuchengzi town, Hexigten, Chifeng, Inner Mongolia. The site was first uncovered in 1986 and was excavated by the Institute of Cultural Relics of Inner Mongolia in 1987. Three out of four individuals screened were further analyzed and one individual was AMS dated in this study.

- 91KLH11: 3000-2300BP
- 91KLH18: 901-825 calBCE (2717±30 BP)
- 91KLM2: 3000-2300BP

1.3. The Yellow River Valley

The Yellow River (Chinese: Huang He) is the second longest river in China and the sixth longest river in the world. It stretches across China for more than 5,464 km. The river originates in the Bayan Har Mountains in Qinghai province of western China and flows eastwards into the Bohai Sea. The basin has different types of climates: an arid continental monsoon climate in the Upper Yellow River and a semi-humid climate in the Central and Lower Yellow River regions. The average annual temperature in the Yellow River region ranges from 4 to 14 °C. The abundant annual precipitation and the suitable temperature make the Yellow River Basin, especially the Central Plain region, favorable for rain-fed agriculture[30].

1.3.1. The Cultural Sequence of the Yellow River region

The Yellow River region has played a key role in the historical development of societies in China. In this region, complex societies emerged as early as 10,000 BP. The early appearance of agriculture in the Yellow River basin is probably based on nutrient-rich soils and favorable amount of precipitation. Agriculture started in the flood plain of the Yellow River by at least 8,700 BP[1]. A continuous sequence of cultures developed in this region including the Peiligang (9,000-7,000 BP), Cishan (8,000-7,000 BP), Yangshao (7,000-5,000 BP), Dawenkou (5,300-4,400 BP) and Longshan cultures (5,000-4,000 BP).

Peiligang culture

The Peiligang culture was named after the site discovered in Peiligang village, Zhengzhou province in the Yellow River region and was dated from 7,000 to 5,000 BCE. The culture is the earliest Neolithic culture in the middle Yellow River valley and represents the emergence of cereal farming in the region[31]. A general consensus among archaeologists is that the Peiligang culture serves as a key stage for the transition from hunting and gathering to millet based farming[32]. Besides millet farming, Peiligang people also practiced animal husbandry, raising pigs, cattle and possibly poultry. It has been argued that mixed farming of broomcorn millet and rice can be traced back to 7,800 BP in the Peiligang culture of the Middle Yellow River region[33]. However, a recent study revealed that during the Peiligang period, millet-only farming was found in hilly lands while mixed farming was mainly conducted in the alluvial plains[31].

Yangshao culture

The Yangshao culture was discovered in 1921 by Swedish archaeologist Johan Gunnar Andersson. The culture was named after the site first excavated in Yangshao village in Mianchi county, Henan province and spans 2,000 years from 5,000 to 3,000 BCE. The Yangshao influenced a vast geographic region centered on the Central Plain area of the Yellow River valley[3]. Yangshao people relied primarily on millet farming followed by rice as a minor component[13,34]. Besides millets and rice, Yangshao people also grew vegetables and raised livestock, including pigs and cows, and engaged in fishing, hunting and gathering. The most significant feature of Yangshao culture is its painted pottery with a variety of shapes and ornamental designs, many of which came with decorative covers or accessories shaped like animals[35]. Compared with the Peiligang culture, Yangshao houses are larger in size and more sophisticated in design.

Longshan culture

The Longshan culture is a late Neolithic culture in the central and lower Yellow River Basin and

dates from 3,000 to 1,900 BCE. The Longshan culture is known for highly skilled pottery thrown on pottery wheels[36]. The Longshan is referred to as the 'Black Pottery Culture' because of use of highly polished black pottery which is distinct from the reddish pottery used in the Yangshao culture[35,37]. During the Longshan period, the population expanded dramatically and many settlements had rammed earth walls. The Neolithic population in northern China reached its peak during the Longshan phase. The term 'Longshan culture' is a general reference to several regional centers such as Shandong Longshan and Henan Longshan. The Longshan culture also shares similarities with the Liangzhu culture of the lower Yangtze River Basin with respect to the production of unpainted polished black pottery[38]. The Longshan culture marked a transition to the establishment of cities and was a time when moated settlements began to appear. Besides millet, rice farming was already well established by the time of the Longshan.

Qijia culture
The Qijia culture dates between 2,400 and 1,900 BCE and is distributed around the upper Yellow River region of western Gansu and the eastern part of Qinghai province. The culture was named after initial excavations at the Qijiaping site in 1923 by J. G. Andersson. The Qijia culture is also known for the discovery of copper mirrors and domesticated horses as well as the oldest noodles made from millet[39]. The pottery of the Qijia culture is all handmade without any evidence of wheel-made wares[40]. The pottery of the Qijia culture shares many traits with the Longshan culture in Shaanxi and elements of the Majiayao culture but also has its own characteristics.

1.3.2. Archaeological context of sites from the YR in this study

Xiaowu site (Yangshao Culture)
The Xiaowu site is located in Xiaowu village, Lingbao city, Henan province. The site was found in a survey between 2007 and 2009 by Henan Provincial Institute of Cultural Relics and Archaeology. Two tombs were excavated that were identified as belonging to the early Yangshao culture by a trial excavation in 2007. Both of the tombs were rectangular earthen pit burials and a total of 96 human skeletons were collected. Only one individual from this site was AMS dated and produced genomic data in this study.

- XW-M1R18: 4225-3987 calBCE (6056±119 BP)

Wanggou site (Yangshao Culture)
The Wanggou site is located in Wanggou village, Yingyang county, Henan province. It is a large Yangshao culture cemetery dated from 7000 to 5000 BP. Besides human skeletons, a large number of animal bones were also found. Seven individuals produced enough data for analysis in this study.

- WGM20: 5500-5000 BP
- WGM35: 3354-3106 calBCE (5180±124 BP)
- WGM35-1: 5500-5000 BP
- WGM43: 5500-5000 BP
- WGM70: 5500-5000 BP
- WGM76S: 5500-5000 BP
- WGM94: 5500-5000 BP

Wadian site (Longshan Culture)
The Wadian site is located in Yuzhou city, Henan province. It was found in a survey by Henan

Provincial Institute of Cultural Heritage and Archaeology in 1979. Since 1980, three seasons of excavations were carried out by the same institute and more than 1 million square meters were excavated making it the largest Longshan site in Henan province. Recent studies showed that Longshan people kept domesticated pigs, cattle, sheep and dogs. Domesticated animals served as the main source of meat. Large numbers of plant seeds were also found in this site including millet, rice, beans and wheat. Five individuals were initially screened and two of them produced enough genomic data.

- WD-WT1H16: 2239-2033 calBCE (4036±53 BP)
- WD-WT5M2: 4000-3900 BP

Haojiatai site (Longshan Culture/Eastern Zhou)
The Haojiatai site is located in Shicaozhao village, Luohe city, Henan province. Archaeologists have found that the remains excavated at Haojiatai site belonged to the Central Plain Longshan culture, the Xinzhai period, the Erlitou culture and the Eastern Zhou Dynasty. The most important part of the Haojiatai site is the walled city. A total of 14 foundations, 310 ash pits and 90 burials were excavated in this site. Eight samples were screened in this study, among which four individuals produced enough genomic data.

- HJTM107: 2136-1950 calBCE (3660±30 BP)
- HJTM109: 2031-1888 calBCE (3600±30 BP)
- HJTM115: 365-207 calBCE (2236±79 BP)
- HJTW13: 353-57 calBCE (2140±30 BP)

Pingliangtai site (Longshan Culture)
The Pingliangtai site is located in Huaiyang, Henan province and was excavated between 2015 to 2016 when 14 tombs belonging to the Longshan period were found. Eight tombs in alignment constituted a small graveyard. The other six tombs were presumably related to building foundations[41]. Four individuals were analyzed in this study.

- PLTM310: 2275-2035 calBCE (3740±30 BP)
- PLTM311: 2201-2024 calBCE (3710±30 BP)
- PLTM312: 2135-1939 calBCE (3650±30 BP)
- PLTM313: 2118-1894 calBCE (3620±30 BP)

Jiaozuoniecun site
Jiaozuoniecun site was discovered in 2014. A rescue excavation was carried out by Jiaozuo Municipal Institute of Cultural Relics and Archaeology and a total of 48 burials and 19 ash pits were discovered after a period of 2 months' excavation. Based on the relics excavated, archaeologists confirmed that the site belonged to the Shang culture[42]. Three individuals were screened in this study of which two provided enough data for genomic studies and one was AMS dated.

- JXNTM2: ~3000 BP
- JXNTM23: 1231-1123 calBCE (3127±54 BP)

Luoheguxiang site
The Luoheguxiang site is located in Luohe city, Henan province about 5 km south of the Ying county. Between 2012 and 2013, the site was excavated by a joint team of Luohe Municipal Institute of Cultural Relics and Archaeology and Henan Provincial Institute of Cultural Heritage

and Archaeology. More than 150 burials were excavated which spanned four time periods, namely the Warring States period, Han, Song and Ming dynasties. Two individuals produced enough genomic data for analysis.

- LGM41: 2200-2000 BP
- LGM79: 388-230 calBCE (2259±79 BP)

Miaozigou site (Miaozigou Culture)

The Miaozigou site is located at Uraharura, Qahar Youyi Qianqi in south-central Inner Mongolia on the southern coastal region of Lake Huangqihai. The site is 220 meters long and 120 meters wide and covers a region of 10,500 square meters. A total of 52 house foundations and 42 burials as well as large numbers of animal skeletons were excavated. Isotopic analysis suggests that Miaozigou people consumed a significant proportion of animal food and that C_4 plants were their main plant nutritional source in their daily life[43]. Three individuals from this site were analyzed.

- MZGM10-1: ~5500 BP
- MZGM16: ~5500 BP
- MZGM25-2: ~5500 BP

Lajia site (Qijia Culture)

The Lajia site is located in Lajia village, Minhe County, Qinghai province, Northwest China. The site became well-known because of the discovery of the earliest intact noodles that were made from millet. The site was estimated to be around 4,000 years old[44]. Lajia people had domesticated sheep, pig and cattle with sheep as the most common species. Many archaeologists believe that the site was abandoned after being devastated by an earthquake and subsequent flood; multiple individuals excavated inside the buildings may have been refugees at the moment of this natural disaster[44]. Six individuals from Lajia produced enough genomic data.

- LJM2: 4000-3800 BP
- LJM3: 2125-1959 calBCE (3996±83 BP)
- LJM4: 4000-3800 BP
- LJM5: 4000-3800 BP
- LJM14: 4000-3800 BP
- LJM25: 4000-3800 BP

Jinchankou site (Qijia Culture)

The Jinchankou site is located in Huzhu county, Qinghai province at an elevation of 2,309 meters. In 2012, a rescue excavation was carried out by Qinghai Provincial Institute of Archaeology. Five house foundations, 15 ash pits and large quantities of pottery as well as jade artifacts were found. One individual produced enough data in this study.

- JCK-M1-1: 1948-1884 calBCE (3866±32 BP)

Dacaozi site

The Dacaozi site is located in Pingan county, Qinghai province. In 2013, Qinghai Provincial Institute of Archaeology carried out excavations and a total of 23 burials and 84 human remains were excavated. Based on the relics excavated, the site was estimated to date from the Eastern Han to Northern Wei periods[45]. Three out of seven individuals were better preserved and were sequenced to a higher coverage.

- DCZ-M17IV: East Han (~2000BP)

- DCZ-M21II: 68-128 calCE (1852±30 BP)
- DCZ-M22IV: East Han (~2000BP)

Shengedaliang site
The *Shengedaliang* site is located in Shenmu in the northern part of the Loess Plateau. The site is dated from the Late Neolithic (Middle Longshan period) to the Xia period[46]. Various features were observed in the site, such as tombs, ash pits, house foundations and rammed-earth foundation. Nine individuals were screened in this study and three of them produced enough data.

- SM-SGDLM27: ~4000 BP
- SM-SGDML6: 2193-2035 calBCE (4064±79 BP)
- SM-SGDML7X: ~4000 BP

Supplementary Note 2. Additional results for the genetic analysis

Genetic relatives among ancient northern China individuals. Pairwise mismatch analysis shows that the mismatch rate between unrelated individuals is around 0.24. Three individuals from the Banlashan site (BLSM27S, BLSM41 and BLSM45) showed a relatively lower level of mismatch rate with a value of ~0.21, close to the value expected for second-degree relatives (Supplementary Fig. 2 and Supplementary Table 8). Three individuals from the Pingliangtai site (PLTM310, PLTM311 and PLTM312) also showed a relatively low value for different pairs between them and all of those 3 individuals are unrelated from the fourth individual (PLTM313) from the same site (pairwise mismatch rate: ~0.24). PLTM311 and PLTM312 showed an even reduced pairwise mismatch rate of 0.19, suggesting that they are first-degree relatives. Considering that they are infants and share the identical mitochondrial haplogroup (D4b1a), we conclude that they are siblings. Two individuals from the Lajia site (LJM3 and LJM5) also show first-degree relatedness. Pairwise mismatch rate between PLTM310 and PLTM311/PLTM312 is around 0.21, suggesting a second-degree relatedness. Likewise, three individuals from the Mogushan site also show some extent of first- and second-degree relatedness (first-degree: MGS-M7L/MGS-M7R; second-degree: MGS-M6/MGS-M7L, MGS-M6/MGS-M7R) (Supplementary Fig. 2). The above relative pairs are also detected as relatives from the *lcMLkin* analysis by having high values of coefficients of relationship (Supplementary Fig. 3 and Supplementary Table 9). To prevent bias in population-level analysis due to the inclusion of related individuals, we kept an individual with higher coverage for each of the first-degree relative pairs for most analyses (except for individual-based ADMIXTURE and PCA).

Genetic substructure among Tungusic-speaking populations in AR. We show that ancient AR individuals are genetically closest to each other, forming a clade within the resolution of our data (Supplementary Figs. 4, 6-10, 20-21). Six of them form a tight cluster with the biggest coordinates on the Eurasian PC2: two Early Neolithic hunter-gatherers ("AR_EN") and three Iron Age individuals ("AR_Xianbei_IA"; 2nd century CE; Xianbei context) from the Upper AR, and one Bronze Age WLR individual from a nomadic pastoralist context ("WLR_BA_o"). They fall within the range of present-day AR populations, which mostly speak Tungusic languages with the exception of Nivkh (Supplementary Fig. 4). Ancient and present-day AR individuals are also similar in their genetic profiles from the model-based genetic clustering (Supplementary Fig. 6). AR males exclusively harbor Y haplogroup C2b1a (Supplementary Table 2), a haplogroup reaching high frequency in historical nomadic groups such as Donghu, Xianbei, Rouran and Shiwei as well as in present-day Mongolic- and Tungusic-speaking populations from the region and nearby Mongolia. We tested long-term genetic stability in the AR region in the strongest sense by testing cladality using f_4-statistics of the form f_4(AR$_1$, AR$_2$; X, Mbuti) for all pairs of ancient and present-day AR populations (AR$_1$ and AR$_2$) against 20 representative world-wide outgroup populations (Supplementary Fig. 9). Nearly all pairs of ancient AR individuals, including the most recent Iron Age individuals (AR_Xianbei_IA and "AR_IA"), show no significant values ($|f_4| < 3$ s.e.m.; Supplementary Fig. 9), supporting genetic continuity from Neolithic to Iron Age within the resolution of our data. AR genetic stability in general continues to present-day populations in the region, such as Nanai, Negidal, Ulchi and Nivkh, except for their relationship with the two Siberian outgroups, Nganasan and Itelmen (Supplementary Fig. 10). Tungusic-speaking groups further to the north (Evenk and Even) significantly break symmetry in many tests, suggesting a genetic stratification within present-day Tungusic speakers

(Supplementary Figs. 9-10). Although still closely related, they show some extent of genetic difference with respect to their relationships with present-day populations outside of the AR region, such as Itelmen and Nganasan (Supplementary Fig. 10). Also, Tungusic-speaking population further to the south, notably Oroqen, Hezhen and Xibo, do not form a clade with the AR ones (Supplementary Fig. 9). To quantify these differences, we performed qpAdm-based admixture modeling of present-day Tungusic- and Nivkh-speaking populations as well as ancient populations in the nearby regions: Early Neolithic individuals from the Russian Far East (Devil's Gate), Neolithic and Bronze Age individuals from the Baikal region (Baikal_EN and Baikal_EBA), and a RouRan individual from Mongolia. We tried two-way admixture models of AR_EN and MA-1 / Nganasan / WLR_LN. As suggested by the f_4 statistics (Supplementary Fig. 9), present-day AR populations and ancient individuals, Devil's Gate and RouRan, are adequately modeled by a single source AR_EN. Ancient Baikal populations and present-day Siberian Tungusic-speaking populations (Evens and Evenk_Transbaikal) are adequately modeled with extra ANE ancestry (either MA-1 or Nganasan) (Supplementary Table 10). Finally, Tungusic-speaking populations further to the south from AR, Oroqen, Hezhen and Xibo, require a substantial amount of WLR_LN-related ancestry (29-73%; Supplementary Table 10).

A previous study reported early Neolithic genomes from the Lake Baikal region ("Baikal_EN"), showing a near complete genetic turnover from the Upper Paleolithic ancestry in the region, often called "Ancient North Eurasian" (ANE). Interestingly, Baikal_EN are scattered between the Baikal individuals in later periods ("Baikal_EBA") and Neolithic AR individuals in PCA (Supplementary Fig. 4). F_4 statistic shows that Baikal_EN are closer to ANE-related individuals than AR individuals are, most notably to Mesolithic Eastern European Hunter-Gatherers ("EHG"; Z = 3.0 s.e.m.) and Eneolithic Botai (Z = 3.2 s.e.m.; Supplementary Fig. 21). Relying on *qpAdm,* which summarizes multiple f_4 statistics to model details of admixture, we model Baikal_EN as a mixture of AR_EN and Pleistocene southern Siberian individual MA-1 (p = 0.582) with 9.6±1.9% (estimate ± 1 standard error measure, s.e.m.) contribution from MA-1.

Analysis of phenotype- or selection-associated variants. We investigated genetic variants that are well known for harboring signatures of selective sweep or genotype-phenotype association in the following genes: *EDAR* (ectodysplasin A receptor), *ABCC11* (ATP binding cassette subfamily C member 11), *LCT* (lactase), *ADH1B* (alcohol dehydrogenase 1B), *OCA2* (Oculocutaneous albinism II), *MC1R* (Melanocortin 1 receptor), *EGLN1* (Egl-9 family hypoxia inducible factor 1), and *EPAS1* (Endothelial PAS domain protein 1). For the candidate SNPs in the above genes, we tabulated the number of reads matching with reference and alternative alleles for 15 of 55 individuals with at least 2x genome-wide coverage (Supplementary Table 11). We only include reads with a mapping score of at least 30 and sites with a minimum base quality of 30 into analysis. Same as the genome-wide analysis, we used masked BAM files for C/T and G/A SNPs and unmasked ones for the remaining SNPs. We find no known allele associated with lactase persistence (0/436 reads across 8 known variants), suggesting that all 15 individuals from northern China were probably genetically lactose intolerant in adulthood. As expected from its high frequency in both East Asians and Native Americans[49], the derived G allele of rs3827760 in the *EDAR* gene is already prevalent in our samples (50/51 reads). It is known to be associated with straighter, thicker hair, and shovel-shaped incisors[50]. The derived T allele of rs17822931 in the *ABCC11* gene is associated with dry earwax phenotype as well as sweat production[51] and is commonly found among present-day East Asians. The ancient northern

China individuals also show the derived *ABCC11* allele in high frequency (35/35 reads). We also confirm the ancient presence of some derived alleles that are currently confined mostly within East Asia, such as rs1229984 (*ADH1B*; 3/31 reads from two individuals), rs1800414 (*OCA2*; 7/49 reads from four individuals) and rs2228479 (*MC1R*; 11/104 reads from four individuals). Finally, as some individuals (such as individuals from the Lajia and Jinchankou sites) were excavated from sites near the northeast part of the Tibetan Plateau, we further checked if our ancient individuals share high-altitude adaptive genetic variants with the Tibetans. Specifically, we inspected whether our ancient individuals share derived alleles with 19 SNPs in the *EPAS1* gene[52] and two nonsynonymous SNPs in the *EGLN1* gene[53,54]. Only two out of 921 reads aligned to the *EPAS1* SNPs match with derived alleles. The two derived reads are from a single C-to-T SNP in one individual (rs74898705; 2/10 reads for XW-M1R18) and this individual does not harbor any derived allele in the tightly-linked remaining 16 SNPs (0/108 reads). Therefore, we believe that these two derived reads are likely to be from residual post-mortem damage rather than from a real derived variant. In contrast, we observe derived *EGLN1* nonsynonymous alleles in multiple individuals: rs12097901 (21/56 reads from 6 individuals) and rs186996510 (2/42 reads from two individuals; SM-SGDLM6 from Shengedaliang and BLSM27S from Banlashan). The latter is rare outside of the Tibetan plateau (3% frequency in 1000 the Genomes East Asians) and is known to have arisen on the background haplotype including rs12097901 which is common in East Asians (46% in the 1000 Genomes East Asians). Additional samples with deeper sequence coverage are required to provide insight into the geographic and temporal origins of rs186996510.

Archaeobotanical analysis of millet and rice agriculture in YR and Yangtze river basins. Farming became the most important subsistence strategy since the early Yangshao period (7,000-6,000 BP) in China, Agriculture based on rice and millet cultivation dominated in southern and northern China respectively during the Yangshao and later Longshan periods[55]. By collecting and summarizing the up-to-date published data of crop macro-fossils assemblage from archaeological sites between 7,000-4,000 BP in China[56-115], we found that rain-fed agriculture was the primary livelihood in both the YR and WLR basins between 7,000-4,000 BP, while rice-farming spread northward from the Yangtze river basin to the YR during the Longshan period, When it was adopted as an important subsistence strategy in the middle and lower YR between 4,500-4,000 BP (Supplementary Fig. 12). Considering our current ancient DNA findings of a significant genetic contribution from populations that related to south China (or maybe Southeast Asia) between the Yangshao and Longshan periods, our archaeobotanical evidences strongly suggest that the northward expansion of rice agriculture in the Late Neolithic might have been accompanied by human migrations from south China, and which might have contributed to human genetic pools in the YR since that time.

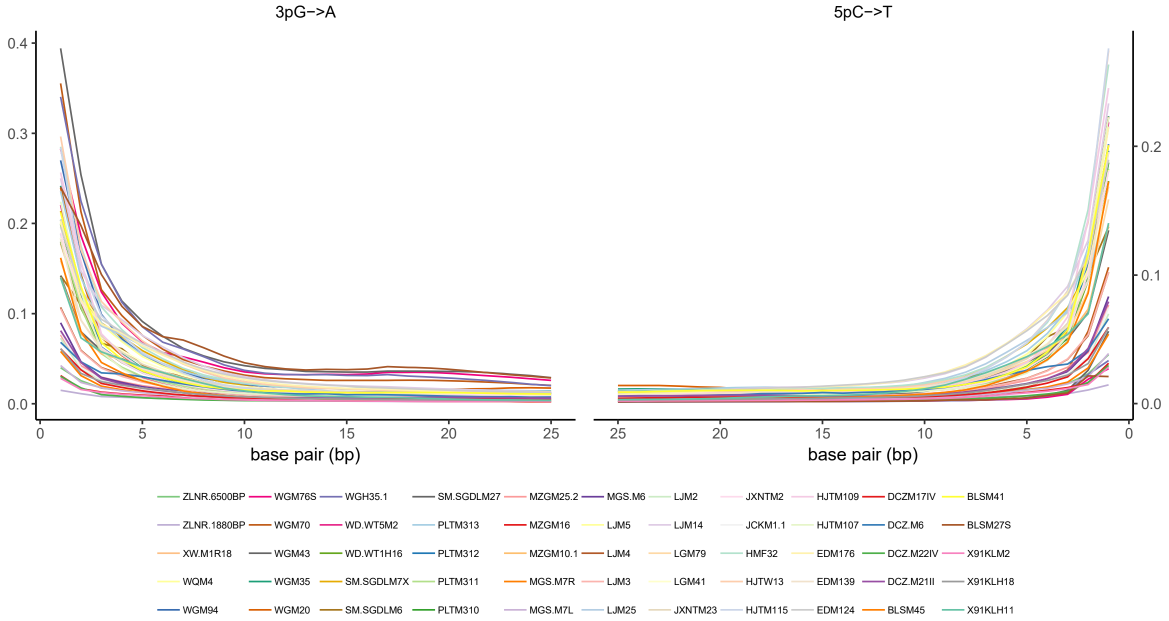

Supplementary Fig. 1. DNA damage plots for 55 ancient samples from northern China. Curves on the left side represent C->T misincorporations at the 5' end and those on the right side represent G->A misincorporations at the 3' end.

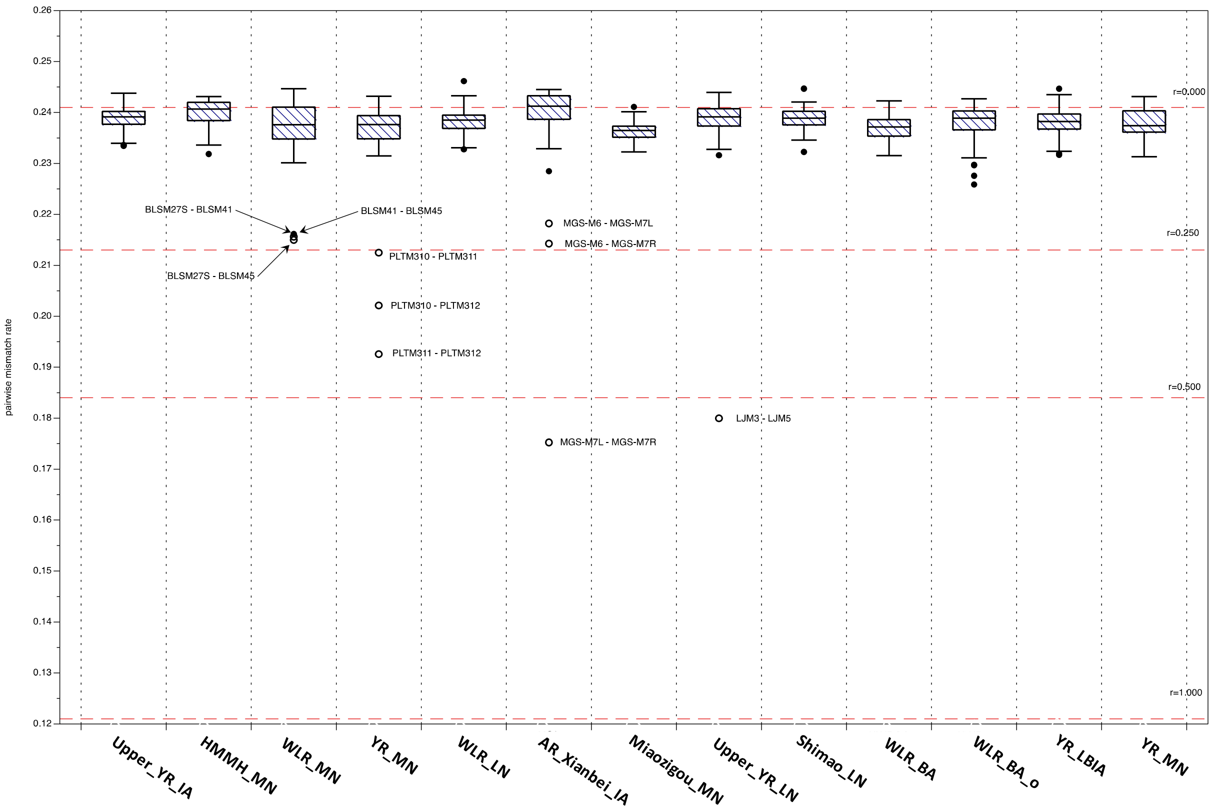

Supplementary Fig. 2. Pairwise mismatch rate across all pairs of ancient individuals from northern China. The red dashed lines from the bottom to the top mark the expected pairwise mismatch rate for the identical twins (r=1), first-degree (r=0.5), second-degree (r=0.25) and unrelated (r=0) individual pairs. Here we keep individual pairs with at least 8,000 SNPs covered by both individuals to reduce artificial bias due to high missing rates. Each box-plot shows distribution of 54×N comparisons of the 'pmr' value (N = number of ancient individuals in each group; Table 1). The horizon black line within the box of each box-plot represents the median value of the distribution, and the vertical black line delineates 1.5 times the distance between the 25th and 75th quartile of the distribution. Black dots denote the outlier and the circles represent the extreme outliers.

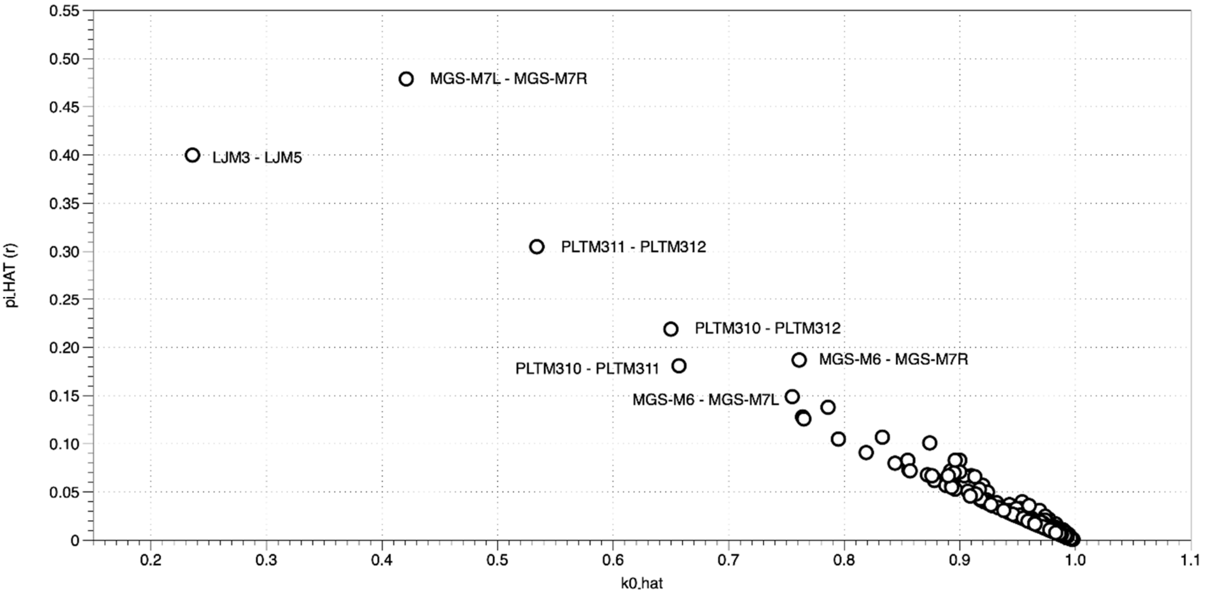

Supplementary Fig. 3. lcMLkin estimates genetic relatedness between pairs of ancient individuals from this study. "k0_hat" represents the probability of two diploid individuals sharing 0 alleles with recent shared ancestor ("identity-by-descent") and pi_HAT (r) represent the coefficient of relatedness. In this sense, individuals with lower k0_hat but higher pi_HAT tends to have closer kinships.

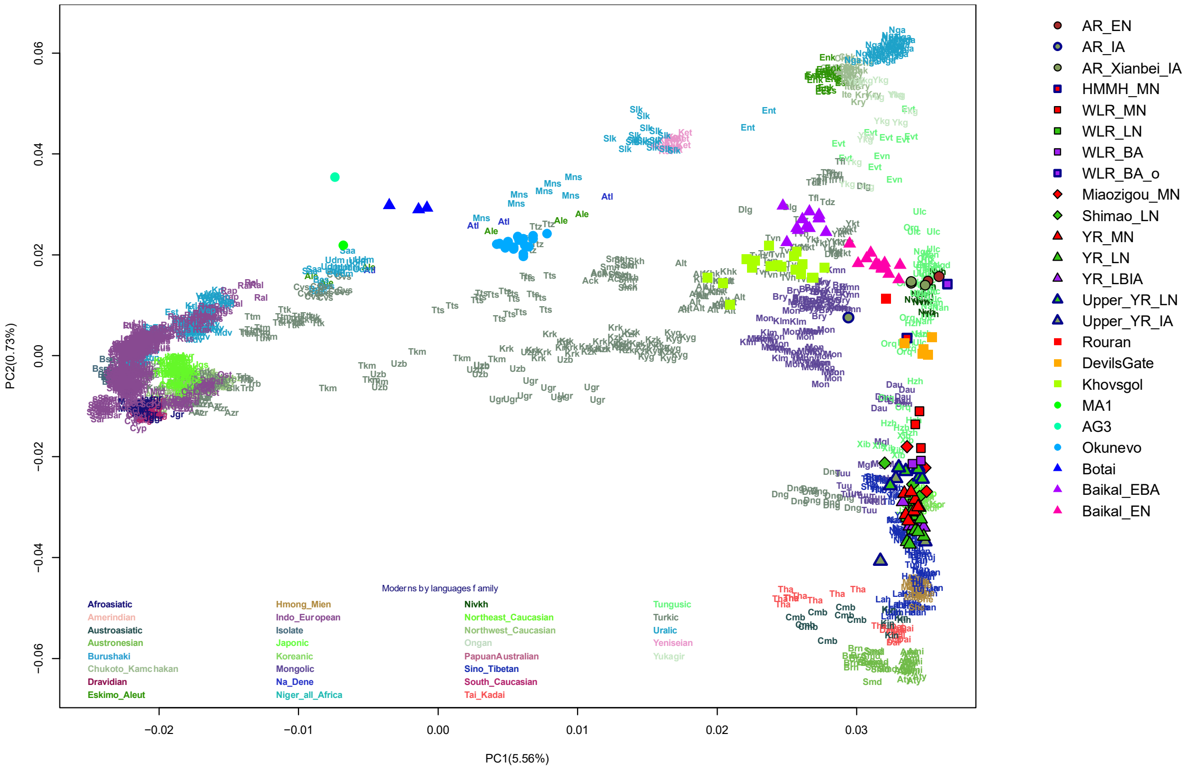

Abz Abazin
Abk Abkhasian
Adg Adygei
Abn Albanian
Ale Aleut
Atl Aleut_Tlingit
Alt Altaian
Ack Altaian_Chelkans
Ami Ami
Arm Armenian
Ahm Armenian_Hemsheni
Aty Atayal
Avr Avar
Azr Azeri
Blk Balkar
Bsc Bashkir
Bsq Basque
Blr Belarusian
Bes Besermyan
Brn Borneo
Blg Bulgarian
Bry Buryat
Cmb Cambodian
Cch Chechen
Chk Chukchi
Cvs Chuvash
Ccs Circassian
Crt Croatian
Cyp Cypriot
Cze Czech
Dai Dai
Drg Darginian
Dau Daur
Dlg Dolgan
Dng Dungan
Ent Enets
Eng English
Ecs Eskimo_CS
Enk Eskimo_NK
Est Estonian
Evn Even
Evf Evenk_FarEast
Evt Evenk_Transbaikal
Ezd Ezid
Fin Finnish
Fre French
Ggz Gagauz
Grg Georgian
Ger German
Grk Greek
Han Han
Hzh Hezhen
Hun Hungarian
Ice Icelandic
Igs Ingushian
Iri Irish
Iru Irish_Ulster
Itn Italian_North
Its Italian_South
Ite Itelmen
Jap Japanese
Jas Jew_Ashkenazi
Jgr Jew_Georgian
Kbd Kabardinian
Ktg Kaitag
Klm Kalmyk
Krc Karachai
Krk Karakalpak
Krl Karelian
Kzk Kazakh
Ket Ket
Khs Khakass
Khk Khakass_Kachins
Kmn Khamnegan
Kin Kinh
Kor Korean
Kry Koryak
Kbc Kubachinian
Kmk Kumyk
Krd Kurd
Kyg Kyrgyz
Lah Lahu
Lak Lak
Lzg Lezgin
Lth Lithuanian
Mlt Maltese
Mns Mansi
Mia Miao
Mld Moldavian
Mon Mongol
Mgl Mongola
Mdv Mordovian
Nan Nanai
Nax Naxi
Ngd Negidal
Nga Nganasan
Nvh Nivh
Ng1 Nogai
Nwg Norwegian
Orc Orcadian
Orq Oroqen
Ost Ossetian
Pol Polish
Rom Romanian
Rus Russian
Rak Russian_Krasnoborsky
Ral Russian_Leshukonsky
Rap Russian_Pinezhsky
Saa Saami
Sar Sardinian
Sct Scottish
Slk Selkup
Smd Semende
She She
Shp Sherpa
Stl Shetlandic
Skh Shor_Khakassia
Smn Shor_Mountain
Scl Sicilian
Srb Sorb
Spa Spanish
Spn Spanish_North
Tbs Tabasaran
Tjl Tajik
Ttk Tatar_Kazan
Ttm Tatar_Mishar
Tts Tatar_Siberian
Ttz Tatar_Zabolotniye
Tha Thai
Tib Tibetan
Tdz Todzin
Tfl Tofalar
Tuu Tu
Tbl Tubalar
Tuj Tujia
Tra Turkish
Trb Turkish_Balikesir
Tkm Turkmen
Tvn Tuvinian
Udm Udmurt
Ukr Ukrainian
Ulc Ulchi
Ugr Uygur
Uzb Uzbek
Vep Veps
Xib Xibo
Ykt Yakut
Yii Yi
Ykg Yukagir

Supplementary Fig. 4. Eurasian PCA showing the genetic profile of ancient individuals from northern China and nearby regions. We calculated PCs using 2,077 present-day Eurasian individuals (marked by three-letter codes) and projected ancient individuals onto the top two PCs. Present-day individuals are color-coded based on the language family they belong to. Ancient individuals newly reported in this study are marked by color-filled shapes with black boundary. Published ancient individuals from nearby regions are marked by color-filled shapes without boundary (Supplementary Data 2).

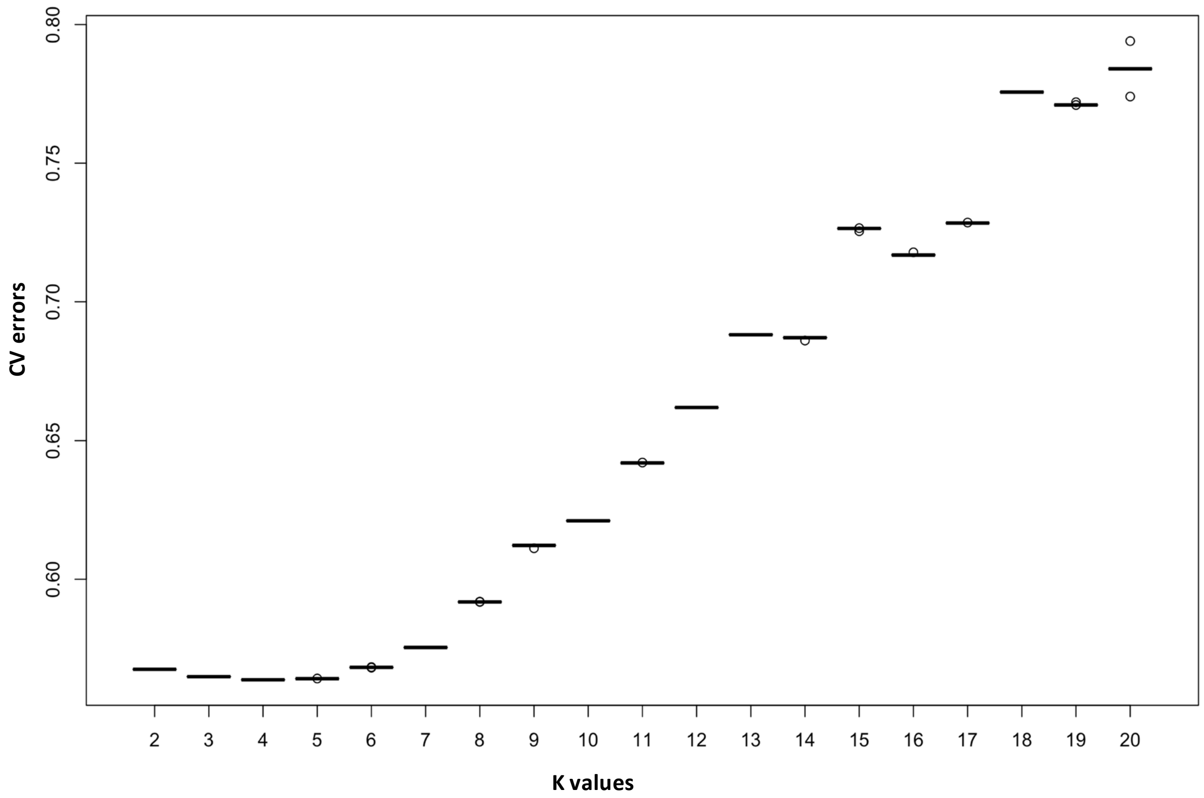

Supplementary Fig. 5. Cross-validation (CV) errors for the ADMIXTURE analysis. We ran five replicates for each K value and the lowest CV errors are observed for K=2 to K=5.

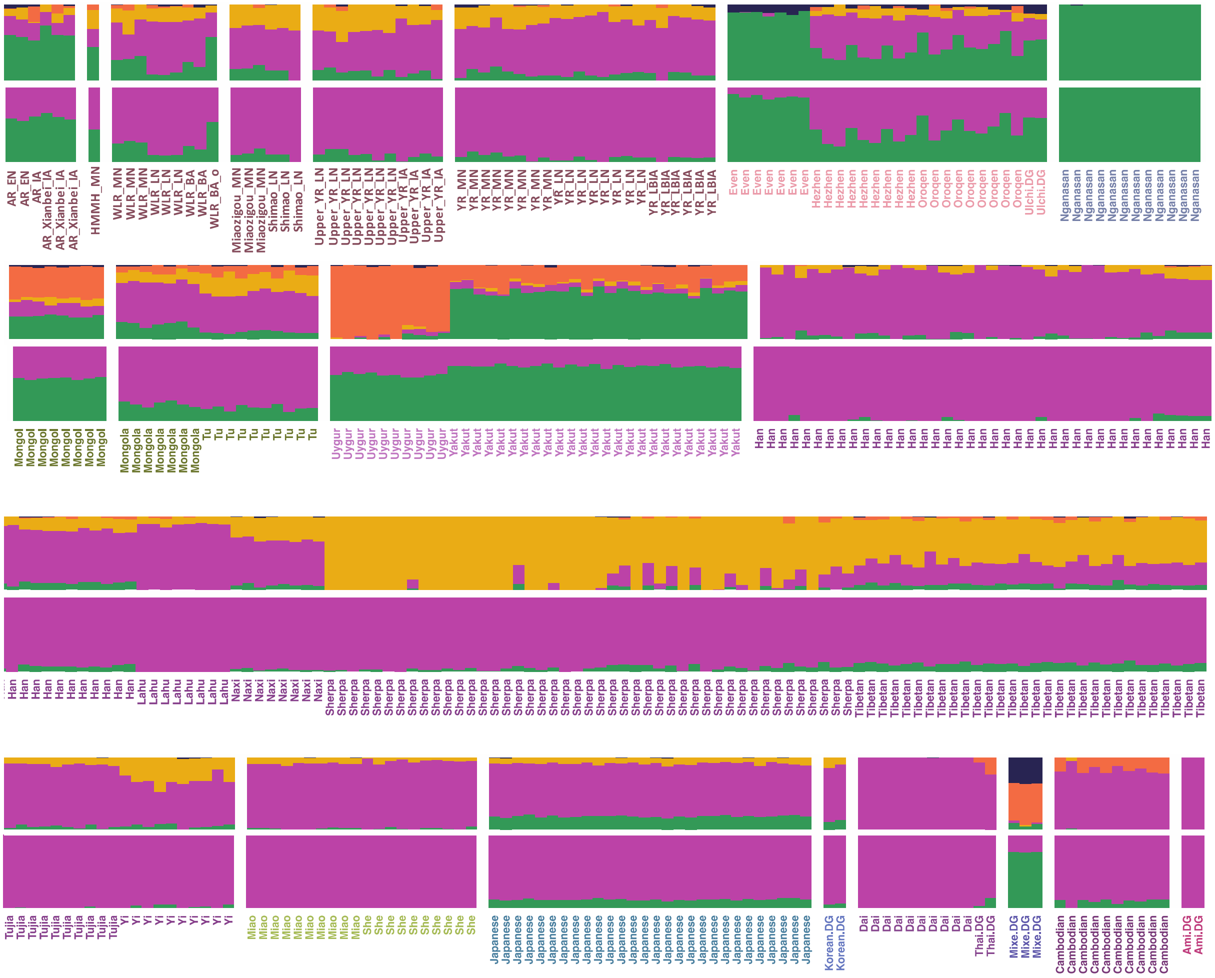

Supplementary Fig. 6. Unsupervised ADMIXTURE analysis of selected ancient and present-day populations. We present results for K = 2 and 5 (lower and upper rows, respectively). The upper two rows show ancient individuals and the lower four rows show selected present-day East Asian, Siberian and Native American populations.

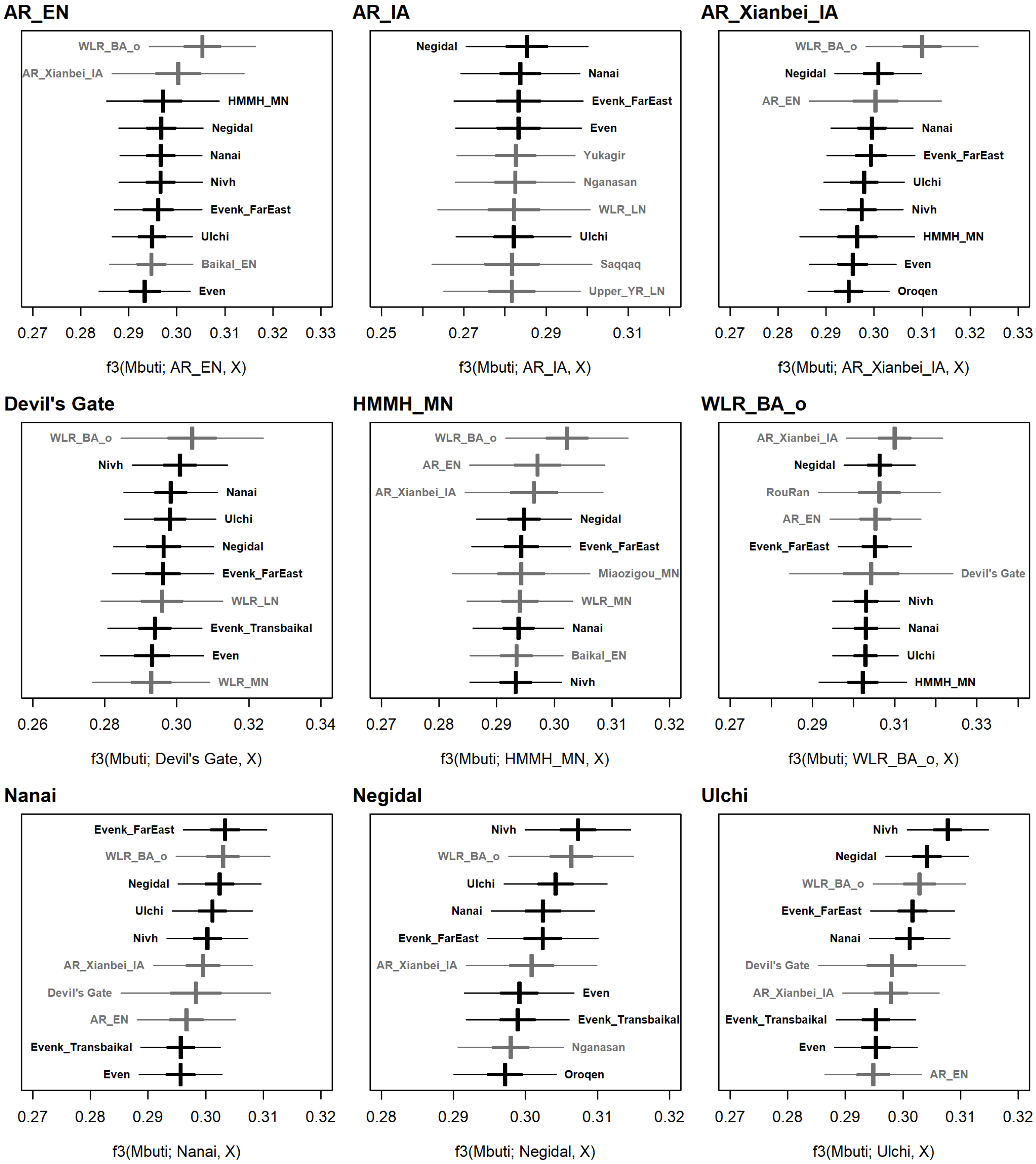

Supplementary Fig. 7. Close genetic relationship between ancient and present-day populations from the Amur River and nearby regions. We present top 10 outgroup-f_3 signal for each population among 334 non-sub-Saharan African populations. Horizontal bars represent the point estimate ± 3 (thin) and ± 1 (thick) s.e.m, respectively, and s.e.m. are estimated using 5 cM block jackknifing. Populations from the Amur River regions are marked in black color, and those outside of the region are marked in grey.

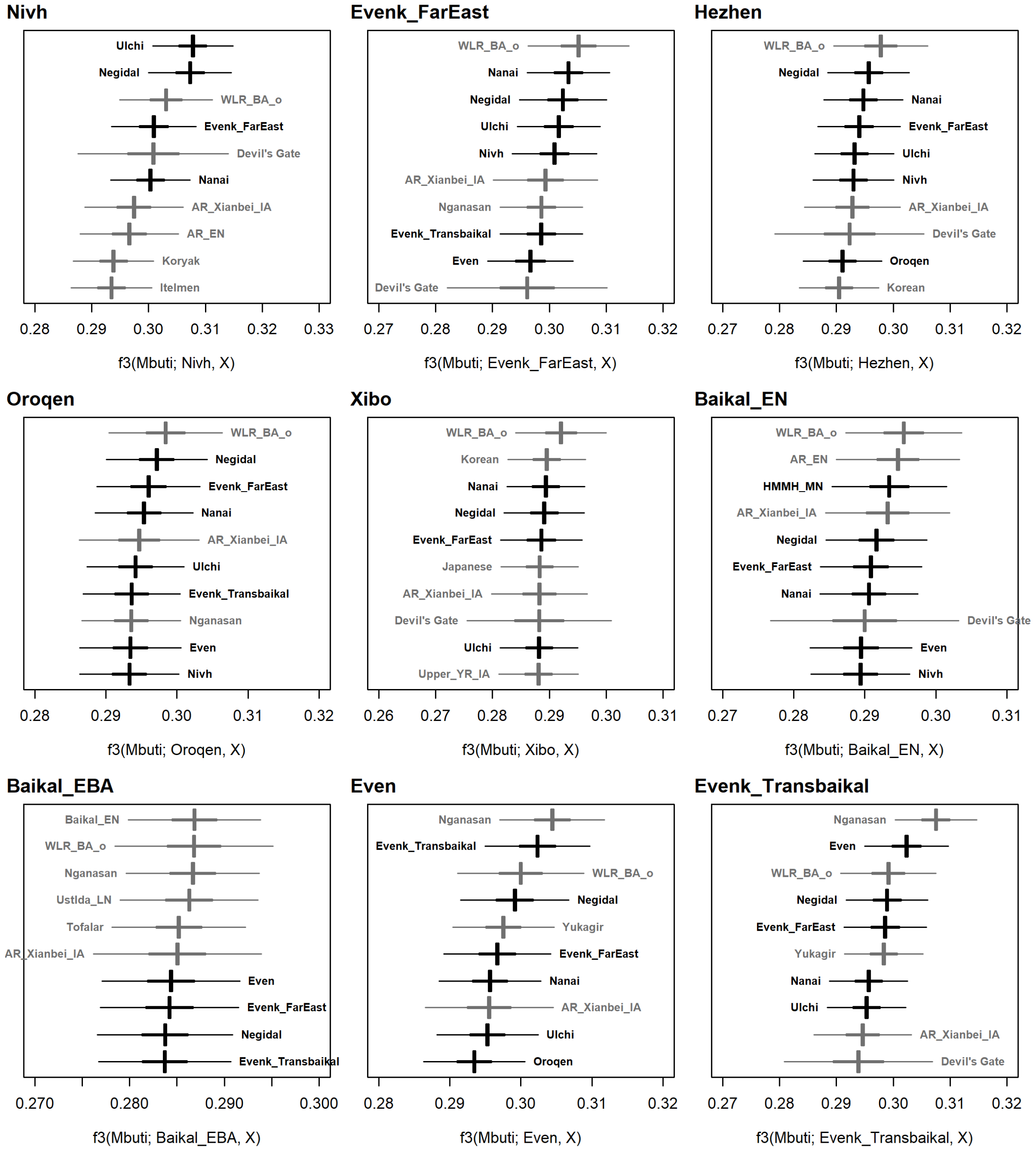

Supplementary Fig. 7. (*Continued*)

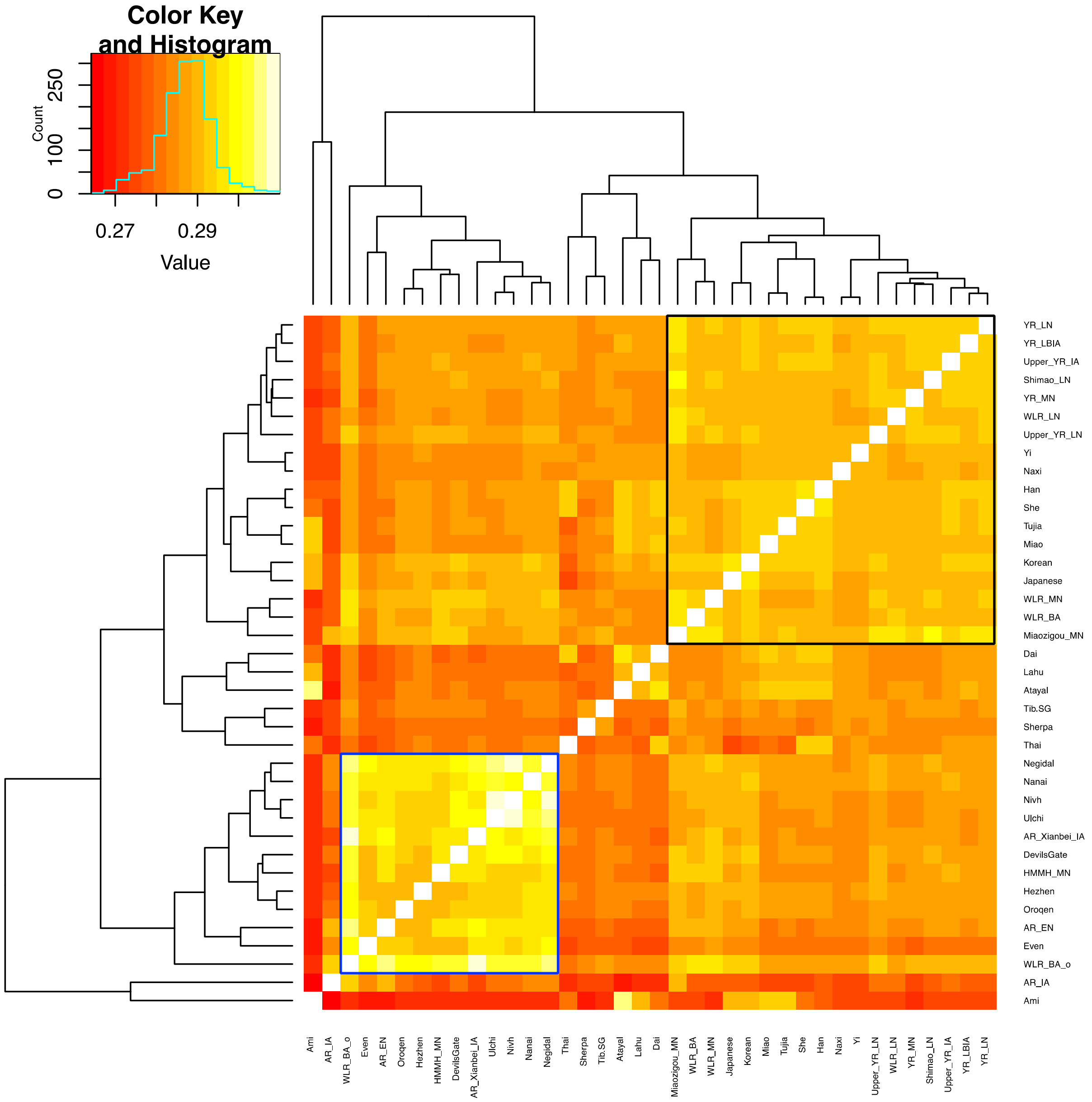

Supplementary Fig. 8. A heatmap summary of the outgroup f_3-statistics for pairs of ancient and present-day East Asian populations. Both ancient individuals from this study and present-day East Asian populations are included. Blue rectangle indicates the AR cluster and the black one shows the YR cluster. High genetic affinity between the AR populations are represented by elevated outgroup-f_3 values.

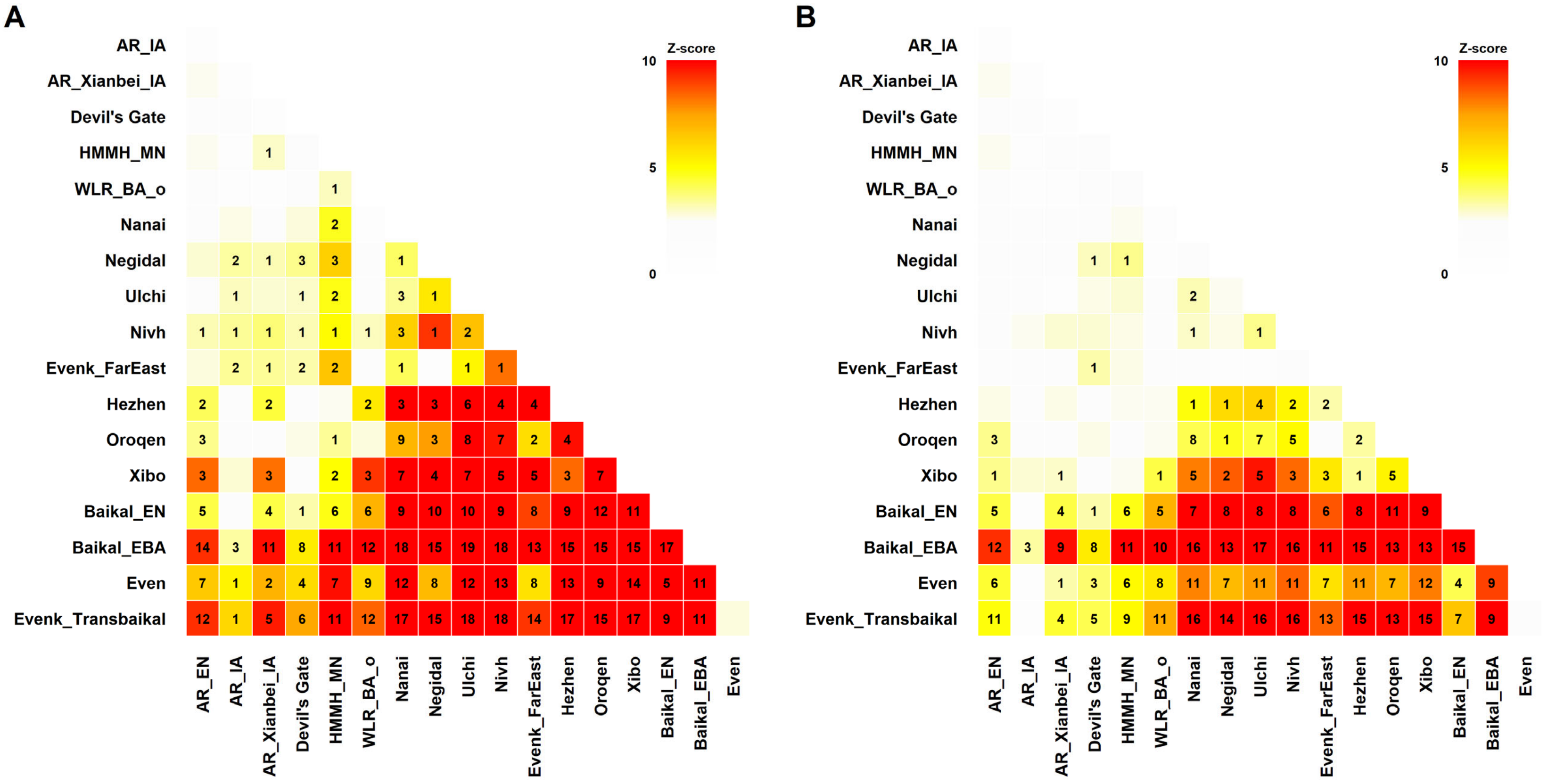

Supplementary Fig. 9. Long-term stability of the genetic profile in the AR region measured by f_4 symmetry statistics. (A) We calculated f_4 symmetry statistics in the form of f_4 (AR_1, AR_2; X, Mbuti) for 20 representative world-wide populations outside of the AR region: Ami, Armenian, BedouinB, Dai, French, Itelmen, Japanese, Korean, Mixe, Nganasan, Onge, Papuan, Sardinian, Tibetan, EHG, LBK_EN, MA-1, Tianyuan, WHG, Yamnaya_Samara. Heatmap colors represent the biggest |Z| scores among 20 tests. Z scores were calculated by dividing f_4 statistic by its corresponding 5 cM block jackknifing s.e.m.. Numbers inside each cell show the number of tests with $|Z| \geq 3$ s.e.m. (B) We summarize the same statistics after excluding two Siberian outgroups, Nganasan and Itelmen, from the list of 20. Excluding these two outgroups, most ancient and present-day populations from the AR region show no significant departure from cladeness.

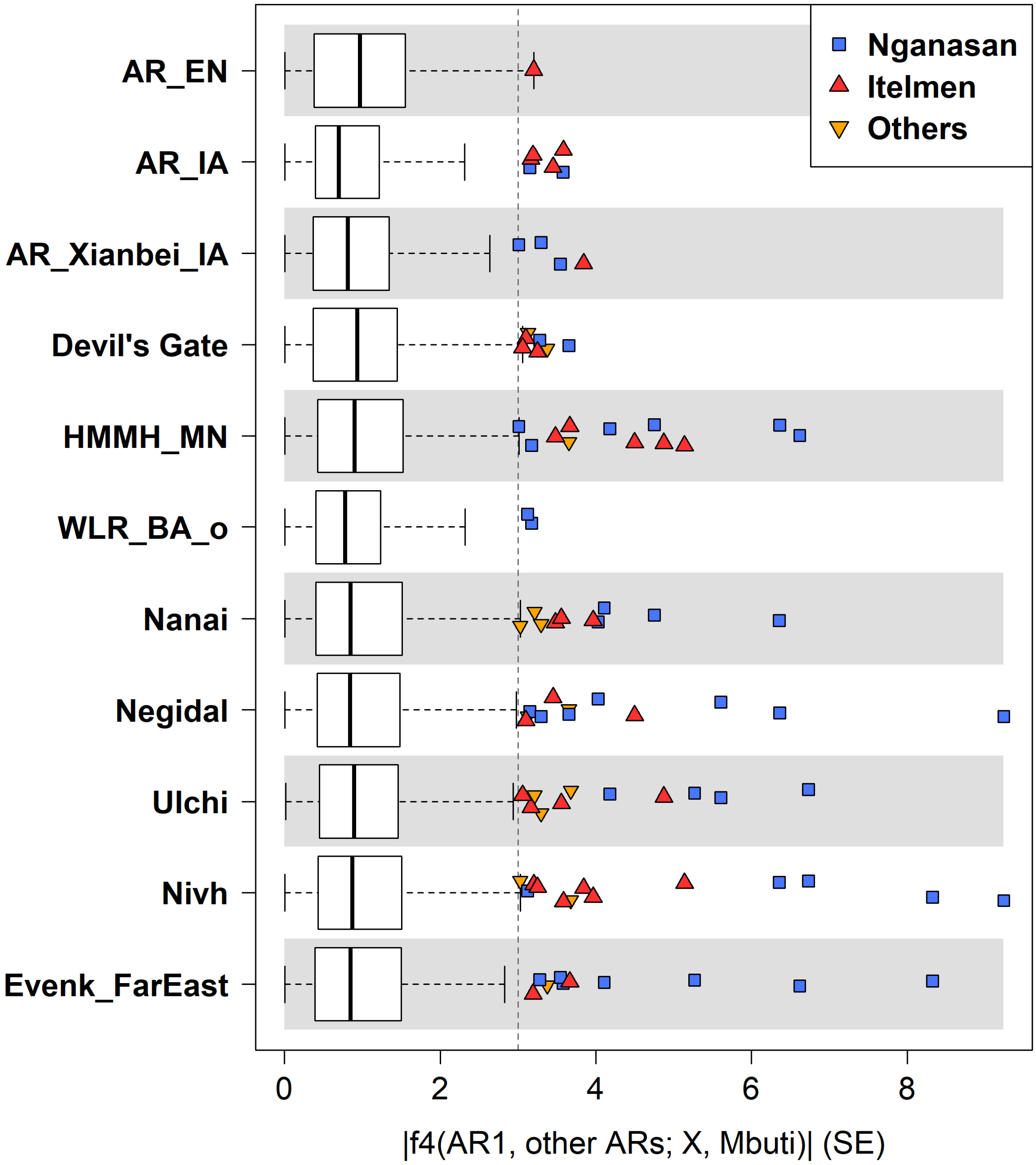

Supplementary Fig. 10. Distribution of f_4 symmetry statistics for the 6 ancient and 5 present-day AR populations. Each boxplot shows distribution of all 200 f_4(AR, other ARs; X, Mbuti) values involving each AR population. Most of the significant statistics (|Z| > 3 s.e.m.) involve Siberian populations Nganasan or Itelmen as an outgroup (X), while tests with other outgroups only mildly deviate from zero (max |Z| = 3.7). For each box plot, the box represents 25th to 75th percentile range, the thick vertical line inside the box represents the median, and the whiskers show a range of 1.5 times of the inter-quartile range (i.e. box width).

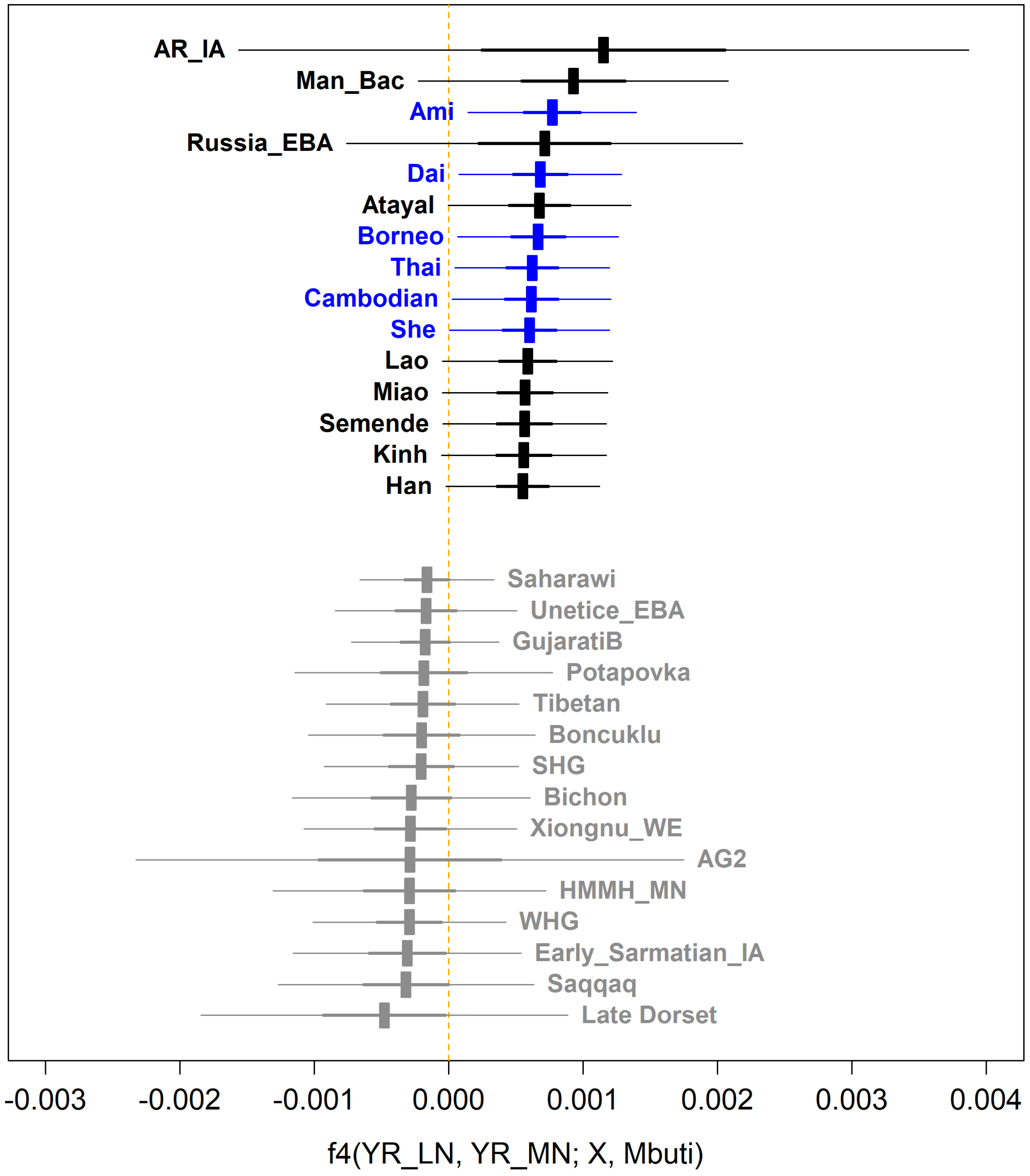

Supplementary Fig. 11. The genetic difference between Middle and Late Neolithic populations from the Central Plain. We present 15 most positive (upper side) and 15 most negative (lower side) f_4(YR_LN, YR_MN; X, Mbuti) statistics across 334 world-wide populations. Horizontal bars represent the point estimate ± 3 (thin) and ± 1 (thick) s.e.m, respectivley. s.e.m. are estimated using 5 cM block jackknifing. F_4 statistics deviating three s.e.m. or more from zero are marked in blue. Significant positive statistics are shown for many Southeast Asian populations (e.g. Ami and Dai), while no significant statistic is found in the negative end.

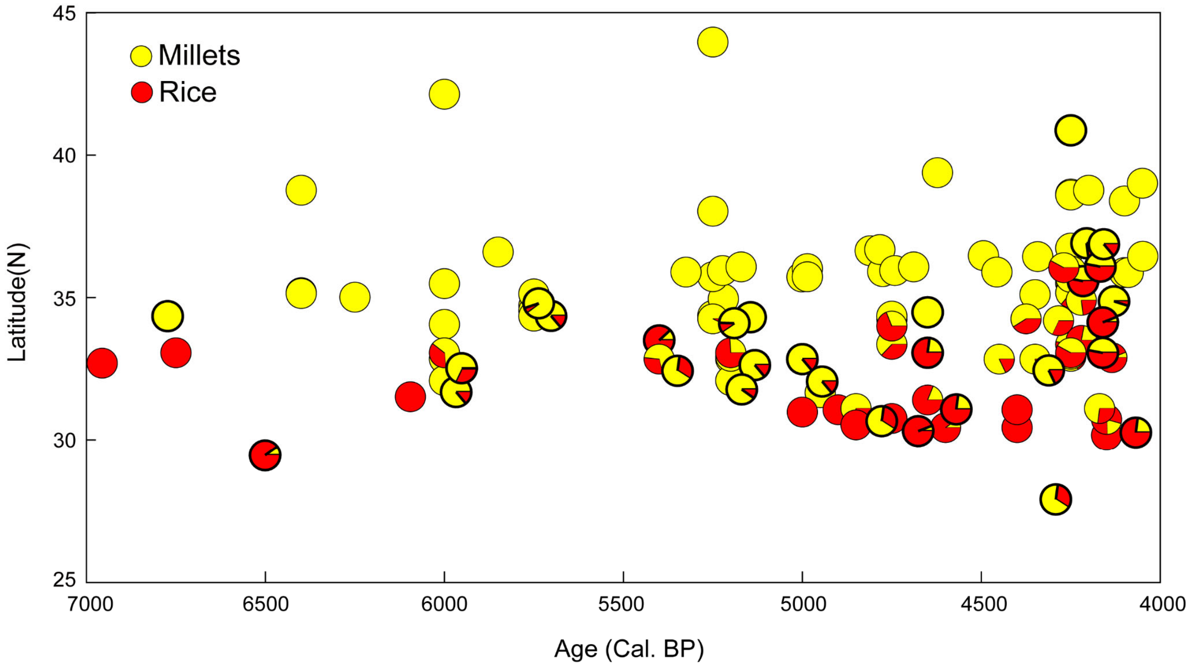

Supplementary Fig. 12. The latitudinal patterns of crop macro-fossils assemblage from sites dated between 7000-4000 BP in China. The circles with thick and thin lines represent archaeobotanical data from excavated sites and investigated sites respectively.

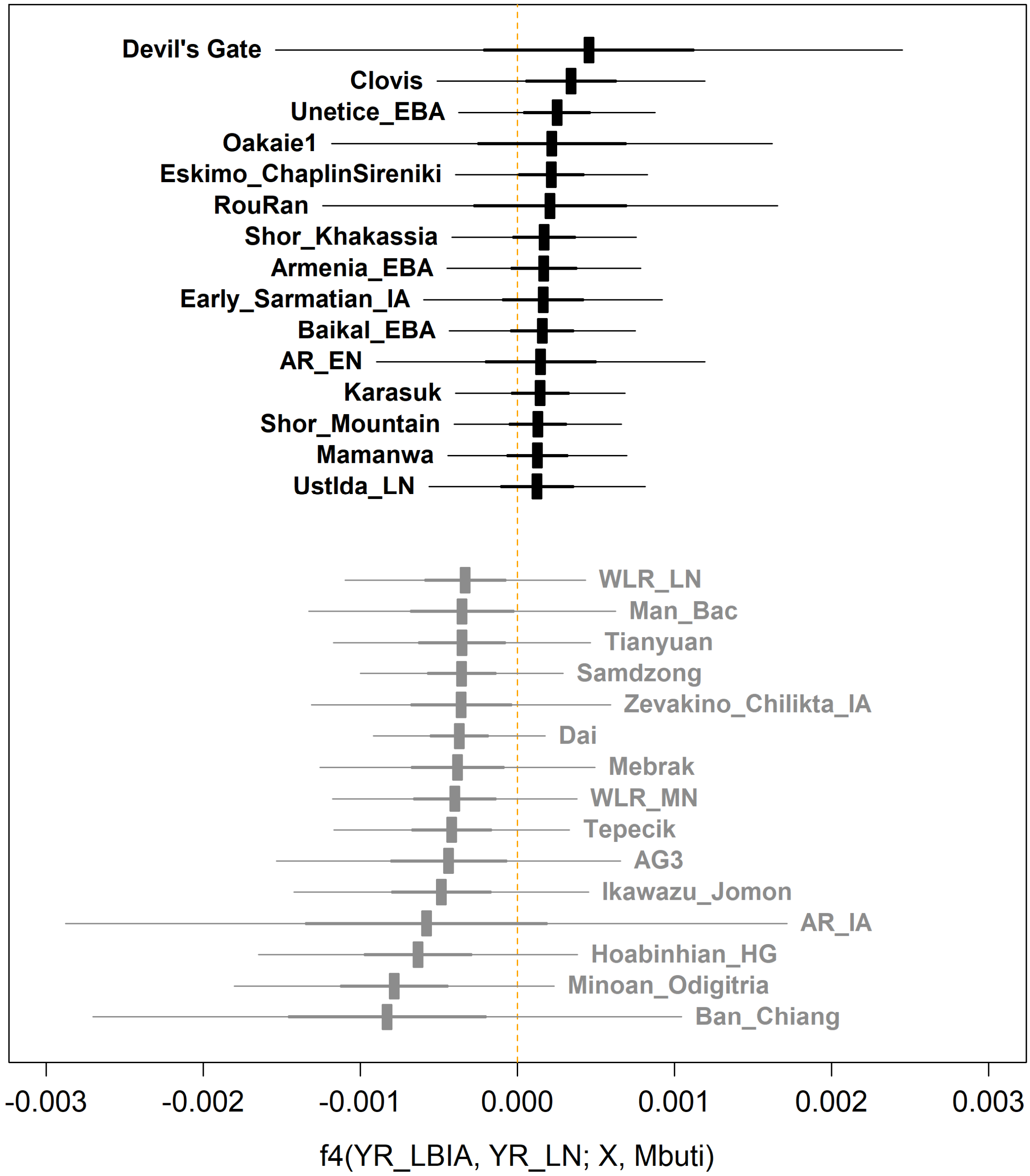

Supplementary Fig. 13. The genetic difference between Late Neolithic and Late Bronze/Iron Age populations from the Central Plain. We present 15 most positive (upper side) and 15 most negative (lower side) f_4(YR_LBIA, YR_LN; X, Mbuti) statistics across 334 world-wide populations. Horizontal bars represent the point estimate ± 3 (thin) and ± 1 (thick) s.e.m, respectively. s.e.m. are estimated using 5 cM block jackknifing. We find no significant difference between two populations, suggesting no substantial genetic change between two groups.

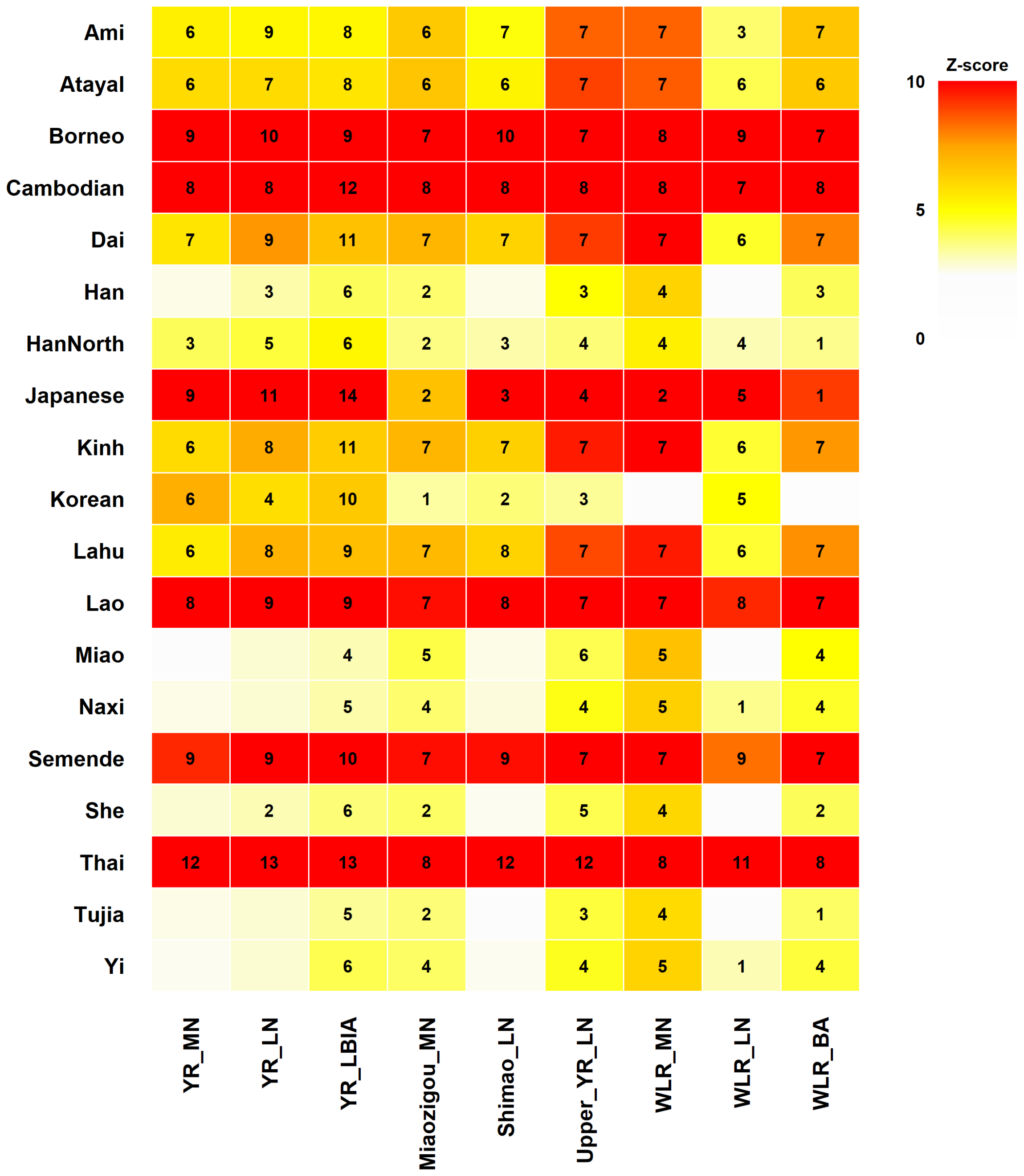

Supplementary Fig. 14. A comparison of genetic profile between ancient YR and WLR populations and present-day East Asians by f_4 symmetry statistics. We calculate f_4 symmetry statistics in the form of f_4 (Present-day East Asian, Ancient; X, Mbuti) for 20 representative world-wide populations outside of the YR region and Southeast Asia: Armenian, BedouinB, French, Itelmen, Mixe, Nanai, Nganasan, Nivh, Onge, Papuan, Sardinian, Sherpa, Tibetan, EHG, Ikawazu Jomon, LBK_EN, MA-1, Tianyuan, WHG, Yamnaya_Samara. Heatmap colors represent the biggest |Z| scores among 20 tests. Numbers inside each cell show the number of tests with |Z| ≥ 3 s.e.m. Z scores were calculated by dividing f_4 statistics by their corresponding 5 cM block jackknifing s.e.m.

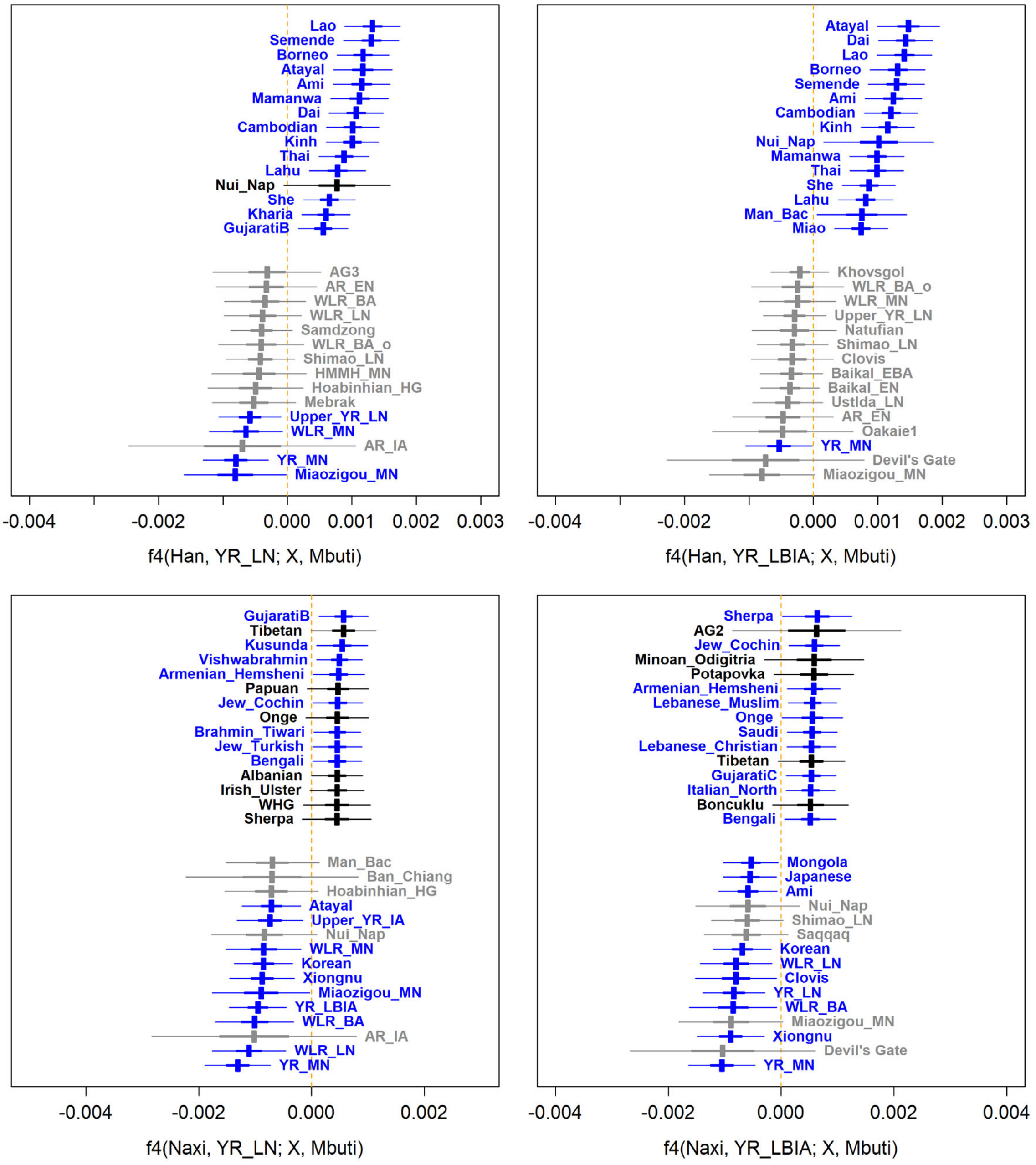

Supplementary Fig. 15. The genetic difference between ancient YR populations and present-day Sino-Tibetan-speaking ones. We present 15 most positive (upper side) and 15 most negative (lower side) f_4(present-day, ancient; X, Mbuti) statistics across 334 world-wide populations. Horizontal bars represent the point estimate ± 3 (thin) and ± 1 (thick) s.e.m, respectively. s.e.m. are estimated using 5 cM block jackknifing. F_4 statistics deviating three s.e.m. or more from zero are marked in blue color. We choose Late Neolithic (YR_LN; left panel) and Bronze/Iron Age (YR_LBIA; right panel) as ancients, and Han and Naxi as present-day representatives. Han Chinese shows strong extra affinity with southern Chinese and Southeast Asians, while Naxi show much reduced difference across world-wide populations.

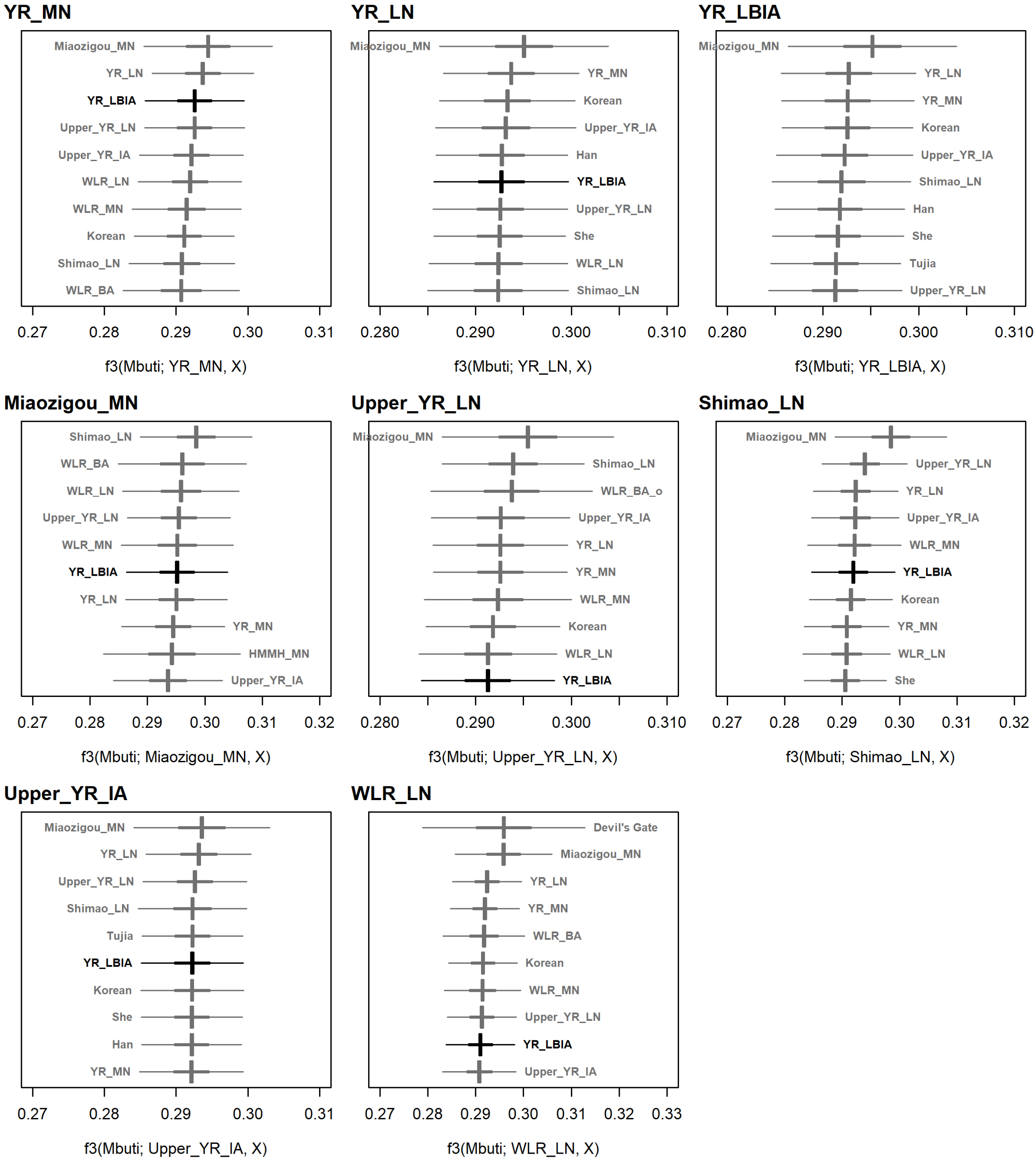

Supplementary Fig. 16. Close genetic relationship between ancient populations from the Central Plain and nearby regions. We present top 10 outgroup-f_3 signal for each population among 334 non-sub-Saharan African populations. Horizontal bars represent the point estimate ± 3 (thin) and ± 1 (thick) s.e.m, respectively. s.e.m. are estimated using 5 cM block jackknifing. Populations from the YR regions are marked in black, and those outside of the region are marked in grey.

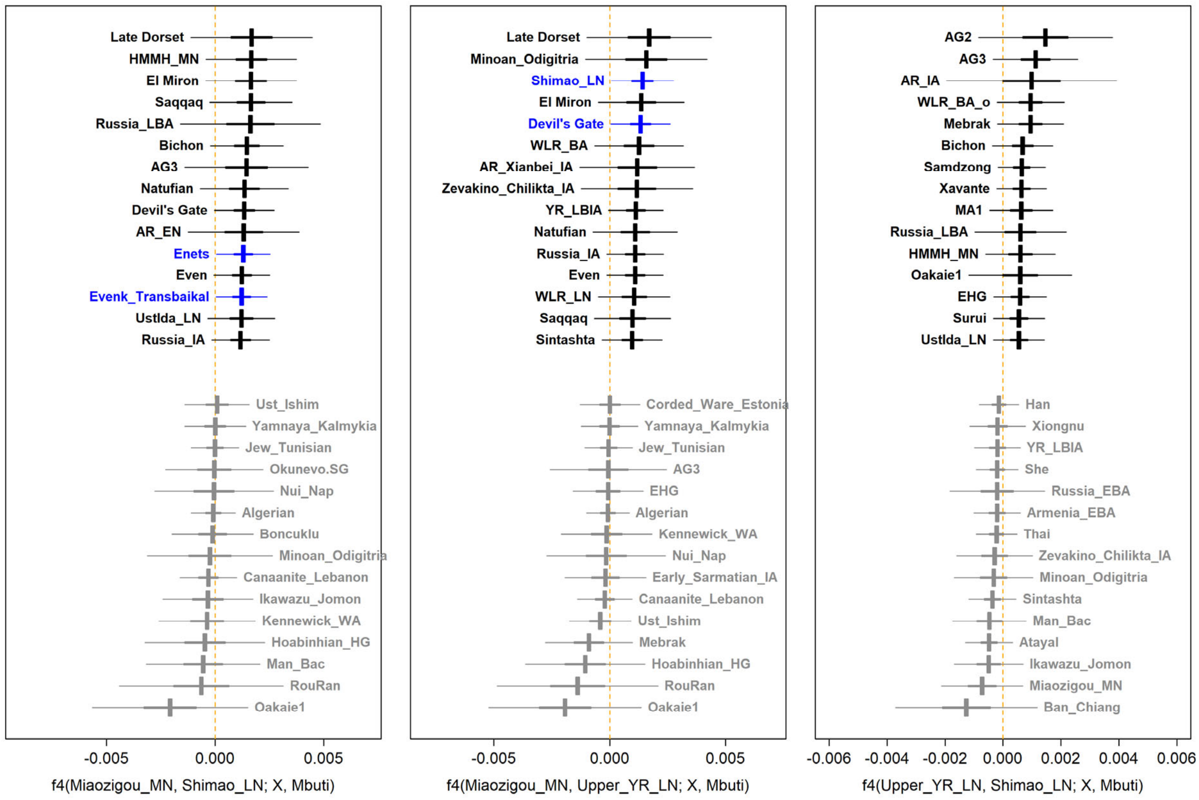

Supplementary Fig. 17. The genetic similarity between Miaozigou_MN, Shimao_LN, and Upper_YR_LN. We present 15 most positive (upper side) and 15 most negative (lower side) f_4 statistics of the form f_4(Target$_1$, Target$_2$; X, Mbuti) for each pair of three target populations (Miaozigou_MN, Shimao_LN, Upper_YR_LN) across 334 world-wide populations. Horizontal bars represent the point estimate ± 3 (thin) and ± 1 (thick) s.e.m, respectively. s.e.m are estimated using 5 cM block jackknifing. F_4 statistics deviating three s.e.m. or more from zero are marked in blue color. The most significant statistic across all three pairs is only 3.2 s.e.m., supporting that these three groups have a nearly identical genetic profile.

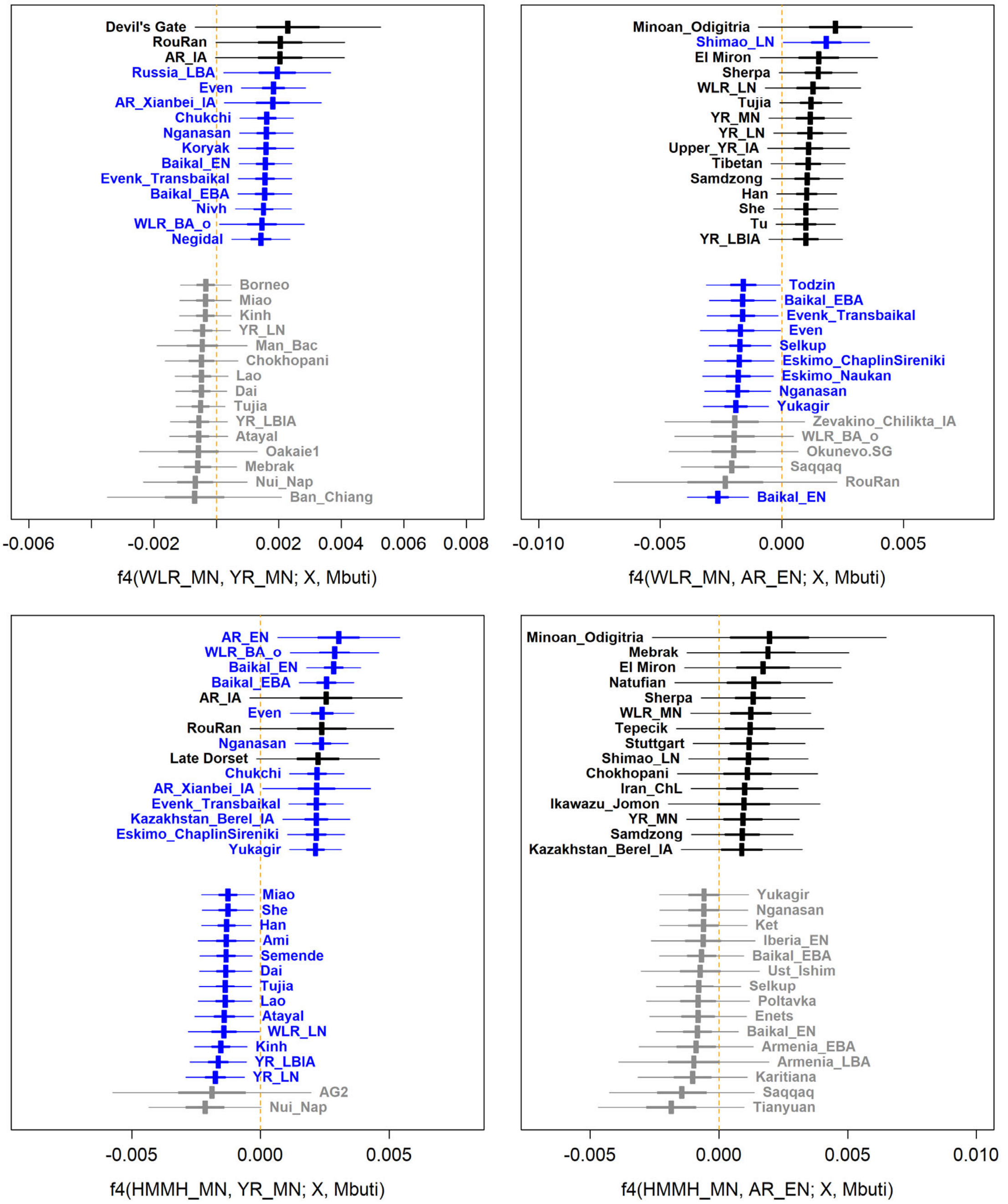

Supplementary Fig. 18. The genetic difference between Middle Neolithic WLR and YR/AR populations. We present 15 most positive (upper side) and 15 most negative (lower side) f_4(WLR_MN/HMMH_MN, YR_MN/AR_EN; X, Mbuti) statistics across 334 world-wide populations. Horizontal bars represent the point estimate ± 3 (thin) and ± 1 (thick) s.e.m, respectively. s.e.m are estimated using 5 cM block jackknifing. F_4 statistics deviating three s.e.m. or more from zero are marked in blue color. We compare two Middle Neolithic WLR groups (WLR_MN, HMMH_MN) with earlier or contemporaneous YR (WLR_MN; left panel) and AR (AR_EN; right panel) populations.

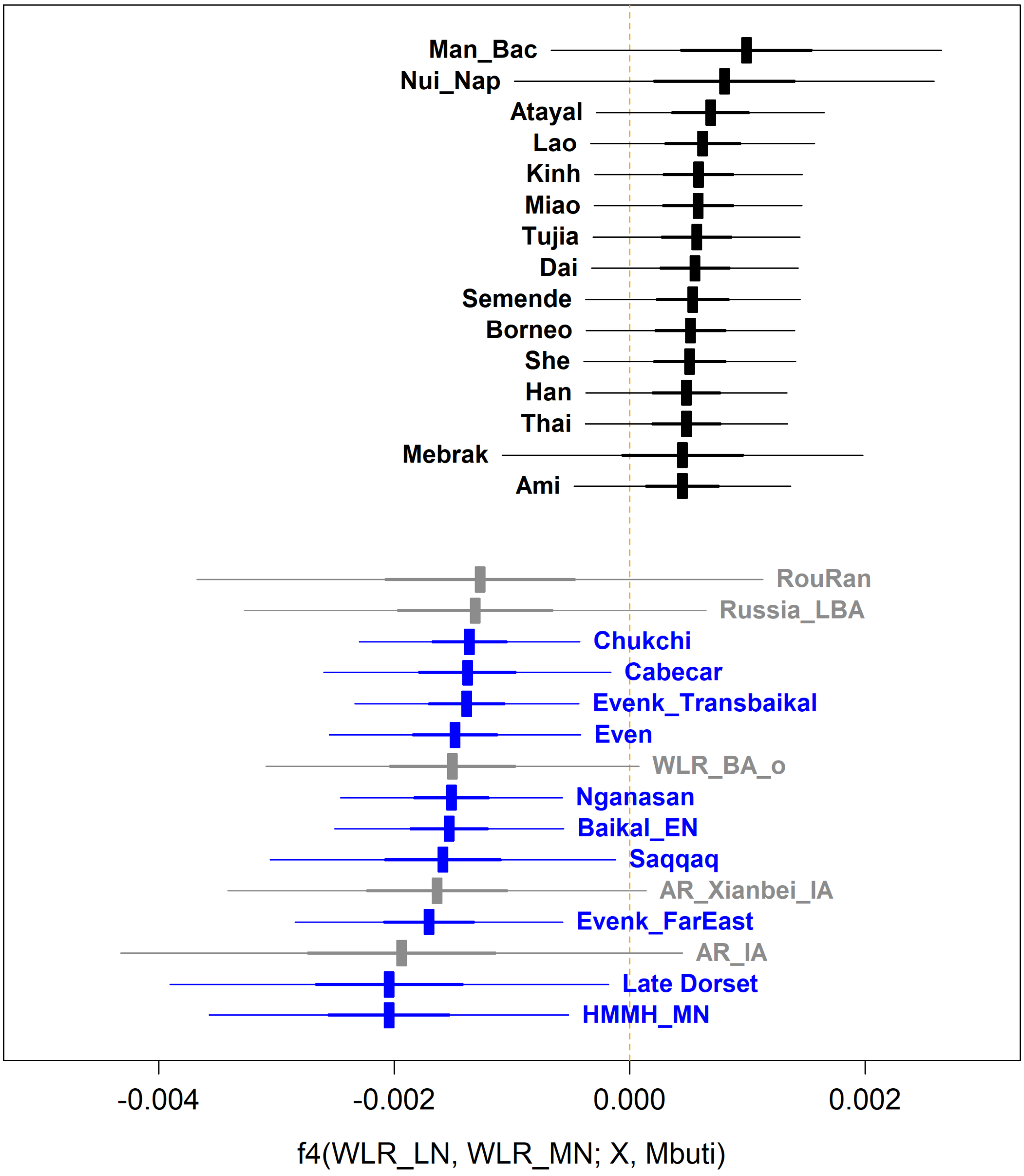

Supplementary Fig. 19. The genetic difference between Middle and Late Neolithic populations from the WLR region. We present 15 most positive (upper side) and 15 most negative (lower side) f_4(WLR_LN, WLR_MN; X, Mbuti) statistics across 334 world-wide populations. Horizontal bars represent the point estimate ± 3 (thin) and ± 1 (thick) s.e.m, respectively. s.e.m are estimated using 5 cM block jackknifing. F_4 statistics deviating three s.e.m. or more from zero are marked in blue. Southern Chinese and Southeast Asian populations are suggestively more closely related to Late Neolithic WLR individuals, while Middle Neolithic WLRs are significantly close to Siberian population.

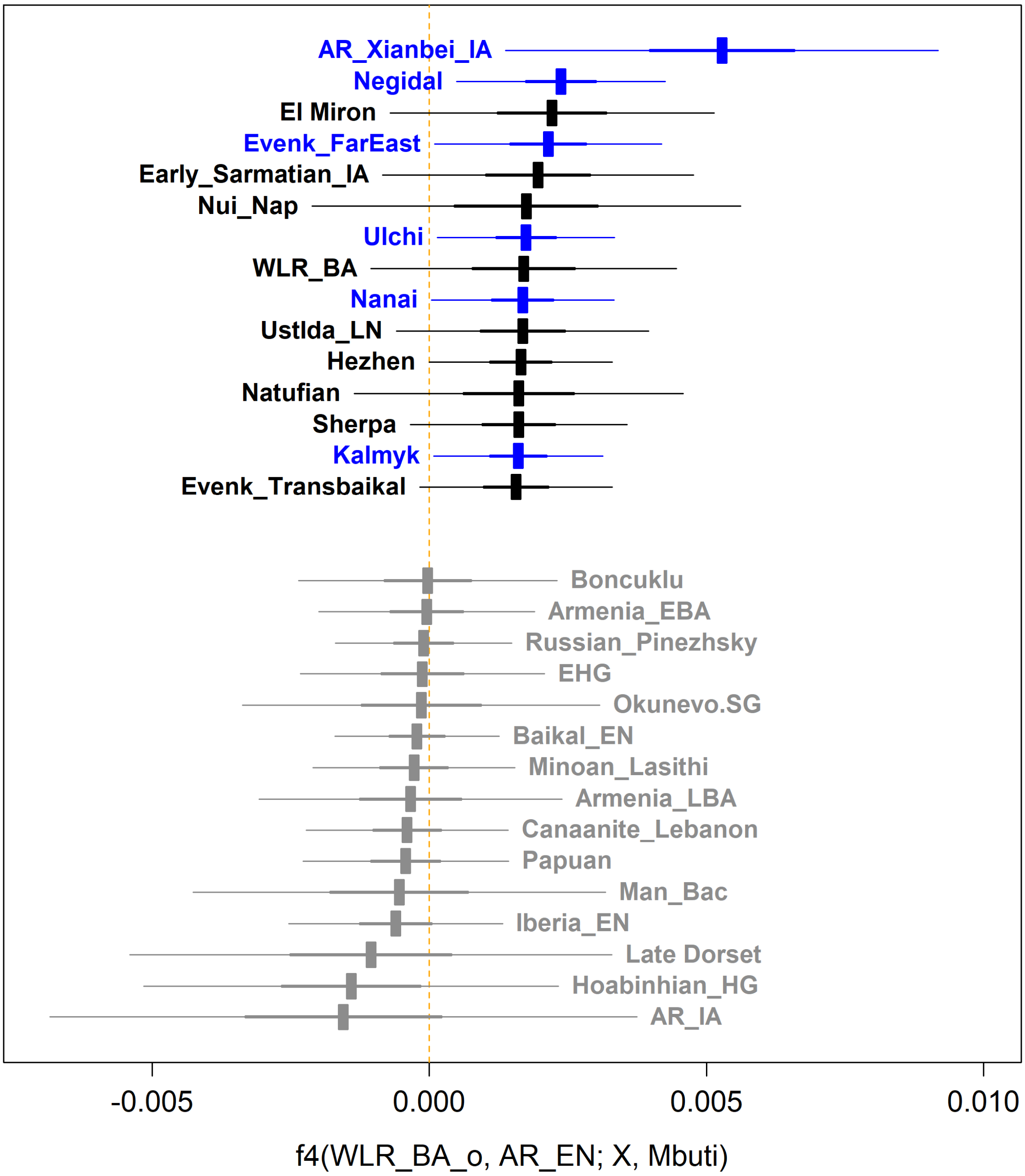

Supplementary Fig. 20. The genetic difference between Early Neolithic AR individuals (AR_EN) and Bronze Age WLR outlier (WLR_BA_o). We present 15 most positive (upper side) and 15 most negative (lower side) f_4(WLR_BA_o, AR_EN; X, Mbuti) statistics across 334 world-wide populations. Horizontal bars represent the point estimate ± 3 (thin) and ± 1 (thick) s.e.m, respectively. s.e.m are estimated using 5 cM block jackknifing. F_4 statistics deviating three s.e.m. or more from zero are marked in blue. Iron Age AR individuals (AR_Xianbei_IA) and several Tungusic-speaking populations show significant extra affinity with WLR_BA_o ($f_4 > 3$ s.e.m.).

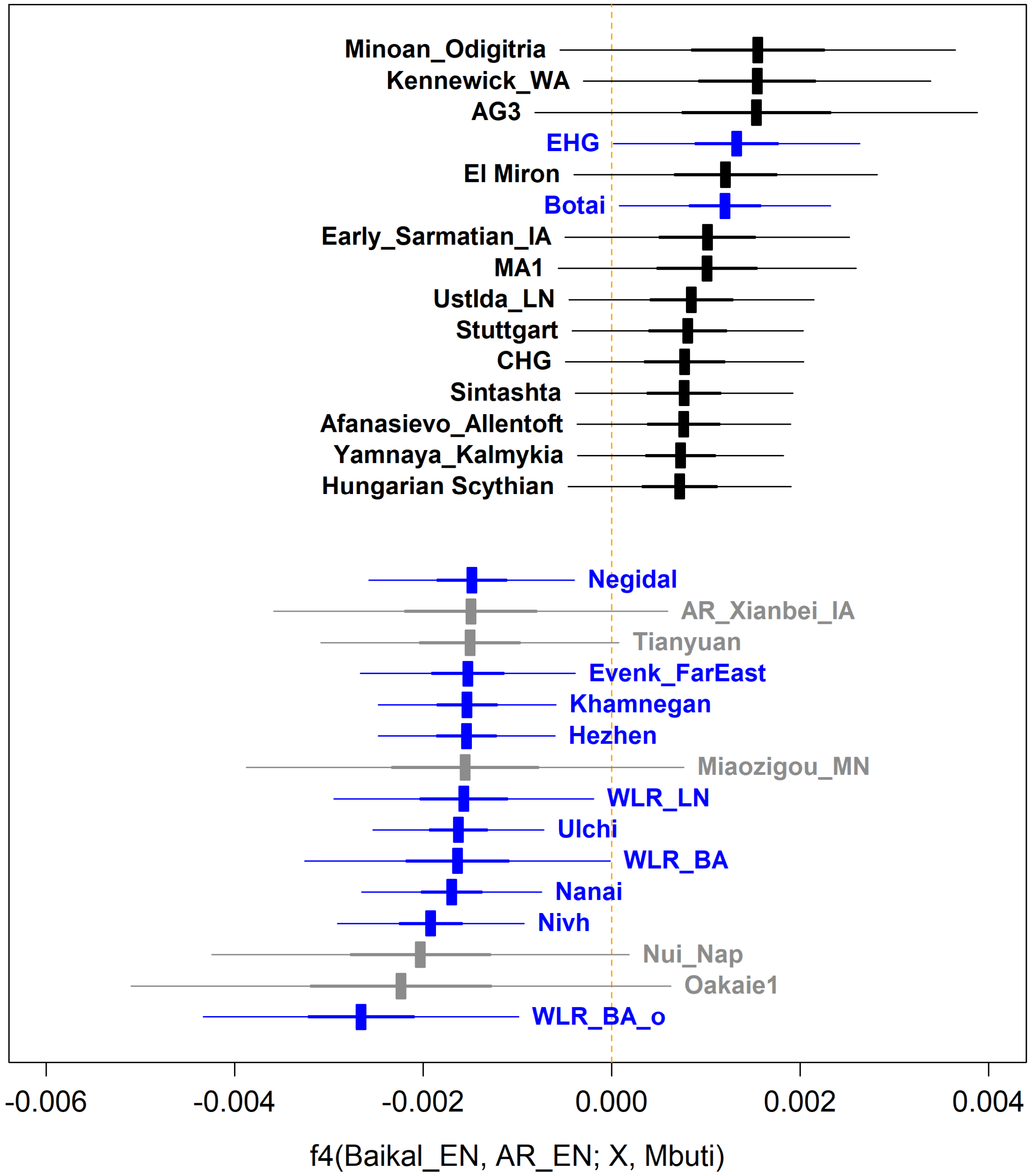

Supplementary Fig. 21. The genetic difference between early Neolithic populations in the Baikal and AR regions. We present 15 most positive (upper side) and 15 most negative (lower side) f_4(Baikal_EN, AR_EN; X, Mbuti) statistics across 334 world-wide populations. Horizontal bars represent the point estimate ± 3 (thin) and ± 1 (thick) s.e.m, respectively. s.e.m are estimated using 5 cM block jackknifing. F_4 statistics deviating three s.e.m. or more from zero are marked in blue color. Positive statistics with EHG and Botai suggest extra ANE affinity of Baikal Neolithics. Ancient and present-day AR populations show higher affinity to AR Neolithics.

Supplementary Table 1. Details of the ancient genomes generated in the present study. Calibrated ^{14}C dates are marked by bold face (2-sigma range), while dates in plain text are estimated from archaeological context.

Region	Group Label	ID	Date (cal. BCE)	# of human reads aligned	% human reads	# of HO SNPs covered	#of Illumina SNPs covered	Coverage
AR	AR_EN	WQM4	**5,480-5,320**	11,051,756	36.261	116,609	43,912	0.287
		ZLNR-2	**5,525-5,460**	7,022,968	49.340	107,971	42,901	0.239
	AR_IA	ZLNR-1	**66-222 CE**	2,054,390	13.628	32,677	12,711	0.068
	AR_Xianbei_IA	MGS-M6	50-250 CE	2,865,438	16.924	53,622	21,080	0.109
		MGS-M7L	50-250 CE	2,235,238	18.913	48,142	19,145	0.095
		MGS-M7R	50-250 CE	5,593,818	62.400	114,392	45,355	0.241
WLR	HMMH_MN	HMF32	**3,694-3,636**	65,486,765	6.118	359,994	146,645	1.431
	WLR_MN	BLSM27S	3,550-3,050	235,890,840	70.065	582,506	244,000	5.878
		BLSM41	3,550-3,050	1,838,689	12.872	21,635	8,269	0.048
		BLSM45	**3,338-3,098**	155,544,786	75.454	415,956	155,667	3.296
	WLR_LN	EDM124	**1,464-1,344**	8,151,932	7.321	87,827	35,324	0.212
		EDM139	2,050-1,550	37,939,588	17.840	249,441	96,474	0.848
		EDM176	2,050-1,550	87,790,708	35.817	448,372	177,451	2.398
	WLR_BA	91KLH11	1,050-350	25,560,526	27.523	188,999	72,306	0.574
		91KLH18	**901-825**	19,944,274	19.067	221,701	88,127	0.594
	WLR_BA_o	91KLM2	1,050-350	59,405,164	30.129	421,440	169,922	1.653
Inner Mongolia	Miaozigou_MN	MZGM10-1	3,550-3,050	16,198,775	41.929	122,615	48,107	0.348
		MZGM16	3,550-3,050	3,752,951	22.746	50,468	19,855	0.111
		MZGM25-2	3,550-3,050	2,509,204	29.257	28,251	11,225	0.065
Shaanxi	Shimao_LN	SM-M27	2,250-1,950	132,957,124	46.820	498,953	198,473	3.261
		SM-M6	**2,193-2,035**	167,999,003	91.053	564,509	235,541	4.478
		SM-M7X	2,250-1,950	1,059,834	8.703	11,690	4,481	0.026
Upper YR	Upper_YR_LN	JCKM1-1	**1,948-1,884**	37,616,736	13.491	423,172	173,664	1.487
		LJM14	2,050-1,850	57,968,442	49.818	382,925	155,512	1.483
		LJM25	2,050-1,850	2,113,727	27.587	25,766	9,991	0.056
		LJM2	2,050-1,850	40,839,055	91.409	366,494	149,354	1.217
		LJM3	**2,129-1,963**	64,478,132	68.229	500,659	207,290	2.316
		LJM4	2,050-1,850	1,452,790	15.583	19,948	8,215	0.043
		LJM5	2,050-1,850	40,530,938	85.421	328,564	131,191	1.084

(Continued on the next page)

Supplementary Table 1. *(Continued)*

Region	Group Label	ID	Date (cal. BCE)	# of human reads	% human reads	# of HO SNPs covered	#of Illumina SNPs covered	Coverage
Upper YR	Upper_YR_IA	DCZ-M17IV	50-150 CE	28,036,224	14.592	334,302	136,645	1.007
		DCZ-M21II	**68-128 CE**	33,442,988	15.949	374,610	150,333	1.206
		DCZ-M22IV	50-150 CE	64,452,209	52.099	508,063	206,313	2.439
		DCZ-M6	50-150 CE	1,018,878	8.790	17,180	6,764	0.034
YR	YR_MN	XW-M1R18	**4,225-3,987**	250,620,501	89.803	584,164	243,336	6.824
		WGM20	3,550-3,050	2,566,735	18.446	38,135	14,675	0.079
		WGM35	**3,354-3,106**	40,709,207	48.486	332,006	133,780	1.096
		WGH35-1	3,550-3,050	2,123,106	6.053	19,517	7,749	0.047
		WGM43	3,550-3,050	2,105,352	6.146	21,433	8,653	0.051
		WGM70	3,550-3,050	13,886,241	18.543	131,242	52,602	0.341
		WGM76S	3,550-3,050	12,443,112	33.283	120,061	47,893	0.307
		WGM94	3,550-3,050	29,743,568	27.629	242,720	95,824	0.737
	YR_LN	PLTM310	**2,275-2,045**	107,954,777	32.342	511,952	204,374	3.034
		PLTM311	**2,201-1,844**	129,393,625	42.403	553,427	226,303	3.928
		PLTM312	**2,135-1,939**	7,353,608	30.057	87,663	33,039	0.202
		PLTM313	**2,118-1,894**	135,973,650	46.230	564,232	232,001	4.289
		WD-WT1H16	**2,139-2,033**	241,230,765	94.527	586,085	243,595	7.533
		WD-WT5M2	2,050-1,850	98,510,733	71.788	467,235	181,307	2.544
		HJTM107	**2,118-1,950**	61,657,921	42.288	348,196	135,269	1.399
		HJTM109	**2,031-1,888**	47,403,034	20.530	269,374	104,887	0.986
	YR_LBIA	HJTM115	**365-207**	142,643,252	78.040	472,943	187,239	2.948
		HJTW13	**353-57**	1,129,897	12.494	15,325	5,967	0.033
		JXNTM2	1,550-1,050	82,569,610	90.743	461,399	186,042	2.144
		JXNTM23	**1,231-1,123**	62,609,646	86.854	412,484	166,549	1.634
		LGM41	250-50	66,060,872	93.180	427,068	170,904	1.761
		LGM79	**388-230**	73,193,890	76.465	447,868	180,322	1.947

Note: Among the samples that with available ^{14}C dating, 14 samples (91KLH18, WGM35, BLSM45, DCZ_M21II, EDM124, HJTM115, HMF32, JCKM1-1, JXNTM23, LGM79, LJM3, SM-M6, WD-WT1H16, XWM1R18) were dated by AMS at Lanzhou University in Lanzhou, China and all the rest were dated by the AMS at Beta Analytic, Miami, USA. The IntCal09 curve and the Libby half-life of 5,568 years were used in the calculation of all dates, with the calibration performed using Calib (v.6.0.1) or using the OxCal 4.2.3 program.

Supplementary Table 2. Contamination estimation and uniparental haplogroups of ancient individuals in this study. We present C to T misincorporation rate at the first base of the 5' end (5' C -> T), estimates for the nuclear contamination using ANGSD (X-contam.), mitochondrial contamination estimates from Schmutzi (MT contam.) and uniparental haplogroups. "n/a" represent failed estimates due to low coverage.

Region	Group Label	ID	Genetic sex	5' C -> T	X-contam. (± 1 s.e.m.)	MT_contam. (95% CI)	mtDNA Haplogroup	Y Haplogroup
AR	AR_EN	WQM4	F	0.134	n/a	0.01 (0.00-0.02)	C4a1a	n/a
		ZLNR-2	F	0.037	n/a	0.01 (0.00-0.02)	C5	n/a
	AR_IA	ZLNR-1	M	0.014	0.0630 ± 0.0635	0.01 (0.00-0.02)	N9a9	C2b1a
	AR_Xianbei_IA	MGS-M6	M	0.081	0.0269 ± 0.0271	0.01 (0.00-0.02)	C5a1	C2b1a1
		MGS-M7L	M	0.038	-0.0069 ± 0.0071	0.01 (0.00-0.02)	Z3a1	C2b1a1
		MGS-M7R	M	0.053	0.0044 ± 0.0061	0.01 (0.00-0.02)	C4a1a4a	C2b1a1
WLR	HMMH_MN	HMF32	F	0.242	n/a	0.02 (0.01-0.03)	D4j	n/a
	WLR_MN	BLSM27S	F	0.021	n/a	0.02 (0.01-0.03)	D5a3a1	n/a
		BLSM41	M	0.191	-0.0431 ± 0.0377	0.03 (0.01-0.05)	n/a	O2a
		BLSM45	F	0.145	n/a	0.01 (0.01-0.02)	D5a3a1	n/a
	WLR_LN	EDM124	M	0.171	0.0433 ± 0.0174	0.01 (0.01-0.02)	B5b1a	O2a1c
		EDM139	F	0.174	n/a	0.01 (0.01-0.02)	A22	n/a
		EDM176	M	0.207	0.0079 ± 0.0014	0.01 (0.01-0.02)	N9a1	n/a
	WLR_BA	91KLH11	F	0.124	n/a	0.01 (0.01-0.02)	D4m1	n/a
		91KLH18	M	0.054	0.0063 ± 0.0032	0.01 (0.01-0.02)	D4j14	NO
	WLR_BA_o	91KLM2	M	0.026	0.0049 ± 0.0011	0.01 (0.01-0.02)	B4c1a2	C2b1a1
Inner Mongolia	Miaozigou_MN	MZGM10-1	M	0.138	0.0138 ± 0.0091	0.02 (0.01-0.03)	A14	C2e2
		MZGM16	M	0.030	0.0345 ± 0.0266	0.02 (0.01-0.03)	C4a2a1	C2e2
		MZGM25-2	F	0.072	n/a	0.01 (0.01-0.02)	D4b2	n/a
Shaanxi	Shimao_LN	SM-M27	M	0.122	0.0044 ± 0.0009	0.01 (0.01-0.02)	G2a1	C2e1b2
		SM-M6	M	0.128	0.0091 ± 0.0008	0.02 (0.01-0.03)	D5a2a1b	O2a2b1a
		SM-M7X	F	0.187	n/a	0.02 (0.01-0.03)	M80'D	n/a
Upper YR	Upper_YR_LN	JCKM1-1	M	0.072	0.0058 ± 0.0013	0.01 (0.01-0.02)	G3a2	O2a2b1a
		LJM14	M	0.221	0.0177 ± 0.0023	0.01 (0.01-0.02)	A18	O2a2b1a
		LJM25	M	0.161	-0.0245 ± 0.0183	0.01 (0.01-0.02)	B4c1b2c2	n/a
		LJM2	F	0.066	n/a	0.01 (0.01-0.02)	F1g	n/a
		LJM3	M	0.097	0.0075 ± 0.0010	0.01 (0.01-0.02)	G1c1	O2a2b1a
		LJM4	F	0.098	n/a	0.04 (0.03-0.05)	F1a1a	n/a
		LJM5	M	0.169	0.0100 ± 0.0027	0.02 (0.01-0.03)	F1g	O2a2b1a

(Continued on the next page)

Supplementary Table 2. *(Continued)*

Region	Group Label	ID	Genetic sex	5' C -> T	X-contam. (± 1 s.e.m.)	MT_contam. (95% CI)	mtDNA Haplogroup	Y Haplogroup
Upper YR	Upper_YR_IA	DCZ-M17IV	F	0.057	n/a	0.01 (0.01-0.02)	D4b2b	n/a
		DCZ-M21II	M	0.076	0.0058 ± 0.0016	0.01 (0.01-0.02)	G2b1b	O2a2b1
		DCZ-M22IV	F	0.028	n/a	0.01 (0.01-0.02)	F1g	n/a
		DCZ-M6	F	0.064	n/a	0.01 (0.01-0.02)	Z3	n/a
YR	YR_MN	XW-M1R18	M	0.158	0.0106 ± 0.0007	0.01 (0.01-0.02)	D4g2a1	Q1a1a1
		WGM20	F	0.217	n/a	0.02 (0.00-0.04)	n/a	n/a
		WGM35	F	0.211	n/a	0.01 (0.01-0.02)	F1	n/a
		WGH35-1	F	0.033	n/a	n/a	n/a	n/a
		WGM43	M	0.056	-0.0474 ± 0.0291	0.01 (0.01-0.02)	n/a	n/a
		WGM70	F	0.213	n/a	0.01 (0.01-0.02)	M8a2	n/a
		WGM76S	F	0.027	n/a	0.03 (0.02-0.04)	C4a1a1	n/a
		WGM94	M	0.224	0.0222 ± 0.0048	0.01 (0.01-0.02)	B4d1	O1b1a2
	YR_LN	PLTM310	F	0.178	n/a	0.01 (0.01-0.02)	D4b1a	n/a
		PLTM311	M	0.171	0.0082 ± 0.0009	0.01 (0.01-0.02)	D4b1a	NO?
		PLTM312	M	0.186	0.0315 ± 0.0192	0.03 (0.02-0.04)	D4b1a	n/a
		PLTM313	F	0.206	n/a	0.01 (0.01-0.02)	F2h	n/a
		WD-WT1H16	M	0.164	0.0083 ± 0.0006	0.01 (0.01-0.02)	N9a2	O2a2b1
		WD-WT5M2	F	0.194	n/a	0.01 (0.01-0.02)	D5a2a	n/a
		HJTM107	M	0.187	0.0057 ± 0.0020	0.01 (0.01-0.02)	D4	C2e2
		HJTM109	M	0.214	0.0102 ± 0.0028	0.02 (0.01-0.03)	D4e1a	O2a1a2
	YR_LBIA	HJTM115	F	0.235	n/a	0.02 (0.01-0.03)	F4a2	n/a
		HJTW13	M	0.260	-0.0334 ± 0.0269	0.03 (0.02-0.04)	n/a	n/a
		JXNTM2	M	0.170	0.0110 ± 0.0015	0.01 (0.01-0.02)	A5b1b	O2a2b1a
		JXNTM23	F	0.177	n/a	0.02 (0.01-0.03)	C4a1a2	n/a
		LGM41	M	0.159	0.0112 ± 0.0018	0.01 (0.01-0.02)	M8a2b	O2a2b1a
		LGM79	F	0.148	n/a	0.01 (0.01-0.02)	B4d1'2'3	n/a

Supplementary Table 3. Cladal relationship of the ancient and present-day AR populations. (A) We test the cladality between six ancient (rows) and five present-day AR populations (columns) using the qpWave program. We also we carried out the same test by merging the above-mentioned ancient (aAR_merged) and present-day (pAR_merged) AR populations into a single group. Each cell provides the qpWave *p*-value (from χ^2 statistic for the likelihood ratio test) for the null hypothesis that two target populations (one ancient and one present-day) are symmetrically related to the chosen set of outgroups (Mbuti, Natufian, Onge, Iran_N, Villabruna, Anatolia_N, Mixe, Ami). Overall non-significant *p*-values ($p>0.05$) support that the ancient and present-day AR populations share a similar genetic profile. (B) We test the continuity between the same pairs of ancient and present-day AR populations in the strict sense: i.e. the ancient population does not have a private genetic drift not shared by the descendant present-day population ($t_2=0$ in the original study). Significant p-values ($p<0.05$; from χ^2 statistic for the likelihood ratio test) suggest the presence of ancient-specific drift ($t_2>0$). Non-significant results are presumably false negatives due to low coverage of the ancient individuals (AR_IA and AR_Xianbei_IA) or small sample size of the present-day individuals (Negidal, n=3; Evenk_FarEast, n=2).

A. QpWave *p*-value for the test of cladality

	Nanai	Negidal	Ulchi	Nivh	Evenk_FarEast	mAR_merged
AR_EN	0.108	0.334	0.372	0.283	0.426	0.279
AR_IA	0.334	0.687	0.377	0.289	0.579	0.399
AR_Xianbei_IA	0.033	0.156	0.181	0.155	0.043	0.166
Devil's Gate	0.338	0.320	0.454	0.508	0.316	0.418
HMMH_MN	0.071	0.037	0.124	0.291	0.106	0.111
WLR_BA_o	0.108	0.942	0.372	0.283	0.426	0.942
aAR_merged	0.058	0.033	0.141	0.316	0.193	0.091

B. *P*-value for the continuity test (ancients as the direct ancestor of the present-day populations)

	Nanai	Negidal	Ulchi	Nivh	Evenk_FarEast	mAR_merged
AR_EN	3.11×10^{-5}	0.999	5.83×10^{-5}	2.20×10^{-6}	0.999	1.33×10^{-5}
AR_IA	1.000	1.000	1.000	1.000	1.000	1.000
AR_Xianbei_IA	1.000	0.998	1.000	1.000	1.000	1.000
Devil's Gate	0.000	3.07×10^{-94}	0.000	0.000	8.69×10^{-27}	0.000
HMMH_MN	4.64×10^{-5}	1.000	1.35×10^{-20}	5.63×10^{-37}	7.71×10^{-5}	5.69×10^{-25}
WLR_BA_o	1.000	7.92×10^{-7}	1.48×10^{-18}	1.21×10^{-38}	1.000	3.85×10^{-17}
aAR_merged	0.000	1.000	1.07×10^{-296}	0.000	1.000	0.000

Supplementary Table 4. Admixture modeling of ancient populations from the periphery of the Central Plain or from the Middle Neolithic WLR region. For each target group, we provide two two-way admixture models: WLR_MN+AR_EN and WLR_LN+AR_EN. Both models provide adequate fit to all target groups (*P*-value ≥ 0.05). In all but one target (WLR_BA_o), at least one model shows a significant decrease in model fit with the exclusion of the minor ancestry component ($P_{\text{diff}} < 0.05$). s.e.m. are estimated by 5 cM block jackknifing. *P*-values (*P*-value and P_{diff}) are calculated by the likelihood ratio test of the associated χ^2 statistics.

Region	Target	*P*-value	P_{diff}	Coef_{YR}	Coef_{AR}	s.e.m.
**A. YR_MN as the YR source (AR source = AR_EN)**						
YR periphery	Miaozigou_MN	0.502	**0.027**	0.800	0.200	0.090
	Shimao_LN	0.278	**1.44×10^{-4}**	0.790	0.210	0.054
	Upper_YR_LN	0.943	**2.30×10^{-5}**	0.804	0.196	0.042
	Upper_YR_IA	0.771	0.324	0.947	0.053	0.053
WLR	WLR_MN	0.420	**3.74×10^{-12}**	0.602	0.398	0.057
	HMMH_MN	0.469	**0.009**	0.249	0.751	0.089
	WLR_LN	0.836	0.162	0.917	0.083	0.057
	WLR_BA	0.915	**0.008**	0.814	0.186	0.067
	WLR_BA_o	0.469	0.896	0.018	0.982	0.091
**B. WLR_LN as the YR source (AR source = AR_EN)**						
YR periphery	Miaozigou_MN	0.548	**0.028**	0.813	0.187	0.085
	Shimao_LN	0.808	**4.29×10^{-7}**	0.761	0.239	0.046
	Upper_YR_LN	0.370	**8.02×10^{-9}**	0.765	0.235	0.038
	Upper_YR_IA	0.438	**0.018**	0.884	0.116	0.048
WLR	WLR_MN	0.286	**1.11×10^{-16}**	0.572	0.428	0.054
	HMMH_MN	0.401	**0.013**	0.218	0.782	0.082
	WLR_LN	0.848	**0.026**	0.880	0.120	0.053
	WLR_BA	0.856	**4.16×10^{-5}**	0.750	0.250	0.059
	WLR_BA_o	0.484	0.638	0.042	0.958	0.084

Supplementary Table 5. Admixture modeling of Tibetans from Sherpas in Tibetan Plateau and ancient populations from the periphery of the Central Plain or from the Middle Neolithic WLR region. To increase the resolution of our analysis, we used a new '1240kIllumina' dataset, which more Sherpas (45) and Tibetans (30) are included. We provide two-way admixture models: All models provide adequate fit to the target groups (*P*-value ≥ 0.05). s.e.m. are estimated by 5 cM block jackknifing. *P*-values (*P*-value and P_{diff}) are calculated by the likelihood ratio test of the associated χ^2 statistics.

Ref_1	Ref_2	*P*-value	P_{diff}	$Coef_1$	$Coef_2$	s.e.m.
Sherpa	Upper_YR_LN	**0.735**	8.79×10^{-6}	0.757	0.243	0.059
	YR_MN	**0.335**	5.09×10^{-5}	0.814	0.186	0.049
	YR_LN	**0.400**	2.51×10^{-5}	0.860	0.140	0.032
	AR_EN	**0.544**	2.09×10^{-2}	0.945	0.055	0.023
	Naxi	**0.070**	4.90×10^{-4}	0.817	0.183	0.051
	Yi	**0.057**	6.63×10^{-4}	0.850	0.150	0.041
	Han	**0.062**	5.73×10^{-4}	0.907	0.093	0.025
	Miao	0.037	1.32×10^{-3}	0.916	0.084	0.025
	Dai	0.005	2.87×10^{-2}	0.954	0.046	0.020

Supplementary Table 6. Admixture modeling of major Sino-Tibetan populations from China by using Yangshao (YR_MN) as one potential source (Ref_1). We used the same dataset as described in Table S1. We provide two-way admixture models: Models provide adequate fit to the target groups with *P*-value ≥ 0.05. s.e.m. are estimated by 5 cM block jackknifing. *P*-values (*P*-value and P_{diff}) are calculated by the likelihood ratio test of the associated χ^2 statistics.

A. *P*-values for the two-way admixture models for the six target populations

Ref_1	Ref_2	Naxi	Yi	Lahu	Tujia	Han	Miao
Upper_YR_LN	Dai	**0.108**	**0.137**	**0.140**	1.39×10^{-6}	1.89×10^{-10}	1.49×10^{-3}
	She	2.94×10^{-2}	3.75×10^{-2}	2.92×10^{-9}	**0.627**	**0.498**	**0.933**
	Sherpa	1.08×10^{-4}	2.51×10^{-5}	1.86×10^{-10}	6.26×10^{-7}	1.25×10^{-6}	1.07×10^{-7}
YR_MN	Dai	**0.460**	**0.314**	**0.104**	4.06×10^{-4}	7.24×10^{-4}	3.24×10^{-2}
	She	**0.514**	**0.244**	4.13×10^{-9}	**0.407**	**0.613**	**0.911**
	Sherpa	**0.620**	**0.234**	3.25×10^{-6}	**0.090**	**0.257**	2.97×10^{-2}
YR_LN	Dai	1.09×10^{-5}	3.66×10^{-5}	3.40×10^{-3}	1.44×10^{-2}	3.82×10^{-3}	**0.223**
	She	1.09×10^{-3}	3.50×10^{-4}	4.47×10^{-6}	**0.406**	**0.261**	**0.711**
	Sherpa	**0.093**	3.54×10^{-3}	3.81×10^{-18}	2.04×10^{-5}	1.92×10^{-4}	3.13×10^{-7}
WLR_MN	Dai	1.46×10^{-3}	8.03×10^{-4}	9.18×10^{-3}	4.81×10^{-3}	9.52×10^{-3}	**0.375**
	She	1.86×10^{-6}	1.43×10^{-7}	2.55×10^{-7}	**0.350**	**0.781**	**0.787**
	Sherpa	1.17×10^{-9}	1.21×10^{-11}	3.45×10^{-15}	2.61×10^{-13}	1.45×10^{-12}	7.76×10^{-14}

B. Details of the admixture models with $P \geq 0.05$

Target	Ref_1	Ref_2	*P*-value	P_{diff}	$Coef_1$	$Coef_2$	s.e.m.
Naxi	Upper_YR_LN	Dai	0.108	1.03×10^{-5}	0.742	0.258	0.049
	YR_MN	Dai	0.460	1.000	1.003	-0.003	0.092
		She	0.514	0.496	1.094	-0.094	0.150
		Sherpa	0.620	0.240	0.856	0.144	0.110
	YR_LN	Sherpa	0.093	1.68×10^{-7}	0.643	0.357	0.055
Yi	Upper_YR_LN	Dai	0.137	1.90×10^{-8}	0.676	0.324	0.046
	YR_MN	Dai	0.314	0.312	0.912	0.088	0.083
		She	0.244	0.736	0.952	0.048	0.130
		Sherpa	0.234	0.929	1.019	-0.019	0.124
Lahu	Upper_YR_LN	Dai	0.140	1.37×10^{-7}	0.218	0.782	0.041
	YR_MN	Dai	0.104	1.84×10^{-7}	0.289	0.711	0.058
Tujia	Upper_YR_LN	She	0.627	0.026	0.109	0.891	0.047
	YR_MN	She	0.407	0.089	0.139	0.861	0.089
		Sherpa	0.090	3.57×10^{-10}	1.779	-0.779	0.215
		She	0.406	0.080	0.170	0.830	0.096
	WLR_MN	She	0.350	0.095	0.070	0.930	0.042
Han	Upper_YR_LN	She	0.498	4.56×10^{-6}	0.179	0.821	0.036
	YR_MN	She	0.613	5.48×10^{-6}	0.284	0.716	0.064
		Sherpa	0.257	2.51×10^{-8}	1.650	-0.650	0.191
		She	0.261	2.28×10^{-5}	0.343	0.657	0.071
	WLR_MN	She	0.781	2.90×10^{-6}	0.152	0.848	0.030
Miao	Upper_YR_LN	She	0.933	0.044	0.098	0.902	0.046
	YR_MN	She	0.911	0.065	0.143	0.857	0.077
		Dai	0.223	2.72×10^{-13}	0.523	0.477	0.051
		She	0.711	0.205	0.124	0.876	0.095
	WLR_MN	Dai	0.375	$< 10^{-15}$	0.300	0.700	0.032
		She	0.787	0.135	0.060	0.940	0.039

Supplementary Table 7. Admixture modeling of Late Neolithic or Bronze Age populations from the WLR region. We model Late Neolithic and Bronze Age WLR populations as a mixture of a YR source (YR_MN or YR_LN) and an AR/WLR source (AR_EN, HMMH_MN or WLR_MN). Additionally, we model WLR_BA as a mixture of Late Neolithic WLR (WLR_LN) and an AR/WLR source. All models provide adequate fit to all target groups (*P*-value ≥ 0.05). All models involving Upper_YR_LN show a significant decrease in model fit with the exclusion of the minor ancestry component ($P_{\text{diff}} < 0.05$). s.e.m. are estimated by 5 cM block jackknifing. WLR_LN derive most of its ancestry from a YR source, with maximum 26% contribution from the local source (WLR_MN). In contrast, WLR_BA shows much higher non-YR contribution in all six models. *P*-values (*P*-value and P_{diff}) are calculated by the likelihood ratio test of the associated χ^2 statistics.

Ref_1	Ref_2	*P*-value	P_{diff}	$Coef_1$	$Coef_2$	s.e.m.
**A. WLR_LN as the target**						
YR_MN	AR_EN	0.836	0.162	0.917	0.083	0.057
	HMMH_MN	0.666	0.561	0.957	0.043	0.072
	WLR_MN	0.261	0.410	0.891	0.109	0.139
YR_LN	AR_EN	0.848	**0.026**	0.880	0.120	0.053
	HMMH_MN	0.441	**0.016**	0.855	0.145	0.059
	WLR_MN	0.731	**0.014**	0.742	0.258	0.104
**B. WLR_BA as the target**						
YR_MN	AR_EN	0.915	**0.008**	0.814	0.186	0.067
	HMMH_MN	0.873	**6.30×10^{-4}**	0.718	0.282	0.078
	WLR_MN	0.830	**0.006**	0.480	0.520	0.155
YR_LN	AR_EN	0.856	**4.16×10^{-5}**	0.750	0.250	0.059
	HMMH_MN	0.839	**9.87×10^{-7}**	0.651	0.349	0.069
	WLR_MN	0.921	**0.004**	0.414	0.586	0.125
WLR_LN	AR_EN	0.929	0.167	0.880	0.120	0.082
	HMMH_MN	0.988	0.085	0.811	0.189	0.103
	WLR_MN	0.903	0.130	0.659	0.341	0.231
	WLR_BA_o	0.889	**0.004**	0.792	0.208	0.066

Supplementary Table 8. Pairwise mismatch rate estimates for the kinship among ancient Chinese individuals. We present the top 20 most related pairs of individuals estimated from pairwise mismatch analysis, we only present individuals with at least 3000 SNPs overlap.

ID1	ID2	nSNPs	nmismatch	pmismatch	Kinship
MGS-M7L	MGS-M7R	17171	3009	0.17524	1st degree
LJM3	LJM5	517223	93092	0.17998	1st degree
PLTM311	PLTM312	146348	28184	0.19258	1st degree?
PLTM310	PLTM312	137807	27853	0.20212	2nd degree
PLTM310	PLTM311	877478	186444	0.21248	2nd degree
MGS-M6	MGS-M7R	19285	4132	0.21426	2nd degree
BLSM27S	BLSM45	713675	153424	0.21498	2nd degree
BLSM41	BLSM45	28051	6048	0.21561	2nd degree
BLSM27S	BLSM41	38089	8233	0.21615	2nd degree
WQM4	ZLNR-2	39185	8799	0.22455	unrelated
BLSM41	HJTM109	19161	4324	0.22567	unrelated
91KLM2	MGS-M6	69994	15809	0.22586	unrelated
HJTM107	HJTW13	17374	3952	0.22747	unrelated
DCZ-M21II	HJTW13	17777	4044	0.22748	unrelated
91KLM2	MGS-M7L	63205	14384	0.22758	unrelated
WGM35	WGM43	23583	5377	0.22800	unrelated
MGS-M7L	ZLNR-2	16348	3732	0.22828	unrelated
MZGM25-2	WGM94	21896	4999	0.22831	unrelated
MGS-M6	ZLNR-2	18052	4124	0.22845	unrelated
MGS-M7R	WQM4	41029	9374	0.22847	unrelated

Supplementary Table 9. LcMLkin estimates for the kinship among ancient Chinese individuals. We present the top 20 most related pairs of individuals estimated from lcMLkin, we only present individuals with at least 3000 SNPs overlap.

Ind1	Ind2	k0_hat	k1_hat	k2_hat	pi_HAT	nrSNP	Kinship
LJM3	LJM5	0.236	0.727	0.036	0.400	258176	1st degree
MGS-M7L	MGS-M7R	0.421	0.200	0.379	0.479	8447	Parent/offspring
PLTM311	PLTM312	0.534	0.322	0.144	0.305	69930	1st degree?
PLTM310	PLTM312	0.65	0.263	0.087	0.219	64698	2nd degree
PLTM310	PLTM311	0.657	0.324	0.019	0.181	483178	2nd degree
MGS-M6	MGS-M7L	0.755	0.191	0.054	0.149	3913	2nd degree
MGS-M6	MGS-M7R	0.761	0.104	0.134	0.187	9275	2nd degree
BLSM27S	BLSM45	0.764	0.216	0.019	0.128	367334	2nd degree
MGS-M7L	ZLNR-2	0.833	0.120	0.047	0.107	7860	unrelated
BLSM41	BLSM45	0.855	0.124	0.021	0.083	11375	unrelated
91KLM2	MGS-M6	0.856	0.140	0.003	0.073	33711	unrelated
MGS-M6	ZLNR-2	0.872	0.119	0.009	0.068	8260	unrelated
BLSM27S	BLSM41	0.874	0.050	0.076	0.101	17556	unrelated
WQM4	ZLNR-2	0.876	0.113	0.011	0.067	16850	unrelated
91KLM2	MGS-M7R	0.878	0.114	0.007	0.064	74098	unrelated
91KLM2	ZLNR-1	0.878	0.119	0.003	0.062	20165	unrelated
LJM4	MGS-M7R	0.893	0.102	0.006	0.057	3445	unrelated
MGS-M7R	ZLNR-2	0.893	0.105	0.002	0.055	18590	unrelated
91KLM2	ZLNR-2	0.894	0.095	0.011	0.058	68199	unrelated
91KLM2	MGS-M7L	0.900	0.033	0.067	0.083	31097	unrelated

Supplementary Table 10. Admixture modeling of present-day Tungusic- and Nivkh-speaking populations and ancient populations from the nearby regions. We model the target populations as a mixture of AR_EN and MA-1, WLR_LN or Nganasans. Models with implausible (negative) coefficients are marked by grey color. Best models for each target (*P*-value ≥ 0.05 with ancestry coefficients between 0 and 1) are marked by grey shades. For models with nested *p*-value (P_{diff}) ≥ 0.05 (highlighted by bold face), we suggest that a single source model omitting the minor ancestry reference is sufficient. S.e.m. are estimated by 5 cM block jackknifing. *P*-values (*P*-value and P_{diff}) are calculated by the likelihood ratio test of the associated χ^2 statistics. For the AR populations and ancient Devil's Gate and RouRan, a single source model (AR_EN only) are mostly adequate. For the ancient Baikal populations and present-day Siberian Tungusic populations (Even and Evenk_Transbaikal), a model with additional ANE ancestry (modeled with MA-1) are the best. Finally, Tungusic-speaking populations further to the south from AR (Hezhen, Oroqen, Xibo) are modeled with substantial WLR_LN-related ancestry.

		Ref_2 = MA-1			Ref_2 = WLR_LN			Ref_2 = Nganasan		
**Group**	**Target**	***P*-value**	**P_{diff}**	**$Coef_2$ ± s.e.m.**	***P*-value**	**P_{diff}**	**$Coef_2$ ± s.e.m.**	***P*-value**	**P_{diff}**	**$Coef_2$ ± s.e.m.**
Ancients	Baikal_EN	0.611	5.64×10^{-6}	0.118 ± 0.024	0.040	6.99×10^{-4}	-0.485 ± 0.231	0.065	2.39×10^{-3}	0.692 ± 0.142
	Baikal_EBA	0.075	6.44×10^{-15}	0.223 ± 0.025	9.59×10^{-3}	$< 10^{-15}$	-1.411 ± 0.426	0.576	2.33×10^{-9}	1.685 ± 0.185
	Devil's Gate	0.184	**0.101**	0.166 ± 0.097	0.370	0.027	-3.609 ± 11.16	0.065	**0.661**	0.172 ± 0.506
	RouRan	0.349	**0.534**	0.051 ± 0.081	0.589	0.651	-0.256 ± 0.908	0.737	**0.386**	0.282 ± 0.318
North	Even	0.431	3.33×10^{-4}	0.099 ± 0.026	0.101	1.77×10^{-3}	-0.460 ± 0.216	0.227	2.52×10^{-3}	0.662 ± 0.131
	Evenk_Transbaikal	0.196	1.31×10^{-5}	0.116 ± 0.024	9.74×10^{-3}	1.71×10^{-4}	-0.590 ± 0.276	0.024	0.016	0.821 ± 0.095
AR	Evenk_FarEast	0.199	0.593	-0.016 ± 0.029	0.571	**0.114**	0.213 ± 0.126	0.329	0.295	-0.190 ± 0.216
	Negidal	0.243	0.539	-0.017 ± 0.028	0.187	**0.583**	0.080 ± 0.147	0.200	0.462	-0.136 ± 0.210
	Nanai	0.053	0.199	-0.032 ± 0.026	0.340	0.023	0.280 ± 0.107	0.134	0.068	-0.300 ± 0.218
	Ulchi	0.164	0.869	-0.006 ± 0.025	0.313	**0.267**	0.138 ± 0.118	0.230	0.615	-0.083 ± 0.179
	Nivh	0.203	0.575	-0.015 ± 0.026	0.183	**0.341**	0.125 ± 0.131	0.156	0.475	-0.124 ± 0.201
South	Hezhen	3.66×10^{-4}	0.037	-0.053 ± 0.027	0.284	5.47×10^{-4}	0.563 ± 0.113	0.026	1.05×10^{-4}	-0.696 ± 0.309
	Oroqen	1.79×10^{-3}	0.532	-0.017 ± 0.026	0.047	0.026	0.291 ± 0.122	0.022	0.045	-0.356 ± 0.249
	Xibo	6.10×10^{-6}	9.06×10^{-3}	-0.067 ± 0.028	0.209	0.032	0.728 ± 0.126	0.010	9.52×10^{-7}	-0.936 ± 0.374

Supplementary Table 11. Allele counts of variants with signatures of positive selection or with genotype-phenotype association. Cases with reads supporting the presence of derived alleles are marked by grey shades.

SNP	CHR	POS	REF	ALT	GENE	BLSM27S	BLSM45	DCZ-M22IV	EDM176	HJTM115	JXNTM2	LJM3	PLTM310	PLTM311	PLTM313	SM-SGDLM27	SM-SGDLM6	WD-WT1H16	WD-WT5M2	XW-M1R18
snp_2_136608574	2	136608574	C	T	*LCT*	9,0	3,0	2,0	0,0	3,0	3,0	3,0	1,0	1,0	1,0	3,0	3,0	11,0	1,0	4,0
snp_2_136608642	2	136608642	A	T		6,0	5,0	5,0	2,0	0,0	3,0	2,0	1,0	8,0	2,0	2,0	4,0	12,0	4,0	8,0
rs41525747	2	136608643	G	C		6,0	5,0	5,0	2,0	0,0	3,0	3,0	2,0	8,0	2,0	2,0	4,0	12,0	3,0	8,0
rs4988236	2	136608644	G	A		6,0	2,0	5,0	1,0	0,0	1,0	2,0	1,0	5,0	2,0	1,0	3,0	10,0	2,0	4,0
rs4988235	2	136608646	G	A		5,0	1,0	4,0	1,0	0,0	1,0	2,0	1,0	5,0	2,0	1,0	2,0	11,0	2,0	4,0
rs41456145	2	136608649	A	G		5,0	3,0	3,0	2,0	0,0	2,0	2,0	1,0	6,0	2,0	0,0	3,0	10,0	3,0	5,0
rs41380347	2	136608651	A	C		8,0	5,0	5,0	3,0	1,0	3,0	4,0	2,0	7,0	3,0	2,0	4,0	11,0	5,0	5,0
rs145946881	2	136608746	C	G		5,0	3,0	6,0	2,0	1,0	0,0	6,0	3,0	4,0	5,0	1,0	6,0	9,0	0,0	6,0
rs3827760	2	109513601	A	G	*EDAR*	0,3	0,1	0,3	0,2	0,2	0,1	0,2	0,2	1,4	0,4	0,8	0,1	0,10	0,1	0,6
rs17822931	16	48258198	C	T	*ABCC11*	0,4	0,5	0,1	0,0	0,1	0,1	0,2	0,4	0,4	0,4	0,3	0,0	0,5	0,1	0,0
rs1229984	4	100239319	T	C	*ADH1B*	0,2	0,0	0,2	0,2	0,0	0,2	0,4	0,3	1,1	0,4	0,1	0,1	2,0	0,2	0,4
rs1800414	15	28197037	T	C	*OCA2*	3,0	0,0	2,2	0,2	1,2	3,0	3,0	1,1	2,0	2,0	2,0	6,0	10,0	1,0	6,0
rs2228479	16	89985940	G	A	*MC1R*	5,1	24,0	3,0	6,0	3,3	0,0	1,0	6,0	5,0	8,0	4,0	2,1	19,0	6,0	1,6
rs12097901	1	231557255	C	G	*EGLN1*	2,2	2,0	1,0	8,0	1,0	1,0	1,1	0,4	0,4	7,0	4,4	0,0	0,6	0,0	8,0
rs186996510	1	231557623	G	C		2,1	6,0	1,0	3,0	6,0	1,0	0,0	2,0	2,0	3,0	4,0	2,1	3,0	2,0	3,0

(Continue in the next page)

Supplementary Table 11. (*continued*)

SNP	CHR	POS	REF	ALT	GENE	BLSM27S	BLSM45	DCZ-M22IV	EDM176	HJTM115	JXNTM2	LJM3	PLTM310	PLTM311	PLTM313	SM-SGDLM27	SM-SGDLM6	WD-WT1H16	WD-WT5M2	XW-M1R18
rs115321619	2	46567916	G	A	*EPAS1*	5,0	4,0	2,0	1,0	2,0	1,0	0,0	9,0	6,0	7,0	5,0	4,0	9,0	4,0	14,0
rs73926263	2	46568680	A	G		2,0	2,0	2,0	0,0	2,0	3,0	2,0	1,0	3,0	2,0	0,0	3,0	9,0	5,0	2,0
rs73926264	2	46569017	A	G		10,0	1,0	3,0	0,0	2,0	1,0	1,0	1,0	5,0	5,0	1,0	5,0	9,0	4,0	8,0
rs73926265	2	46569770	G	A		5,0	3,0	0,0	1,0	4,0	0,0	1,0	1,0	1,0	2,0	2,0	2,0	3,0	4,0	3,0
rs55981512	2	46570342	G	A		4,0	3,0	3,0	1,0	2,0	2,0	2,0	1,0	4,0	1,0	1,0	2,0	9,0	2,0	7,0
rs149306391	2	46571017	C	G		2,0	1,0	0,0	2,0	3,0	1,0	2,0	2,0	2,0	4,0	4,0	5,0	6,0	3,0	7,0
2:46571435	2	46571435	G	C		4,0	8,0	0,0	4,0	7,0	0,0	4,0	4,0	8,0	6,0	4,0	6,0	18,0	3,0	9,0
rs188801636	2	46577251	T	C		6,0	0,0	0,0	0,0	0,0	0,0	3,0	1,0	0,0	1,0	0,0	4,0	0,0	0,0	0,0
2:46579689	2	46579689	A	G		3,0	4,0	1,0	2,0	2,0	1,0	2,0	5,0	4,0	3,0	5,0	2,0	6,0	2,0	7,0
rs189807021	2	46583581	G	A		0,0	6,0	4,0	0,0	1,0	0,0	1,0	1,0	2,0	1,0	3,0	2,0	4,0	1,0	4,0
2:46584859	2	46584859	A	G		7,0	1,0	4,0	1,0	1,0	1,0	2,0	1,0	1,0	1,0	2,0	2,0	7,0	3,0	3,0
rs150877473	2	46588019	C	G		5,0	6,0	3,0	1,0	1,0	1,0	2,0	5,0	5,0	7,0	11,0	4,0	12,0	6,0	11,0
rs142826801	2	46588331	G	C		4,0	9,0	2,0	3,0	2,0	0,0	1,0	1,0	2,0	1,0	3,0	2,0	7,0	4,0	2,0
rs74898705	2	46589032	C	T		1,0	3,0	3,0	0,0	2,0	1,0	1,0	2,0	3,0	2,0	5,0	0,0	10,0	2,0	8,2
rs141366568	2	46594122	A	G		6,0	0,0	2,0	4,0	3,0	1,0	2,0	2,0	3,0	4,0	3,0	4,0	6,0	2,0	5,0
rs116062164	2	46597756	A	C		7,0	5,0	6,0	4,0	1,0	5,0	5,0	5,0	5,0	6,0	4,0	8,0	7,0	2,0	12,0
rs141426873	2	46598025	C	G		0,0	4,0	1,0	1,0	2,0	0,0	1,0	1,0	2,0	4,0	4,0	3,0	8,0	3,0	5,0
rs116611511	2	46600030	A	G		3,0	2,0	3,0	1,0	2,0	1,0	1,0	7,0	6,0	2,0	2,0	8,0	7,0	0,0	5,0
2:46600358	2	46600358	A	G		7,0	1,0	2,0	2,0	0,0	0,0	0,0	3,0	2,0	3,0	1,0	1,0	6,0	0,0	4,0

Supplementary References

1. Lu, H. *et al*. Earliest domestication of common millet (*Panicum miliaceum*) in East Asia extended to 10,000 years ago. *Proc. Natl. Acad. Sci. U. S. A*. **106**, 7367–7372 (2009).
2. Yang X. *et al*. Early millet use in northern China. *Proc. Natl. Acad. Sci.* **109**, 3726–3730 (2012).
3. Liu, L. The archaeology of China: from the late Paleolithic to the early Bronze Age. (Cambridge Univ. Press, Cambridge, 2012).
4. Kato, H. Neolithic culture in Amurland: The formation process of a prehistoric complex hunter-gatherer society. Ch. 1, (Graduate School of Letters, Hokkaido University, 2006).
5. Duggan, A. T. *et al*. Investigating the prehistory of Tungusic peoples of Siberia and the Amur-Ussuri region with complete mtDNA genome sequences and Y-chromosomal markers. *PLoS One* **8**, e83570 (2013).
6. Chard, C. S. Northeast Asia in prehistory. (University of Wisconsin Press, Madison, 1974).
7. Testart, A. *et al*. The significance of food storage among hunter-gatherers: Residence patterns, population densities, and social inequalities. *Curr. Anthropol*. **23**, 523–537 (1982).
8. Okladnikov, A. P., The archaeological studies in 1935 on the Amur River. *Sov. Archaeol.* **1**, 275–277 (1936).
9. Han, M. A study on the settlements and environment of prehistoric times in the West Liao River Valley (In Chinese). *Kaogu Xuebao* **4**, 1–5 (2015).
10. Sun, Y., A comparative study of prehistoric subsistence on west Liaohe upstream region and Central Plain of China (In Chinese). *Neimenggu Shifan Daxue Xuebao* **38**, 269–275 (2015).
11. Tian, G. Charactering ancient cultural development in Xiliaohe area in prehistoric northeast China and its division of history (In Chinese). *Laioning Shifan Daxue Xuebao* **27**, 111–114 (2004).
12. Huang, Y., Zhang, J. & Cui, J. The flourishing of modern agriculture (In Chinese). (Yanbian Daxue Press, Yanbian, 2017).
13. Zhao, Z. New archaeobotanical data for the study of the origins of agriculture in China. *Curr. Anthropol.* **52**, S295–S306 (2011).
14. Zhao, Z. The floatation results at Xinglonggou site and its implications for understanding the dry-land farming in north China (In Chinese). *Dongya Guwu* **1**, 188–189 (2004).
15. Yan, W. The beginning and spread of agriculture in Northeast Asia (In Chinese). *Nongye Kaogu* **3**, 11-18 (1993).
16. Liu, L., Duncan, N. A., Chen, X., Liu, G. & Zhao, H. Plant domestication, cultivation, and foraging by the first farmers in early Neolithic Northeast China: Evidence from microbotanical remains. *The Holocene* **25**, 1965–1978 (2015).
17. Zhang, X., Liu, G., Wang, M. & Lv, P. Stable isotopic analysis of human remains from Xinglonggou site (In Chinese). *Nanfang Wenwu* **4**, 185–195 (2017).

18. Tao, D., Wu, Y., Guo, Z., Hill, D. V. & Wang, C. Starch grain analysis for ground stone tools from Neolithic Baiyinchanghan site: implications for their function in Northeast China. *J. Archaeol. Sci.* **38**, 3577–3583 (2011).
19. Wu, W., Xin, Y., Wang, H., Jin, G. & Yang, X. Subsistence economy at Chahai site in Fuxin, Liaoning Province (In Chinese). *Kaogu Yu Wenwu* **6**, 110–113 (2013).
20. Chifeng International Collaborative Archaeological Research Project. Settlement patterns in the Chifeng region. (Univ. Pittsburgh Center for Comparative Archaeology, Pittsburg, 2011).
21. Xi, Y. & Teng, H. Review and comments on Zhaobaogou Culture (In Chinese). *Chifeng Xueyuan Xuebao* **32**, 11–14 (2011).
22. Wang, X. Microwear analysis of microliths unearthed from the Xinglongwa and Zhaobaogou sites (In Chinese). *Xibu Kaogu* **1**, (2006).
23. Peterson, C. E. Hongshan regional organization in the Upper Daling River. (Univ. Pittsburgh Center for Comparative Archaeology, Pittsburg, 2014).
24. Xue, L. & Zhang, G. Agricultural tools initially used in the Hongshan period (In Chinese). *Xueyuan Xuebao* **32**, 6–7 (2011).
25. Zhao, Z. & Sun, Y. Study on the plant remains unearthed at Weijiawopu, a Hongshan site (In Chinese). *Nongye Kaogu* **3**, 1–5 (2013).
26. Liu, X., Wang, T., Wei, D. & Hu, Y. Preliminary exploitation on human lifestyle during Xiaoheyan culture period: A case study of the Jiangjialiang site (In Chinese). *Renleixue Xuebao* **36**, 280–288 (2016).
27. Gao, M. & Li, G. A few issues on the Lower Xiajiadian Culture (In Chinese). *Liaoning Daxue Xuebao* **5**, 50–56 (2014).
28. Xiong, Z. *et al.* Excavation report of Blashan site in Chaoyang, Liaoning (In Chinese). *Kaogu* **2**, (2017).
29. Archaeological research institute of Inner Mongolia. The excavation of the Neolithic site at Haminmangha in Horqin left-middle Banner, Inner Mongolia in 2011 (In Chinese). *Zhongguo Kaogu.* **14**, 10–17 (2015).
30. Zhang, Z. & International Water Management Institute. Yellow River comprehensive assessment: basin features and issues : collaborative research between International Water Management Institute (IWMI) and Yellow River Conservancy Commission (YRCC). (International Water Management Institute, 2003).
31. Wang, C. *et al.* The spatial pattern of farming and factors influencing it during the Peiligang culture period in the middle Yellow River valley, China. *Sci. Bull.* **62**, 1565–1568 (2017).
32. Lu, T. L. D. The transition from foraging to farming and the origin of agriculture in China. (British Archaeological Reports Publishing, Oxford, 1999).
33. Zhang, J. *et al.* Early mixed farming of millet and rice 7800 years ago in the middle Yellow River region, China. *PLoS One* **7**, e52146 (2012).
34. Pechenkina, E. A., Benfer, R. A. & Zhijun, W. Diet and health changes at the end of the Chinese neolithic: the Yangshao/Longshan transition in Shaanxi province. *Am. J. Phys. Anthropol.* **117**, 15–36 (2002).

35. Zhou, Y. & Gu, W. Ancient human jaw osteomyelitis in Chinese Yangshao period: a case report (In Chinese). *Huaxi Kouqiang Yixue Zazhi* **35**, 663–664 (2017).
36. Li, M. *et al.* Archeology of the Lu City: Place memory and urban foundation in early China. *Archaeol. Res. Asia* **14**, 151–160 (2018).
37. Qiu, P. & Wang, C. Initial study on carbon fiber in the black pottery of the Dawenkou and Longshan cultures (In Chinese). *Stud. Hist. Nat. Sci.* **1**, 15-30 (2001).
38. Huang, T. M. Liangzhu – a late Neolithic jade-yielding culture in southeastern coastal China. *Antiquity* **66**, 75–83 (1992).
39. Lu, H. *et al.* Millet noodles in Late Neolithic China. *Nature* **437**, 967-968 (2005).
40. Zhimin, A. The Bronze Age in eastern parts of Central Asia. Ch. 13 (*Hist. Civiliz. Cent. Asia*, China, 1992).
41. The Henan Provincial Institute of Cultural Relics and Archaeology; The School of Archaeology and Museology, Excavation of the Longshan Period tombs on the Pingliangtai Site in Huaiyang county, Henan province (In Chinese). *Huaxia Kaogu* **4**, 14-27 (2019)
42. Discovery of the Late Shang dynasty cemetery in Nie village, Jiaozuo, Henan province (In Chinese). *Kaogu Yu Wenwu* **8**, 19-29 (2014).
43. Zhang, Q., Eng, J. T., Wei, J. & Zhu, H. Stable isotope analysis of human bone in Neolithic Age at Miaozigou site, Chayouqian Banner, Inner Mongolia (In Chinese). *Acta Anthr. Sin.* **29**, 270-275 (2010).
44. Wu, Q. *et al.* Outburst flood at 1920 BCE supports historicity of China's Great Flood and the Xia dynasty. *Science* **353**, 579–582 (2016).
45. Li, M., Sun, Z., Wang, L. & Hu, X. A preliminary study on human bones at Dacaozi site in Ping'an, Qinghai (In Chinese). *Chin. Cult. Relics* **7**, 1-2 (2014).
46. Shaanxi Provincial Institute of Archaeology, Shenmu County, The Shimao site in Shenmu County, Shaanxi (In Chinese). *Chinese Archaeology* **5**, 18–26 (2014).
47. Jeong, C. *et al.* The genetic history of admixture across inner Eurasia. *Nat. Ecol. Evol.* **3**, 966-976 (2019).
48. Jeong, C. *et al.* Bronze Age population dynamics and the rise of dairy pastoralism on the eastern Eurasian steppe. *Proc. Natl. Acad. Sci.* **115**, 11248–11255 (2018).
49. Kamberov, Y. G. *et al.* Modeling recent human evolution in mice by expression of a selected *EDAR* variant. *Cell* **152**, 691–702 (2013).
50. Kimura, R. *et al.* A common variation in *EDAR* is a genetic determinant of shovel-shaped incisors. *Am. J. Hum. Genet.* **85**, 528–535 (2009).
51. Yoshiura, K. *et al.* A SNP in the *ABCC11* gene is the determinant of human earwax type. *Nat. Genet.* **38**, 324–330 (2006).
52. Huerta-Sánchez, E. *et al.* Altitude adaptation in Tibetans caused by introgression of Denisovan-like DNA. *Nature* **512**, 194–197 (2014).
53. Xiang, K. *et al.* Identification of a Tibetan-specific mutation in the hypoxic gene *EGLN1* and its contribution to high-altitude adaptation. *Mol. Biol. Evol.* **30**, 1889–1898 (2013).

54. Jeong, C. *et al.* Admixture facilitates genetic adaptations to high altitude in Tibet. *Nat. Commun.* **5**, 3281 (2014).
55. Dong, G., Zhang, S., Yang, Y., Chen, J. & Chen, F. Agricultural intensification and its impact on environment during Neolithic Age in northern China. Chin. *Sci. Bull.* **61**, 2913–2925 (2016).
56. Chen, F. H. *et al.* Agriculture facilitated permanent human occupation of the Tibetan Plateau after 3600 B.P. *Science* **347**, 248–250 (2015).
57. Crawford, G. *et al.* Late Neolithic plant remains from Northern China: preliminary results from Liangchengzhen, Shandong. *Curr. Anthropol.* **46**, 309–317 (2005).
58. D'Alpoim Guedes, J. Adaptation and invention during the spread of agriculture to southwest China. Doctoral dissertation, Harvard University (Geography, 2013).
59. Guedes, J. d'Alpoim, Jiang, M., He, K., Wu, X. & Jiang, Z. Site of Baodun yields earliest evidence for the spread of rice and foxtail millet agriculture to south-west China. *Antiquity* **87**, 758–771 (2013).
60. Jin, G., Wagner, M., Tarasov, P. E., Wang, F. & Liu, Y. Archaeobotanical records of Middle and Late Neolithic agriculture from Shandong Province, East China, and a major change in regional subsistence during the Dawenkou Culture. *The Holocene* **26**, 1605–1615 (2016).
61. Qiu, Z., Shang, X., Ferguson, D. K. & Jiang, H. Archaeobotanical analysis of diverse plant food resources and palaeovegetation at the Zhumucun site, a late Neolithic settlement of the Liangzhu Culture in east China. *Quat. Int.* **426**, 75–85 (2016).
62. School of Archaeology and Museology, Peking University; Henan Provincial Institution of Culture Heritage and Archaeology. Archaeological discovery and research at the Wangchenggang site in Dengfeng. (Elephant Press, Hong Kong, 2007).
63. Chen, W., Zhang, J., Cai, Q. Analysis of plant remains excavated from Guchengzhai site in Xinmi, Henan Province (In Chinese). *Huaxia Kaogu.* **1**, 54-62 (2012).
64. Chen, X., Wang, L., Wang, Q. Analysis of flotation result of Xijincheng Site in Boai County, Henan Province from 2006 to 2007 (In Chinese). *Huaxia Kaogu*. **3**, 67-76 (2010).
65. Chen, X. *et al.* Analysis of sampling and flotation results of several pre-Qin sites in southeastern Shandong. East Asia Archaeology. *Science Press* **6**, 354-357 (2009).
66. Cheng, Z., Yang, Y., Yuan, Z. & Zhang, J. A Study on charred plant remains in Yangpu site of Suzhou, Anhui (In Chinese). *Jianghan Kaogu.* **1**, 95-103 (2016).
67. Cheng, Z., Yang, Y., Zhang, J. & Fang, F. Research on charred plant remains from Xiaosungang site in Huainan city, Anhui Province. *Quat. Sci.* **36**, 302-311 (2016).
68. Dai, Y. & Wan, J. Flotation result and analysis of Guiyuanqiao Site in Shifang city, Sichuan Province (In Chinese). *Sichuan Cult. Relics* **5**, 81-87 (2015).
69. Deng, Z. Analysis of plant remains excavated from Baligang Site in Dengzhou, Henan Province. (Graduate School of Archaeology and Museology, Peking University, Beijing, 2015).
70. Fan, X. J. Analysis of carbonized plant remains at Xichengyi site (In Chinese). (Graduate Institute of Cultural Heritage, Shandong University, Jinan, 2016).

71. Fu, P. & Sun, Y. Study on the ways of producing business at Haminmangha site: focusing on archaeobotanical analysis (In Chinese). *Nongye Kaogu.* **4**, 1-5 (2015).
72. Gao, S. *et al.* Flotation result and analysis of Zhaiyaoliang site in Yulin City, Shaanxi Province (In Chinese). *Nongye Kaogu.* **3**, 14-19 (2016).
73. Guo, X. The late period of Longshan in northern Shaanxi: a case study of plant and animal remains at Muzhuzhuliang and Shengedaliang sites (In Chinese). *Nongye Kaogu.* **3**, 19-23 (2017).
74. Guo, X. Analysis of prehistoric plant remains at Guangfulin site in Shanghai (In Chinese). (Graduate Institute of Cultural Heritage, Shandong University, Jinan, 2014).
75. Henan Provincial Institution of Culture Heritage and Archaeology. Nanjiaokou in Sanmenxia City (In Chinese). (Science Press, 2009).
76. Jiang, M. *et al.* Flotation result and preliminary study of Henglanshan Site in Xichang City in 2014 (In Chinese). *Chengdu Nongye Kaogu*. **6**, (2014).
77. Jin, G., Zhao, M., Sun, H. & Fang, H. Archaeobotanical investigation of Longshan cultural site in Chiping, Shandong Province (In Chinese). *Dongya Kaogu*. **6**, 317-320 (2009).
78. Jin, G., Wang, H., Yan, S. Study on carbonized plant remains of Longshan culture at Zhaojiazhuang site in Jiaozhou, Shandong Province (In Chinese). *Kaogu Kexue* **3**, 36-53 (2011).
79. Jin, G., Wang, C., Lan, Y. Analysis of carbonized plant remains at Xuejiazhuang site in Zhucheng (In Chinese). *Dongya Kaogu* **6**, 350-353 (2009).
80. Jin, G., Wang, C., Zhang, K. & Wang, Z. Archaeobotanical report of Fangjia site of Longshan culture in Zibo City (In Chinese). *Haidai Kaogu* **4**, 66-67 (2011).
81. Liu, C. & Fang, Y. Remaining analysis on unearthed plants of Wadian site in Yuzhou, Henan Province (In Chinese). *Nanfang Wenwu* **4**, 55-64 (2010).
82. Liu, H. *et al*. Preliminary analysis of plant remains excavated from Shenmingpu site in Xichuan, Henan Province (In Chinese). *Huaxia Kaogu*. **1**, 54-61 (2017).
83. Liu, X. Study on the plant remains excavated at the Case Board site in 2012 (In Chinese). (Graduate School of Culture heritage, Northwest University, Xian, 2014).
84. Henan Provincial Institution of Culture Heritage and Archaeology. Dadiwan In Qin'an (In Chinese). (Cultural Relics Press, 2006).
85. Zhong, H., Yang, Y., Shao, J. Zhao, Z., The research on the remains of the carbonized plant in the new street site, Lantian county, Shaanxi province (In Chinese). *Nanfang Wenwu* **3**, 36-43 (2015).
86. Zhong, H., Zhao, C., Wei, J., Zhao, Z. Floatation analysis and results of Xizhai site, Xinmi, Henan province (In Chinese). *Kaogu Yu Nongye* **1**, 21-29 (2016).
87. Institute of Archaeology, Chinese Academy of Social Sciences (In Chinese). Yuchi temple, Mengcheng County. (Science Press, 2017).
88. Zhao, Z., Jiang, L. Analysis on the plant remains by flotation unearthed in the Shangshan Site, Pujiang county, Zhejiang province (In Chinese). *Nanfang Wenwu* **3**, 109-130 (2016).

89. Zhao, Z. & He, N. Floatation results from the remains excavated at the Taosi site in 2002 (In Chinese). *Kaogu* **5**, 77-86 (2006).
90. Zhao, Z. & Fang, Y. Identification and analysis of the object floatation – selected from the soil samples collected to the Wangchenggang site in Dengfeng (In Chinese). *Huaxia Kaogu* **2**, 78-89 (2007).
91. Zhao, Z. & Chen, J. Flotation results and analysis of Yingpanshan site, Mao County, Sichuan Province (In Chinese). *Nanfang Wenwu* **3**, 60-67 (2011).
92. Yin, D. Analysis on the plant remains of the Huicun site, Bengbu city, Anhui province (In Chinese). (Graduate Institute of Archaeology, Chinese Academy of Social Sciences, 2011).
93. Yang, Y. The transition of human subsistence strategy and its influencing factors during prehistoric times in the Hexi Corridor, northwest China (In Chinese). (Graduate College of Earth and Environmental Sciences, Lanzhou University, 2015).
94. Rui, X., Zhou, Z., Jiang, M. Primary analysis of floatation remains in Zhonghaiguoji community, Chengdu (In Chinese). *Chengdu Kaogu Faxian* **00**, 240-250 (2012).
95. Rui, X., Jiang, M., Zuo, Z. Analysis of plant remains of primary excavation site from Huili and Huidong county, 2015 (In Chinese). *Chengdu Kaogu Faxian* **00**, 135-146 (2014).
96. Rui, X., Jiang, M., Liu, X. Analysis of plant remains from Guijiapu and Daozuomiao sites, Yanyuan County, 2015 (In Chinese). *Chengdu Kaogu Faxian* **00**, 147-159 (2014)
97. Wu. X. The research of plant remains in Longshan period from Dalaidian site in Henan (In Chinese). (Graduate School of History and Culture, Shandong University, 2016).
98. Wu, R., Cui, Y., Guo, R., Jin, G. Analysis of floatation remains from primary excavation results of Dashuigou site, 2015 (In Chinese). *Nongye Kaogu* **1**, 13-20 (2017).
99. Wu, C. Analysis of plant remains from Qinglongquan site in Yuan County, Hubei Province (In Chinese). (Graduate School of Chinese Academy of Social Science. 2011).
100. Wei, X., Kong, S., Yu, X. A preliminary study of the floral remains from the Shangpo site in Xiping, Henan (In Chinese). *Huaxia Kaogu* **3**, 77-84 (2007).
101. Wang, X. *et al.* Analysis of floatation remains from Nanshantou and Muwanghe site, Baishui River, Shaanxi Province (In Chinese). *Kaogu Yu Wenwu* **2**, 100-104 (2015).
102. Wang, H. Analysis of plant remains from Houyangguanzhuang site, Cangshan county (In Chinese). *Haidai Kaogu* **10**, 133-138 (2013).
103. Wang, H., Liu, Y., Jin, G. Analysis on the carbonized plant remains from the Dongpan site, 2009, Linshu County, Shandong (In Chinese). *Dongya Kaogu* **00**, 357-377 (2011).
104. Wang, C., Zhao, X., Jin, G. Analysis of the results of the floatation for the Liuzhuang site in Hebi, Henan (In Chinese). *Huaxia Kaogu* **3**, 90-99 (2010).
105. Tang, L., Luo, Y., Zhao, Z. Research on charred plant remains from the Chengzishan site, Ezhou (In Chinese). *Jianghan Kaogu* **2**, 108-115 (2017).
106. Tang, L., Huang, W., Guo, C., Qu, L. Research on the Neolithic agriculture in the region of the northwest of Hubei Province and the southwest of Henan Province from the floatation results of Dasi site, Yun County, Hubei Province (In Chinese). *Xibu Kaogu* **2**, 73-85 (2016)

107. Sun, Y., Cao, J., Jing, Z., Zhao, Z. Analysis on plant floatation remains from Weijiawopu site, 2009 (In Chinese). *Beifang Wenwu* **1**, 37-40 (2009).
108. Song, J. Analysis of plant remains from Tonglin site, Shandong province (In Chinese). (Graduate school of Chinese Academy of Social Science, 2007).
109. Sheng, P. *et al.* Preliminary research on the archaeobotanical remains at the Yangjiesha site in Hengshan, Shaanxi (In Chinese). *Kaogu Yu Wenwu* **3**, 123-128 (2017).
110. Liu, H. Results of soil samples flotation from two sites of Yangshao culture in Shaanxi and a comparative study (In Chinese). *Kaogu Yu Wenwu* **4**, 106-112 (2013).
111. Qiu, Z. *et al.* Analysis of plant remains at the Neolithic Yangjia Site, Wuxi City, Jiangsu Province. *Sci. China: Earth Sci.* **59**, 1803-1816 (2016).
112. Qi, W. *et al.* Analysis on the plant floatation remains of the prehistoric site in the upper Shu River region, Shandong (In Chinese). *Nongye Kaogu* **1**, 7-12 (2017).
113. Ma, Y. The research of carbonized plant remains of Wangjiacun site, Dalian (In Chinese). *Beifang Wenwu* **2**, 39-43 (2015).
114. Dong, G. *et al.* Agricultural intensification and its impact on environment during Neolithic Age in northern China. *Chin Sci Bull* **61**, 2913-2925 (2016)
115. Zhao, Z. The process of origin of agriculture in China: Archaeological evidence from flotation results (In Chinese). *Quat. Sci.* **5**, 73-84 (2014).
