# Supplementary material for: Ancient genomes from northern China suggest links between subsistence changes and human migration
Source: Nat Commun. 2020 Jun 1;11:2700. doi: 10.1038/s41467-020-16557-2 (PMC7264253; doi:10.1038/s41467-020-16557-2)
Supplement: Supplementary file 3 — Reporting Summary [file 41467_2020_16557_MOESM3_ESM.pdf]

nature research

Corresponding author(s): Choongwon Jeong

Last updated by author(s): Apr 15, 2020

Reporting Summary

Nature Research wishes to improve the reproducibility of the work that we publish. This form provides structure for consistency and transparency in reporting. For further information on Nature Research policies, see Authors & Referees and the Editorial Policy Checklist .

Statistics

For all statistical analyses, confirm that the following items are present in the figure legend, table legend, main text, or Methods section.

n/a	Confirmed	
☐	☒	The exact sample size (*n*) for each experimental group/condition, given as a discrete number and unit of measurement
☒	☐	A statement on whether measurements were taken from distinct samples or whether the same sample was measured repeatedly
☐	☒	The statistical test(s) used AND whether they are one- or two-sided<br>*Only common tests should be described solely by name; describe more complex techniques in the Methods section.*
☒	☐	A description of all covariates tested
☒	☐	A description of any assumptions or corrections, such as tests of normality and adjustment for multiple comparisons
☐	☒	A full description of the statistical parameters including central tendency (e.g. means) or other basic estimates (e.g. regression coefficient) AND variation (e.g. standard deviation) or associated estimates of uncertainty (e.g. confidence intervals)
☐	☒	For null hypothesis testing, the test statistic (e.g. *F*, *t*, *r*) with confidence intervals, effect sizes, degrees of freedom and *P* value noted<br>*Give P values as exact values whenever suitable.*
☒	☐	For Bayesian analysis, information on the choice of priors and Markov chain Monte Carlo settings
☒	☐	For hierarchical and complex designs, identification of the appropriate level for tests and full reporting of outcomes
☒	☐	Estimates of effect sizes (e.g. Cohen's *d*, Pearson's *r*), indicating how they were calculated

Our web collection on statistics for biologists contains articles on many of the points above.

Software and code

Policy information about availability of computer code

Data collection	Illumina sequence data were processed using the following programs to obtain genotype data used in the analysis: AdapterRemoval v2.2.0, BWA v0.7.12, DeDup v0.12.2, bamUtils v1.0.13, pileupCaller (https://github.com/stschiff/sequenceTools), mapDamage v2.0.6, ANGSD v0.910, Schmutzi v1.5.1. These programs are publicly available.
Data analysis	Population genetic data analysis in this study was performed using the following publicly available programs: Geneious v11.1.3, HaploGrep2, smartpca v16000, ADMIXTURE v1.3.0, PLINK v1.90, lcMLkin v0.5.0, qp3Pop v435, qpDstat v755, qpWave v410, qpAdm v810, continuity test (https://github.com/Schraiber/continuity). Non-default parameters used in our analysis are described in the Methods section. The base map in Figure 1 was generated by ArcGIS v9.2. Calibration of AMS 14C dating results was done by either Calib v6.0.1 or OxCal v4.2.3.

For manuscripts utilizing custom algorithms or software that are central to the research but not yet described in published literature, software must be made available to editors/reviewers. We strongly encourage code deposition in a community repository (e.g. GitHub). See the Nature Research guidelines for submitting code & software for further information.

Data

Policy information about availability of data

All manuscripts must include a data availability statement. This statement should provide the following information, where applicable:

- Accession codes, unique identifiers, or web links for publicly available datasets
- A list of figures that have associated raw data
- A description of any restrictions on data availability

Raw FastQ and alignment files (BAM format) are available at the European Nucleotide Archive (ENA) under the accession number PRJEB36297. Haploid genotype data of ancient individuals in this study on the 1240k panel are available in the EIGENSTRAT format from the following link: [https://edmond.mpdl.mpg.de/imeji/collection/5oV1TtHlsYggGBT3].

Field-specific reporting

Please select the one below that is the best fit for your research. If you are not sure, read the appropriate sections before making your selection.

☐ Life sciences ☐ Behavioural & social sciences ☒ Ecological, evolutionary & environmental sciences

For a reference copy of the document with all sections, see nature.com/documents/nr-reporting-summary-flat.pdf

Ecological, evolutionary & environmental sciences study design

All studies must disclose on these points even when the disclosure is negative.

Study description	This study includes whole genome sequencing of 55 ancient individuals from northern China, out of 107 skeletal elements screened, ranging between 7500 and 1800 years before present. Sequencing coverage ranges 0.03-7.53x. Ancient genomes come from different regions: Amur River (n=6), West Liao River (n=10), Yellow River (n=22), and Yellow River periphery (n=17).
Research sample	Research samples are composed of 55 ancient genomes from various archaeological sites in northern China. They are chosen to cover major archaeological cultures and time periods in northern China. We separated them into broad analysis units based on their geographic origin, time period, and individual genetic profiles. The analysis units include: AR_EN (n=2), AR_IA (n=1), AR_Xianbei_IA (n=3), HMMH_MN (n=1), WLR_MN (n=3), WLR_LN (n=3), WLR_BA (n=2), WLR_BA_o (n=1), Miaozigou_MN (n=3), Shimao_LN (n=3), Upper_YR_LN (n=7), Upper_YR_IA (n=4), YR_MN (n=8), YR_LN (n=8), YR_LBIA (n=6).
Sampling strategy	No sample-size selection was performed prior to the study. To produce ancient genomes reported in this study, we screened the accessible skeletal elements from the relevant geographic regions and time periods, and produced in-depth sequencing data for those with sufficient endogenous DNA preservation and without substantial contamination.
Data collection	Sequencing of the libraries was performed on an Illumina HiSeq X10 instrument at the Annoroad Company, China, in the 150-bp paired-end sequencing design.
Timing and spatial scale	Laboratory works and sequencing was conducted over the period from February 2017 to December 2018. Samples were taken from various archaeological sites in northern China, the locations of which are provided in Fig. 1 and Supplementary Data 1.
Data exclusions	We excluded samples only if the samples do not meet the quality criteria, either by having low level of endogenous human DNA prohibiting genome-scale sequencing or by showing high level of contamination estimates. For population genetic analysis that requires exclusion of genetic relatives, we excluded closely related individuals (1st degree relatives) by removing one with lower coverage from each pair.
Reproducibility	We took multiple individuals from each archaeological site, if available, to support the representativeness of their genetic profiles. For each sample, we estimated contamination level to support the authenticity of data.
Randomization	Ancient genomes were first analyzed by each individual, and then were allocated into the analysis group based on their archaeological context, absolute date (14C dating), and their individual genetic profile. Randomization is not applicable.
Blinding	There was no experimental treatment of samples involved in this study that requires blinding. Data analysis was performed based on the analysis groups that were defined by external information (archaeological context and date).

Did the study involve field work? ☐ Yes ☒ No

Reporting for specific materials, systems and methods

We require information from authors about some types of materials, experimental systems and methods used in many studies. Here, indicate whether each material, system or method listed is relevant to your study. If you are not sure if a list item applies to your research, read the appropriate section before selecting a response.

Materials & experimental systems

n/a	Involved in the study	
☒	☐	Antibodies
☒	☐	Eukaryotic cell lines
☒	☐	Palaeontology
☒	☐	Animals and other organisms
☒	☐	Human research participants
☒	☐	Clinical data

Methods

n/a	Involved in the study	
☒	☐	ChIP-seq
☒	☐	Flow cytometry
☒	☐	MRI-based neuroimaging
