# Supplementary material for: Ancient genomes from northern China suggest links between subsistence changes and human migration
Source: Nat Commun. 2020 Jun 1;11:2700. doi: 10.1038/s41467-020-16557-2 (PMC7264253; doi:10.1038/s41467-020-16557-2)
Supplement: Supplementary file 4 — Description of Additional Supplementary Files [file 41467_2020_16557_MOESM4_ESM.pdf]

Description of Additional Supplementary Files

File name: Supplementary Data 1
Description: A summary of all the ancient samples screened in this study.

File name: Supplementary Data 2
Description: A list of 92 ancient groups used for the population genetic analyses in this study.

File name: Supplementary Data 3
Description: A list of 161 present-day populations used for the population genetic analyses in this study
