# Supplementary material for: Ancient genomes from northern China suggest links between subsistence changes and human migration
Source: Nat Commun. 2020 Jun 1;11:2700. doi: 10.1038/s41467-020-16557-2 (PMC7264253; doi:10.1038/s41467-020-16557-2)
Supplement: Supplementary file 2 — Peer Review File [file 41467_2020_16557_MOESM2_ESM.pdf]

Editorial Note: Parts of this peer review file have been redacted as indicated to remove third-party material where no permission to publish could be obtained.

Reviewers' comments:

Reviewer #1 (Remarks to the Author):

In "Genomic History of Northern China for the last 7,500 years" by Ning et al., the authors generated 55 new ancient DNA data from Northern China between ~2000 to 7500 years before present. The newly generated dataset is not large by today's standard in aDNA, but is unique due to the general rarity of aDNA data from China. Customary to aDNA papers authored by these authors, I thought the questions and the interpretation and insights from the analysis of these aDNA were solid; I do not have major concerns with the analysis, though I have some detailed comments that I will describe below. I am also not an archaeologist/ anthropologist, so some of the interpretation in the manuscript could benefit from a reviewer of that background. My main concern is perhaps not scientific: the aDNA field is very open to data sharing after the paper is published. For this manuscript, the cited accession number on ENA did not reach the dataset:

[redacted]

If the dataset is not to become publicly available, this would severely compromise the impact of this manuscript and change my view of the publishability of the manuscript.

Comments:

1. In Figure 2, the choice of using the 1240k-Illumina dataset did not clearly illustrate the main point the authors were trying to make. I understand that Sherpa and Tibetan samples became useful for later analyses, but the Asia PCA (Figure 2B) to me did not significantly improve the resolution of the global PCA (Figure 2A), because in both cases PC1 is driven strongly by some outgroup. I would like to see PCAs without the Sherpa/Tibetan and/or lower PCs be presented for finer-scale insights. For example, perhaps removing the Sherpa and Tibetans would show clearer differentiation of YR individuals over time?
2. Evidence for genomic continuity among the AR individuals is relatively weak. I failed to see how the outgroup-f3 is supposed to argue for continuity. The "large" f3 values have no basis for comparison. There are known tests of population continuity (e.g. Schraiber, Genetics 2018) that can be applied in this case.
3. Lines 106-120: the general narrative is that there is a transition from MN to LN with input from SC-SEA and a pause between LN to LBIA. But present day Han Chinese showed additional input from SC-SEA. The authors attributed this to northward expansion of rice farming. Because there is a large gap in sampled individuals between LBIA and present day, could this pattern be due to the much more recent migration northward in the late 19th-20th century (which was driven by political changes or regulations, rather than by farming)?

4. I generally find the outgroup-f3 statistics to be uninformative. In Figure S10, nearly all comparisons are grouped together with very close mean values and wide standard errors, thus providing no valuable information. Perhaps the authors can consider using the matrix of outgroup f3 statistics to cluster individuals (like Fu et al. Nature 2016 did), but otherwise I do not see much use of them.

5. For example, in line 124-126 outgroup f3 statistics was one of the evidence to show that
   Shimao_LN and Miaozigou_MN are genetically similar to each other. That is just based on the two populations having the largest outgroup f3 statistics, but are they significantly different from others? This assertion should be tested directly with a f4 or D test. (I also beg to differ that on PCA in Figure 2 these two populations are similar to each other. They are as separated as YR_MN from Upper_YR_IA.)

Minor comments:

1. Figure 1 – it would be helpful to also label the actual river in addition to the river valleys.

2. I also wish the labels assigned to Figure 1 and in PCA be improved. As far as I can tell it is a random combination of symbol and color assigned to each population, which makes it difficult for a colorblind reader to match the symbols in the PCA to the population label in the spatiotemporal graph of Figure 1. It would be nice to have some patterns, such as the same valley given similar shades of the same color, or use the same symbol.

Reviewer #2 (Remarks to the Author):

I feel that the authors should rethink the title. The current one is purely descriptive and does not tell us very much at all about the manuscript in the sense of why the work was done, whether the paper is testing a particular hypothesis etc? I suggest too that the authors might expand the first paragraph after the Abstract on page 2 to include material from the Supplementary sections S1 and S1.1. In my view the authors need to emphasise the argument that this region is one of the first regions in world prehistory where agriculture and complex societies evolved. This reader did not believe this before reading Peterson, et al. 2010.

Notwithstanding the above comment, the authors are to be congratulated in writing a very succinct manuscript. Nevertheless I will list a number of possible changes which I hope might help the reader. Please find these below.

Abstract page 2 line – I have no idea what "genetic development and interactions" actually means.
Page 3 – line 87 – they fall ON different.
Page 3 line 106 –"… but away from the AR cluster". You need to explain this more carefully.
Page 3 line 120 – "that may BE related".
Page 5 line 177 – "… may have led TO an influx of people …"
Page 5 line 181 -182 -sentence 181 – 182. This is not a sentence – this section needs repair or rewriting.
Page 180 – Again, I have no idea what dynamic genetic changes mean? I assume the authors are simply saying that there are changes of time (i.e. last six millennia"). This word is used throughout and I have the same concern about each of these e.g. on Pag2 1 line 48; pag4 line 146. In each case it, the word means precisely nothing.

Figure 1 is a real problem. In particular it is very difficult to interpret. The background is too dark and

consequently the shading is very unclear. Also, I wonder if there should be some landmark geographical features, cities, or the like that would help the reader their marks.

Figure 1 legend “Two published Early Neolithic genomes …” No reference is given.
Figure 1 legend. “Two published (no reference) Early Neolithic genomes …”.
Some sentences are not complete.

Figure 2A in particular, and 2B in part, is not legible in most parts because of the colours.
A is used in some places and a in others – the same applies to B/b.
The PCA results presented in 2A and 2B are quite problematic since they are not legible.

The supplementary is also very well constructed. It was informative and a pleasure to read.

Throughout reading this ms I kept feeling that there should be a direct comparison of linguistic with genomic changes. I notice that Malaspinas et al. 2016 (Nature doi:10.1038/nature18299) did this and showed that the typologies of the trees were almost identical, although the basal dates of divergence were markedly different, presumably since linguistic changes occur much faster (on average) than genomic ones. Of course, I appreciate that the big problem here is that linguists do not have a molecular clock. But the message in Malispinas et al. was that genomes and language evolve together. Still, it would be very interesting if the authors could conduct a similar analysis.

Reviewers' comments:

Reviewer #1 [Remarks to the Authors]:

In "Genomic History of Northern China for the last 7,500 years" by Ning et al., the authors generated 55 new ancient DNA data from Northern China between ~2000 to 7500 years before present. The newly generated dataset is not large by today's standard in aDNA, but is unique due to the general rarity of aDNA data from China. Customary to aDNA papers authored by these authors, I thought the questions and the interpretation and insights from the analysis of these aDNA were solid; I do not have major concerns with the analysis, though I have some detailed comments that I will describe below. I am also not an archaeologist/ anthropologist, so some of the interpretation in the manuscript could benefit from a reviewer of that background. My main concern is perhaps not scientific: the aDNA field is very open to data sharing after the paper is published. For this manuscript, the cited accession number on ENA did not reach the dataset. If the dataset is not to become publicly available, this would severely compromise the impact of this manuscript and change my view of the publishability of the manuscript. If the dataset is not to become publicly available, this would severely compromise the impact of this manuscript and change my view of the publishability of the manuscript.

We deeply appreciate the reviewer's positive assessment on our works and sharing of enthusiasm for the genomic study of ancient China. We firmly agree with the reviewer that data from this study should be made publicly available upon publication and we apologize for our providing an invalid accession number. Our data, both raw FastQ files and alignment data in BAM format, are now uploaded to the European Nucleotide Archive (ENA) under the project accession number PRJEB36297. For the full reproducibility of the analyses we performed in this study, we will also make haploid genotype data of the 55 ancient individuals on the 1240k panel from the Edmond data repository of the Max Planck Society (https://edmond.mpdl.mpg.de/imeji/collection/5oV1TtHlsYggGBT3). The data will be made publicly available as soon as the manuscript is published. Public deposition of the above raw data is supported by the formal permission from the Chinese Ministry of Science and Technology (Accession number: 2020BAT005).

1. In Figure 2, the choice of using the 1240k-Illumina dataset did not clearly illustrate the main point the authors were trying to make. I understand that Sherpa and Tibetan samples became useful for later analyses, but the Asia PCA (Figure 2B) to me did not significantly improve the resolution of the global PCA (Figure 2A), because in both cases PC1 is driven strongly by some outgroup. I would like to see PCAs without the Sherpa/Tibetan and/or lower PCs be presented for finer-scale insights. For example, perhaps removing the Sherpa and Tibetans would show clearer differentiation of YR individuals over time?

We appreciate the reviewer's careful look into the PCA results. Following the reviewer's suggestion, we tried different sets of East Asians for calculating PCs to improve our East Asian PCA results. We provide a new East Asian PC1 vs PC2 plot using the 1240k-Illumina data (Fig. 2C), including the following eight populations: Han, Hezhen, Japanese, Naxi, Oroqen, Tibetan, Tujia, Xibo, Yi. We removed Sherpa according to the reviewer's suggestion, but kept Tibetans and accordingly the 1240k-Illumina data set in our analysis. The location of ancient individuals on this plot matches well with the key findings we report with f4 statistics and qpAdm results:

i) *YR_LN and YR_LBIA individuals are distinct from YR_MN individuals and show more affinity with the present-day SC-SEA populations (represented by Han and Tujia here).*
ii) *Miaozigou_MN, Shimao_LN, Upper_YR_LN, Upper_YR_IA individuals are distinct from the YR individuals and show more affinity with the present-day Tibetans.*

We believe that this modification suffices the reviewer's request. Lower PCs from the Eurasian or East Asian PCA did not improve the distinction between the YR populations (data not shown).

2. Evidence for genomic continuity among the AR individuals is relatively weak. I failed to see how the outgroup-f3 is supposed to argue for continuity. The "large" f3 values have no basis for

comparison. There are known tests of population continuity (e.g. Schraiber, Genetics 2018) that can be applied in this case.

We agree with the reviewer that the "large" outgroup-f3 only shows a general close relationship between populations but not continuity. In the previous version of the manuscript, we already presented the f4 symmetry test of the form f4(AR1, AR2; X, Mbuti) for each pair of AR populations (AR1 and AR2) against 20 representative groups outside of the AR (X's). We obtained in general non-significant results (|Z|<3; Figs. S10-S11) formally supporting that the AR populations are cladal to the exclusion of representative ancient and present-day populations outside of the region. Following the reviewer's suggestion, we updated our manuscript in the following ways:

- *i) We modified the main text to clarify our interpretation of outgroup-f3 and f4 symmetry results (the "strong genetic continuity of AR populations over the last 7,500 years" section)*
- *ii) We provide a heatmap of outgroup-f3 statistics for all pairs of AR populations to highlight the similarity of the AR populations (new SI Fig. S8)*
- *iii) We provide the qpWave test of cladality between the ancient and present-day AR populations (new SI Table S3).*
- *iv) We applied the continuity test described by Schraiber 2018 to the same pairs of ancient and present-day AR populations. Although they make a clade in the qpWave analysis, the continuity test rejects the ancestor-descendant relationship between them (new SI Table S3).*

Reflecting the new results in iv), we changed our wording from "continuity" to "stability" or "cladality" to highlight the lack of genetic changes associated with the gene pools distinct from the AR one. Also, we added a sentence in the main text to describe and interpret the continuity test results.

3. Lines 106-120: the general narrative is that there is a transition from MN to LN with input from SC-SEA and a pause between LN to LBIA. But present day Han Chinese showed additional input from SC-SEA. The authors attributed this to northward expansion of rice farming. Because there is a large gap in sampled individuals between LBIA and present day, could this pattern be due to the much more recent migration northward in the late 19th-20th century (which was driven by political changes or regulations, rather than by farming)?

We agree with the reviewer that there is a large gap between Iron Age and present-day Han Chinese. However, we believe it unlikely that the historical migration during the late 19th-20th century explains the observed difference between YR_LBIA and present-day Han Chinese: the suggested historical migration was a northward migration of YR populations into Manchuria, which harbors parts of the Amur River and West Liao River Valleys in northeast China. It does not involve a gene flow between YR and SC-SEA populations that is necessary to create the observed signal. We would like to highlight that genetic data of Han Chinese individuals we used in this study only represents a small fraction of genetic diversity across present-day Han Chinese, an ethno-cultural group rather than a homogenous genetic group. The formation of Han Chinese since the Iron Age presumably involved admixture with and integration of nearby groups who may have a genetic profile similar to present-day SC-SEA populations, which will be a topic of great interest for future genetic studies.

4. I generally find the outgroup-f3 statistics to be uninformative. In Figure S10, nearly all comparisons are grouped together with very close mean values and wide standard errors, thus providing no valuable information. Perhaps the authors can consider using the matrix of outgroup f3 statistics to cluster individuals (like Fu et al. Nature 2016 did), but otherwise I do not see much use of them

Following the reviewer's suggestion, we now present a heatmap representation of the matrix of outgroup-f3 statistics between East Asian populations (SI Fig. S9). It is now more visible that the AR populations form a tight cluster.

5. For example, in line 124-126 outgroup f3 statistics was one of the evidences to show that Shimao_LN and Miaozigou_MN are genetically similar to each other. That is just based on the two populations having the largest outgroup f3 statistics, but are they significantly different from others?

This assertion should be tested directly with a f4 or D test. (I also beg to differ that on PCA in Figure 2 these two populations are similar to each other. They are as separated as YR_MN from Upper_YR_IA.)

Following the reviwer's suggestion, we now provide f4 symmetry test results for Miaozigou_MN, Shimao_LN, Upper_YR_LN (new SI Fig. S18). We find almost no significant difference between these populations (max |Z| = 3.2 SE), supporting the genetic similarity between them.

Minor comments:

1. Figure 1 – it would be helpful to also label the actual river in addition to the river valleys.
Done.

2. I also wish the labels assigned to Figure 1 and in PCA be improved. As far as I can tell it is a random combination of symbol and color assigned to each population, which makes it difficult for a colorblind reader to match the symbols in the PCA to the population label in the spatiotemporal graph of Figure 1. It would be nice to have some patterns, such as the same valley given similar shades of the same color, or use the same symbol.
Following the reviewer's suggestion, we changed the color-symbol combinations in a consistent way, in which we use one symbol per river valley and one color per time period (MN, LN, LB, IA). We applied the same combination for the map and PCA figures.

Reviewer #2 [Remarks to the Authors]:

I feel that the authors should rethink the title. The current one is purely descriptive and does not tell us very much at all about the manuscript in the sense of why the work was done, whether the paper is testing a particular hypothesis etc? I suggest too that the authors might expand the first paragraph after the Abstract on page 2 to include material from the Supplementary sections S1 and S1.1. In my view the authors need to emphasise the argument that this region is one of the first regions in world prehistory where agriculture and complex societies evolved. This reader did not believe this before reading Peterson, et al. 2010.

Following the reviewer's suggestion, we added descriptions for the Hongshan culture and the early complex societies in northern China in the Introduction. We also changed our title to "Ancient genomes from northern China suggest links between subsistence changes and human migration" to highlight the major findings in our study.

Notwithstanding the above comment, the authors are to be congratulated in writing a very succinct manuscript. Nevertheless I will list a number of possible changes which I hope might help the reader. Please find these below.

Abstract page 2 line – I have no idea what "genetic development and interactions" actually means.
We changed it to "genetic history" to make the sentence concise and clear.

Page 3 – line 87 – they fall ON different.
Corrected.

Page 3 line 106 –"… but away from the AR cluster". You need to explain this more carefully.
We modified the sentence to "Ancient YR individuals from the Central Plain area form a cluster distinct from the AR individuals in the PCA and likewise share a similar genetic profile in the ADMIXTURE analysis".

Page 3 line 120 – "that may BE related".

Corrected.

Page 5 line 177 – "… may have led TO an influx of people …"
Corrected.

Page 5 line 181 -182 -sentence 181 – 182. This is not a sentence – this section needs repair or rewriting.
Corrected.

Page 180 – Again, I have no idea what dynamic genetic changes mean? I assume the authors are simply saying that there are changes of time (i.e. last six millennia"). This word is used throughout and I have the same concern about each of these e.g. on Pag2 1 line 48; pag4 line 146. In each case it, the word means precisely nothing.
We used "dynamic genetic changes" to indicate the opposite direction of genetic changes in the WLR during MN-to-LN and LN-to-EBA transitions. Following the reviewer's suggestion, we changed "dynamic" to "frequent" or "substantial".

Figure 1 is a real problem. In particular it is very difficult to interpret. The background is too dark and consequently the shading is very unclear. Also, I wonder if there should be some landmark geographical features, cities, or the like that would help the reader their marks.
Following the reviewer's suggestion, we modified Fig. 1 to brighten the background and to include major rivers and cities. See also our replies to the reviewer #1.

Figure 1 legend "Two published Early Neolithic genomes …" No reference is given.
Figure 1 legend. "Two published (no reference) Early Neolithic genomes …".
Some sentences are not complete.
We added the references and fixed the sentences in the Fig. 1 legend.

Figure 2A in particular, and 2B in part, is not legible in most parts because of the colours. A is used in some places and a in others – the same applies to B/b. The PCA results presented in 2A and 2B are quite problematic since they are not legible.
We modified Fig. 2 to make it more accessible. Please also see our replies to the reviewer #1.

The supplementary is also very well constructed. It was informative and a pleasure to read.
We appreciate the reviewer's positive comment.

Throughout reading this ms I kept feeling that there should be a direct comparison of linguistic with genomic changes. I notice that Malaspinas et al. 2016 (Nature doi:10.1038/nature18299) did this and showed that the typologies of the trees were almost identical, although the basal dates of divergence were markedly different, presumably since linguistic changes occur much faster (on average) than genomic ones. Of course, I appreciate that the big problem here is that linguists do not have a molecular clock. But the message in Malispinas et al. was that genomes and language evolve together. Still, it would be very interesting if the authors could conduct a similar analysis.

We appreciate the reviewer's suggestion. We agree with the reviewer that the interdisciplinary comparison is an important task to be performed in the future, but we think that it is a task beyond the scope of our current study for the following reasons. The case study suggested by the reviewer compares the linguistic and genetic phylogenies of Aboriginal Australian groups, who collectively experienced tens of thousands of years of isolation from the populations outside of Sahul. Therefore, their genetic relationship can be relatively robustly represented by a phylogenetic tree. In stark contrast, the genetic history of Transeurasian and to a lesser degree Sino-Tibetan populations is characterized by pervasive admixture with neighboring populations speaking different languages as well as by integration of other populations by language conversion. Therefore, at this stage, it is an active area of study to genetically test if Transeurasian and/or Sino-Tibetan populations share a common genetic substratum and to reconstruct an accurate phylogeny of this substratum accounting

for complex and extensive admixture events. We now explicitly call for future studies for this topic at the end of our Discussion section.

REVIEWERS' COMMENTS:

Reviewer #1 (Remarks to the Author):

I'd like to thank the authors for addressing my comments. I have no further major concerns, and only a few minor questions/comments:

1. line 231: 'novel large-scale survey' sounded awkward. Not to diminish the work presented in this manuscript, but a method could be novel, a finding could be novel, but I'm not sure a survey would be. I might just remove the word novel. The novelty of the dataset is significantly acknowledged in the following sentence by providing the 'first genomic look'.

2. line 249: please clarify the meaning of 'candidate sources'.

3. The supplement should be proof-read thoroughly. I find at least one instance where a wrong figure was referred to (line 626 should be referring to Figure S12), presumably due to re-numbering / new figures in the revision.

Reviewer #2 (Remarks to the Author):

Thank you to the authors for considering my comments in a productive manner. I think the revised manuscript is a significant improvement. Congratulations to the authors.

Reviewer #3 (Remarks to the Author):

This study presents the first broad investigation of the whole genome of ancient people in China, and will at least provide many basic data, which will be very important for the ensuing aDNA research. They describe the chronological variation of genetic profile in AR, WLR and YR and show some meaningful explanation about human migration, subsistence strategy. Thus, the study deserves publication after minor revision. Some advise are as follows:
1) Page 3 Line 113, Fig. 1 A-C should be Fig. 2 A-C. In Fig.2 C the letters for Han, Tibet and so on are too small.
2) Page 4 Line 119, Fig.S2 A-B should be Fig.S5. So all the codes of Fig.S are in disorder?
3) Page 5 Line 168-174, the authors mentioned that ancient northern Chinese show extra affinity with (modern) SC-SEA populations, and they explained that ancient people from south China expanded northward with rice farming. Here I don't agree this opinion. As known, many north Chinese moved to the Yangtze River basin between West-Jin and Song Dynasty. Namely, ancient north Chinese contribute to the gene pool of the modern southern Chinese; so it's common that ancient northern Chinese show extra affinity with (modern) SC-SEA populations.
4) domesticate ruminant animals including cattle and sheep were introduced into northern China in the late Neolithic or early Bronze Age, and the possible route is from Mongolia to north or northeast China (Yang, 2020). Whether did this progress involve human migration? the author should compare ancient YR or AR population with ancient Mongolia population (Jeong et al., 2018) in the bronze Age to answer this question. Namely, the authors should judge or clearly suggest whether there is some genetic like between Bronze Age Mongolian and contemporary WLR and YR population.
5) In the subsistence strategy discussion, milking and lactase persistence are also hot research about pastoralism society. Did the authors find some evidence of it in the Bronze Age YR, WLR or AR people?

Reference:
1) Yang Y. Dairying transformed Mongolia[J]. Nature Ecology & Evolution, 2020, 4(3): 288-289.
2) Jeong C, Wilkin S, Amgalantugs T, et al. Bronze Age population dynamics and the rise of dairy pastoralism on the eastern Eurasian steppe[J]. Proceedings of the National Academy of Sciences,

2018, 115(48): E11248-E11255.

REVIEWERS' COMMENTS:

Reviewer #1 (Remarks to the Author):

I'd like to thank the authors for addressing my comments. I have no further major concerns, and only a few minor questions/comments:

1. line 231: 'novel large-scale survey' sounded awkward. Not to diminish the work presented in this manuscript, but a method could be novel, a finding could be novel, but I'm not sure a survey would be. I might just remove the word novel. The novelty of the dataset is significantly acknowledged in the following sentence by providing the 'first genomic look'.
We have changed the text following the reviewer's suggestion.

2. line 249: please clarify the meaning of 'candidate sources'.
By 'candidate sources' we referred to candidate source populations that may have brought rice farming into the Central Plain, if it was associated with demic diffusion. We modified the sentence to clarify this.

3. The supplement should be proof-read thoroughly. I find at least one instance where a wrong figure was referred to (line 626 should be referring to Figure S12), presumably due to re-numbering / new figures in the revision.
We appreciate the reviewer to catch this mistake. We have corrected it and carefully checked the remainder of SI and corrected for such mistakes to make sure that all the figures/tables are referred correctly.

Reviewer #2 (Remarks to the Author):

Thank you to the authors for considering my comments in a productive manner. I think the revised manuscript is a significant improvement. Congratulations to the authors.
We deeply appreciated for the referee's extensive suggestions in improving the quality of the paper.

Reviewer #3 (Remarks to the Author):

This study presents the first broad investigation of the whole genome of ancient people in China, and will at least provide many basic data, which will be very important for the ensuing aDNA research. They describe the chronological variation of genetic profile in AR, WLR and YR and show some meaningful explanation about human migration, subsistence strategy. Thus, the study deserves publication after minor revision. Some advise are as follows:

1) Page 3 Line 113, Fig. 1 A-C should be Fig. 2 A-C. In Fig.2 C the letters for Han, Tibet and so on are too small.
We appreciate the reviewer for pointing our mistake out. We have made the appropriate correction. Following the reviewer's suggestion, we increased the size of the labels for present-day individuals in Fig. 2C to make them more easily recognizable.

2) Page 4 Line 119, Fig.S2 A-B should be Fig.S5. So all the codes of Fig.S are in disorder?
The part mentioned by the reviewer is 'Figs. 2A-B', not 'Fig. S2A-B'. The main figures 2A-B are correctly referred to here. We have checked the main text and SI to make sure that the figure and table numbers are referred to in the correct positions.

3) Page 5 Line 168-174, the authors mentioned that ancient northern Chinese show extra affinity with (modern) SC-SEA populations, and they explained that ancient people from south China expanded northward with rice farming. Here I don't agree this opinion. As known, many north Chinese moved

to the Yangtze River basin between West-Jin and Song Dynasty. Namely, ancient north Chinese contribute to the gene pool of the modern southern Chinese; so it's common that ancient northern Chinese show extra affinity with (modern) SC-SEA populations.

We thank for the reviewer's careful suggestion. Although we in principle agree with the reviewer that the f_4 statistics per se do not show the direction of gene flow, we believe that the reviewer's suggestion of the historical migration since West-Jin is unlikely to explain the genetic difference between Middle and Late Neolithic YR populations. This is based on the fact that we have a time series data in the YR region, especially the Late Bronze and Iron Age individuals (YR_LBIA). As reported in the main text, we do not see the genetic difference between YR_LN and YR_LBIA in terms of their affinity with SC-SEA. If the signal were solely driven by the later historical migration, we should have observed higher affinity of more recent (e.g. YR_LBIA) populations in comparison to earlier ones (e.g. YR_LN). Present-day Han Chinese again show much higher affinity to SC-SEA than YR_LBIA do: we believe that a substantial part of this signal, but not the earlier one between YR_MN and YR_LN, comes from the historical migration the reviewer pointed out.

4) domesticate ruminant animals including cattle and sheep were introduced into northern China in the late Neolithic or early Bronze Age, and the possible route is from Mongolia to north or northeast China (Yang, 2020). Whether did this progress involve human migration? the author should compare ancient YR or AR population with ancient Mongolia population (Jeong et al., 2018) in the bronze Age to answer this question. Namely, the authors should judge or clearly suggest whether there is some genetic like between Bronze Age Mongolian and contemporary WLR and YR population.

We thank for the reviewer's suggestions. Actually, we had already included the Bronze Age Mongolia individuals (Khövsgöl) in Jeong 2018 in our analyses. However, in our analyses, we did not find a genetic support that Khövsgöl had contributed to the formation of the Bronze Age WLR or YR populations. Considering the limited availability of ancient genomes from Inner Mongolia, southern Mongolia, and/or Xinjiang, we believe that future studies focusing on these regions are critical to properly investigate this important question.

5) In the subsistence strategy discussion, milking and lactase persistence are also hot research about pastoralism society. Did the authors find some evidence of it in the Bronze Age YR, WLR or AR people?

We investigated 8 SNPs known to be associated with lactase persistence for the YR, WLR and AR individuals, but did not find any derived allele for lactase persistence (Supplementary Table 11). We would like to highlight that the lack of lactase persistence allele does not mean the absence of milk/dairy consumption in these individuals, considering the example described in Jeong et al 2018. We have not performed the proteomic analysis of dental calculus for these individuals, which is a topic for a separate study.
